# Proline Ring‐Inversion Dynamics in Peptides Enable Cross‐Relaxation‐Based Dynamic Nuclear Polarization

**DOI:** 10.1002/cphc.70444

**Published:** 2026-06-14

**Authors:** Florian Taube, Max Gierth, Georg Künze, Björn Corzilius

**Affiliations:** ^1^ Institute of Chemistry and Department Life Light & Matter University Rostock Rostock Germany; ^2^ Institute for Drug Discovery Faculty of Medicine Leipzig University Leipzig Germany; ^3^ Interdisciplinary Center for Bioinformatics Leipzig University Leipzig Germany; ^4^ Center for Scalable Data Analytics and Artificial Intelligence Leipzig University Leipzig Germany; ^5^ Leibniz Institute of Catalysis (LIKAT) e.V. Rostock Germany

**Keywords:** amino acids, proline, puckering dynamics, SCREAM‐DNP

## Abstract

Dynamic nuclear polarization (DNP) is commonly used to uniformly enhance magic–angle spinning (MAS) NMR signals, but recent efforts have focused on achieving site‐specific enhancement to address severe spectral crowding in large biomolecular systems. One promising strategy is specific cross–relaxation enhancement by active motions under DNP (SCREAM‐DNP), which often exploits fast reorientational dynamics—most notably of methyl groups—even at cryogenic temperatures. Beyond methyl groups, SCREAM‐DNP has also been demonstrated in molecular ring systems that retainactive conformational dynamics. Proline is a particularly relevant example, as its five–membered ring undergoes puckering dynamics that can persist under DNP conditions. While SCREAM‐DNP has been observed for free proline in frozen solution, the impact of incorporation within peptides on these dynamics remains poorly understood. Here, we present a systematic investigation of SCREAM‐DNP in proline and proline‐containing derivatives to elucidate how local structural context modulates the underlying dynamics. By comparing a series of dipeptides with proline at different sequence positions, we identify structural motifs that influence SCREAM‐DNP efficiency. These experimental results are further supported by energy barrier calculations, providing mechanistic insight into the conformational dynamics governing site‐specific DNP enhancement and guiding potential applications in structural biology.

## Graphical abstract

A systematic investigation of SCREAM‐DNP beyond methyl groups, using ring‐inversion dynamics on proline as a model compound. Comparison between free proline and proline‐containing oligopeptides at different sequence positions shows how local structural context modulates ring dynamics and site‐specific DNP enhancement. Energy barrier calculations provide mechanistic insight into the observed effects.

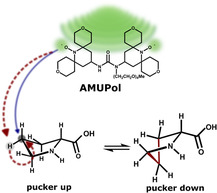

## 1 Introduction

Cross‐relaxation (CR) is a well–known mechanism within the field of NMR spectroscopy. It is triggered by mutual spin flips between two dipolar–coupled atoms undergoing relative motion [1]. During this process magnetization is exchanged between them, which can result in signal intensity transfer and is often utilized as the Nuclear Overhauser Effect (NOE) [2]. The cross–relaxation rate is dependent on the difference of the double and zero quantum transition probabilities and is thus dependent on the dipolar coupling strength and the timescale of the molecular motion, the latter of which is described by the molecular correlation time *τ*_c_ [3]. The transition probabilities decrease with the sixth power of the internuclear distance, setting a boundary for observing magnetization transfer by NOE at approximately 5 Å [3].

The same effect that is used daily in routine liquid state homonuclear NOESY experiments is also capable of triggering heteronuclear NOE, even if the sample is in a solid state [4]. Measurements in the solid state, even if successfully demonstrated in some cases [5, 6, 7, 8], present other problems. For the ^1^H homonuclear case, the main reasons are the lack of chemical shift resolution and the dominance of ^1^H–^1^H spin diffusion [9]. Heteronuclear experiments may still provide insights into different types of molecular motions [10]; however, under extreme conditions such as 100 K, where DNP‐enhanced MAS NMR is typically performed, only processes with a very low activation barrier can serve as a source of motion.

A prominent example is the rotational dynamics of a methyl group, which exhibits a low energy barrier for reorientation [11, 12, 13]. A striking effect of this motion in proteins was first observed in directly ^13^C DNP–enhanced MAS NMR, and described by Daube et al. [14]. Shortly thereafter, this effect has also been reported to occur in other systems such as polyethylene glycol chains [15], *N*‐functional groups [16], methylcellulose [17], including the active involvement of ring‐inversion (pseudorotation) motions demonstrated by Hoffmann et al. in cyclohexane [15], and Aladin et al. in proline [18].

In all cases, heteronuclear cross–relaxation manifests itself in DNP‐enhanced spectra recorded via a direct‐polarization readout (e.g., single‐pulse Bloch decay), while it is caused by an indirect polarization‐transfer pathway. Here, mobile proton spins are hyperpolarized via DNP and subsequently their polarization is spontaneously transferred to dipolar‐coupled heteronuclei, after which the hyperpolarization may spread through the heteronuclear network via spin diffusion. In parallel, the heteronuclei are also hyperpolarized directly via DNP. Experimentally, the NMR spectra based on magnetization transferred by each of the two pathways are selectively accessible via the pulse sequence and spectral post–processing according to Aladin et al. [14, 18, 19, 20, 21].

In recent years, there has been an interest in using DNP to achieve site specificity in NMR spectra, particularly in light of the severe spectral crowding often encountered in MAS NMR of large biomolecular complexes [19]. SCREAM‐DNP may provide one possibility of this sought‐after specificity especially since its potential was recently extended by combination with the MAS frequency‐selective recoupling method of rotational resonance (R^2^) [21]. This offers a high degree of sensitivity as well as both spatial and spectral selectivity and potentially provides access to distance information [21]. Next to methyl groups, another promising source for SCREAM‐DNP in proteins is proline due to a small energy barrier for its ring‐inversion or puckering motion, comparable with methyl groups, as indicated by quantum chemical calculations [11, 12, 13, 22, 23]. Therefore, proline presents an opportunity to expand polarization sources offering some new ways for analyzing biological systems by SCREAM‐DNP.

**FIGURE 1:**
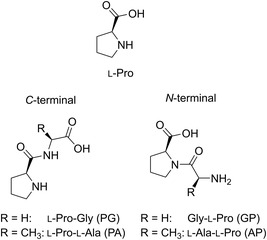
Overview of the chemical structures of investigated l‐proline derivatives.

Up to now, SCREAM‐DNP activity has only been investigated in detail on a frozen solution of free proline but not within peptides [18]. Thus, the question remains how the incorporation of proline into different peptide structures alters the underlying dynamics and subsequently the efficiency of SCREAM‐DNP. Therefore, we systematically investigated the influence of *C*‐ and *N*‐terminal modifications on proline ring dynamics in dipeptides, starting with the simplest possible modifications by adding glycine or l‐alanine in natural isotope abundance to each terminus of fully ^13^C,^15^N‐labeled proline. Adding alanine instead of glycine has also the advantage that another SCREAM‐DNP active group is present in the dipeptide so that the interplay may be studied. Thus, we present a systematic approach to analyze SCREAM‐DNP in proline and its derivatives with the aim of gaining a deeper insight into its dynamics under DNP conditions. We compare different dipeptides incorporating l‐proline at different positions (Figure 1) and discuss the temperature dependence of SCREAM‐DNP in order to determine which structures boost or quench the required dynamics. Since the *N*‐terminal peptide linkage involves the amino group situated in the five‐membered ring, we expect to observe a large impact on the cross‐relaxation propensity; in contrast, the impact by the peripheral *C*‐terminal modification should be marginal. The simplest amino acid to attach to both termini is glycine, which has also been shown to be impervious to SCREAM‐DNP and therefore should excludes any spurious side effects. Additionally, given that proteins commonly contain numerous (SCREAM‐DNP active) methyl‐bearing amino acids, we also link l‐alanine to l‐proline. This enables us to gain initial insights into the interplay and potential interference between several highly dynamic groups in close proximity.

## 2 Results and Discussion

### 2.1 DNP‐Enhanced Cross‐Polarization Experiments

**FIGURE 2:**
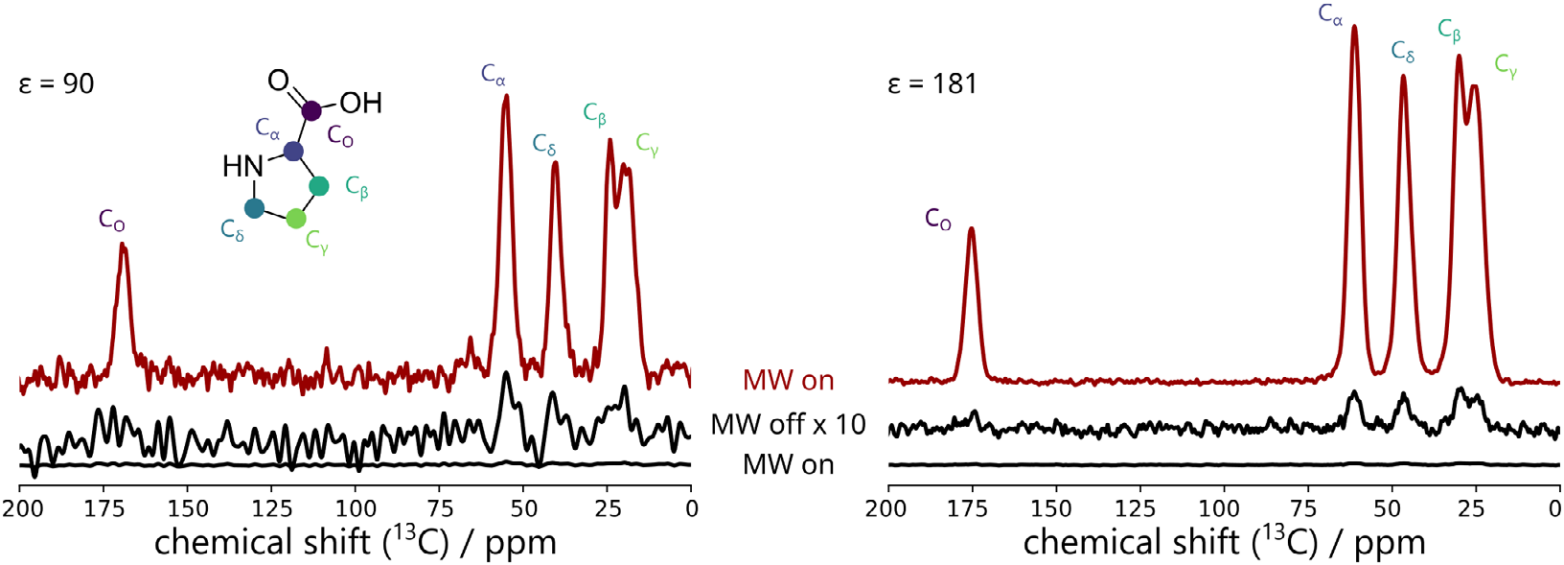
^1^H‐^13^C CPMAS spectra of 0.1 M ^13^C,^15^N‐proline (right) and 1 M natural abundance proline (left) with 10 mM AMUPol at 100 K, 18 kHz MAS and 3 s contact time. The experiment was performed with (µw on, red) and without (µw off, black) microwave irradiation. The total ^1^H DNP enhancement was determined as 181 for labeled and 90 for unlabeled proline.

DNP‐enhanced ^1^H‐^13^C cross‐polarization magic‐angle spinning (CPMAS) experiments at 100 K and 18 kHz spinning frequency (Figure 2) show the expected signals arising from 0.1 M fully ^13^C,^15^N‐labeled proline in ^12^C_3_‐glycerol‐d_8_/D_2_O/H_2_O (60/30/10 vol.‐%) as well as 1 M proline in natural isotope abundance using 10 mM AMUPol as polarizing agent. Resonances could be found at 175 ppm for the carbonyl, 61 ppm for C_α_, 46 ppm for C_δ_, 29 ppm for C_β_, and 24 ppm for C_γ_. The ^1^H DNP enhancement was found to be approximately 181 for labeled proline and 90 for natural abundance proline, based on amplitude comparison with the respective CPMAS spectrum in the absence of microwave irradiation; the observed enhancement factors were uniform for all signals in each sample. The observed differences in enhancement are most likely attributable to variations in the concentration of labeled (0.1 M) versus natural abundance proline (1 M). In the natural abundance sample, the proline concentration is approximately tenfold higher resulting in a greater number of molecules that accelerate *T*_1_ relaxation which results in lower enhancement. Measurements of each sample at higher temperatures reveal no noteworthy difference in chemical shifts but, as expected, a reduction of DNP enhancement with rising temperature. At 125 K an enhancement factor of approximately 128 could be found for labeled proline, which further decreased to approximately 107 at 150 K. Similar experiments were performed on all other proline derivatives (GP, PG, AP, and PA) where only the proline residue was isotope labeled and minimal shifts in resonance frequency are found, with the same temperature dependence with virtually equal ^1^H enhancement factors in each case. An overview about all determined enhancement factors for all samples and all temperatures can be found in Table S3.1.1.

### 2.2 Proline Cross Relaxation Dynamics at 150 K

Next, we performed SCREAM‐DNP experiments using the pulse sequence and processing described before, aiming first to reproduce the pronounced SCREAM‐DNP activity of proline demonstrated in 2019 by Aladin et al. [18]. However, due to technical circumstances, here, we conducted the experiments at a MAS frequency of 18 kHz instead of 8 kHz.

The pulse sequence consists of two acquisition blocks. To provide better reproducibility, in both blocks both the ^1^H and ^13^C spins’ magnetizations were fully quenched prior to the build‐up period by a 90°‐pulse train. Spectra were then recorded by single‐pulse excitation (Bloch decay) after various delay periods (*t*_pol_). In the first block, no ^1^H pulses were applied during this delay period and, thus, a typical direct polarization (DP) spectrum is obtained, containing both direct (i.e., electron to ^13^C) and indirect DNP (i.e., electron to ^1^H and then to ^13^C via CR) transfer pathways. However, during the second block, all ^1^H spin magnetization is continually inverted during the polarization buildup period by 180° pulses, resulting in a DP_sat_ spectrum that ideally only contains contributions from the direct DNP transfer pathway. Numerical subtraction yields the ΔDP_sat_ spectrum, which shows only the magnetization transferred via the CR‐induced indirect pathway. Note that in the ΔDP_sat_ spectrum, all signals typically have negative intensity as an intrinsic result of the CR mechanism, but they are presented below after mathematical inversion (i.e., 180° phase shift) for simplicity. The resulting buildup curves were fitted either exponentially or biexponentially. The buildup efficiency is therefore described by one or two buildup time constants—in the biexponential case both a fast (*t*_f_) and a slow (*t*_s_) constant is obtained; in the monoexponential case we refer to the single time constant as *t*_f_ for easier comparability.

**FIGURE 3:**
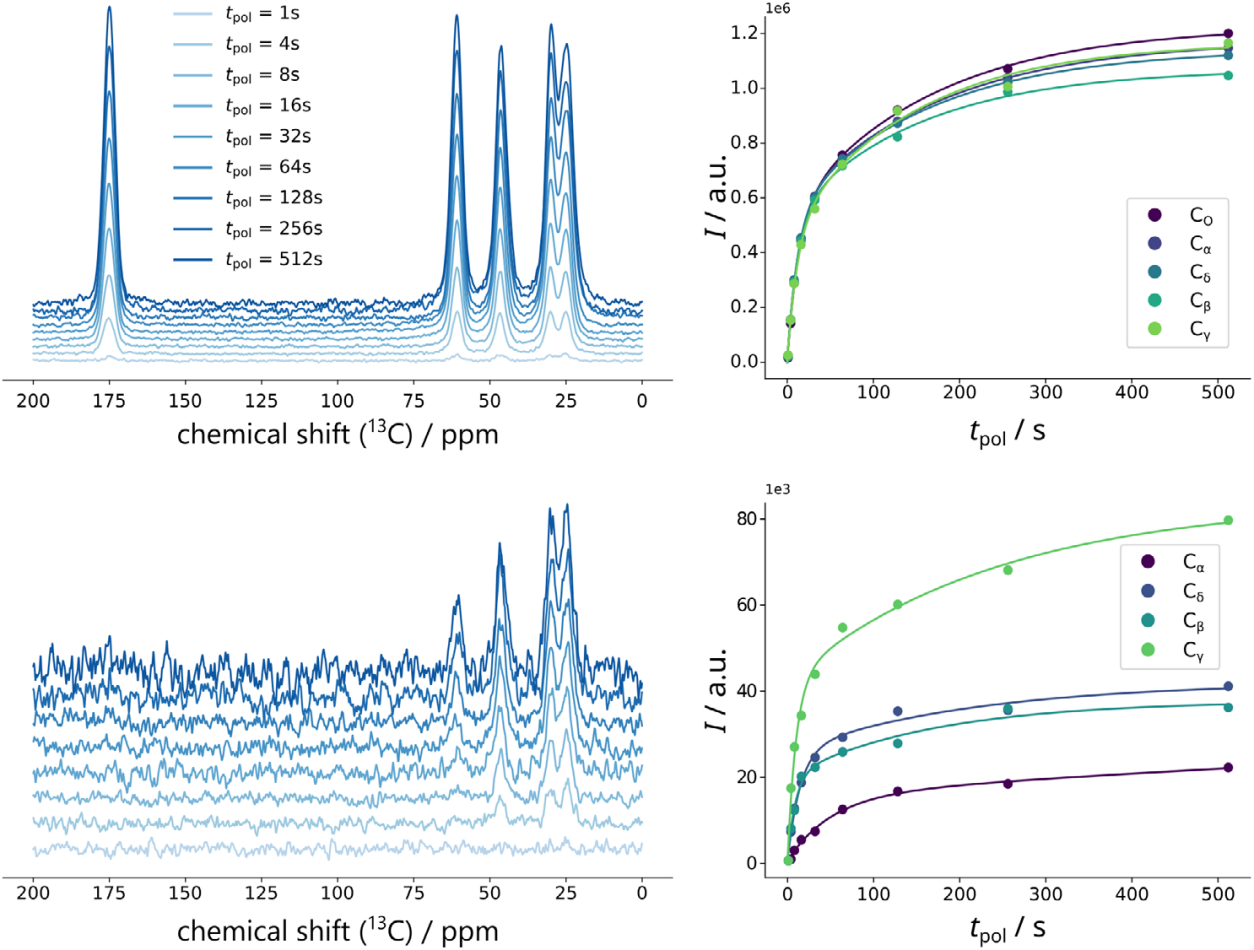
Left: Comparison of DNP‐enhanced ^13^C MAS NMR ΔDP_sat_ spectra of 0.1 M uniformly ^13^C,^15^N‐labeled (top) and 1 M natural abundance (bottom) l‐proline at 150 K, with polarization times ranging from 1 s to 512 s at 18 kHz MAS. Right: Polarization build‐up dynamics for each carbon position in uniformly labeled (top) and natural abundance (bottom) l‐proline. Solid lines represent biexponential fits—except for C_α_ of natural abundance proline, which was fitted to a single‐exponential function—with their parameters provided in Tables S4.3 and S4.15.

For ^13^C,^15^N‐labeled samples at 150 K and an MAS frequency of 18 kHz, the buildup time constants were determined to be approximately 12 s for *t*_f_ and 150 s for *t*_s_ (See Table S3.2.3). These values differ only slightly from previously reported measurements, where *t*_f_ was approximately 10 s and *t*_s_ was approximately 100 s. This can be attributed to the higher MAS frequency, which enhances dipolar decoupling and reduces both spin diffusion and cross‐relaxation efficiency. For ^13^C‐labeled samples, it is reasonable to consider only the mean intensity across all resonances, as intramolecular spin diffusion within the ^13^C system quickly distributes the enhanced polarization, leading to a uniform signal buildup (See Figure 3). Therefore, unless otherwise noted, we only report a single value for uniformly isotope‐labeled samples.

For proline in natural isotope abundance, the probability to find more than one ^13^C on each molecule is negligible, and thus individual build‐up time constants are obtained for each signal. For C_γ_, C_β_, and C_δ_, rapid buildup with time constants of approximately 10 s were observed, whereas for C_α_, a value of around 60 s was determined. While this confirms earlier reports, in this work the effect on the carbonyl group could not be observed here due to the approximately 12‐fold reduced sample volume in the 1.3 mm rotors as compared to 3.2 mm rotors used in the earlier study [18]. The differences in overall enhancement can arise from multiple factors. On the one hand, the signal intensity correlates directly with the number of attached protons—two for C_γ_, C_β_, C_δ_; one for C_α_; and none for the carbonyl carbon (C_O_). On the other hand, variations in the amplitude of ring inversion dynamics across different parts of the proline ring are also expected [18].

### 2.3 Temperature Dependence SCREAM‐DNP Activity in ^13^C‐Labeled Proline

To further investigate the dynamics of the proline ring, we analyzed the above‐discussed behavior at three different temperatures. A comparison of buildup curves for proline at 100, 125, and 150 K reveals that the maximum achievable SCREAM‐DNP signal intensity—defined as the plateau value *A*_f_ in the case of exponential buildup or as the sum of the plateau values *A*_f_ and *A*_s_ in the case of biexponential buildup—is only slightly temperature‐dependent, showing a decrease at higher temperatures.

**FIGURE 4:**
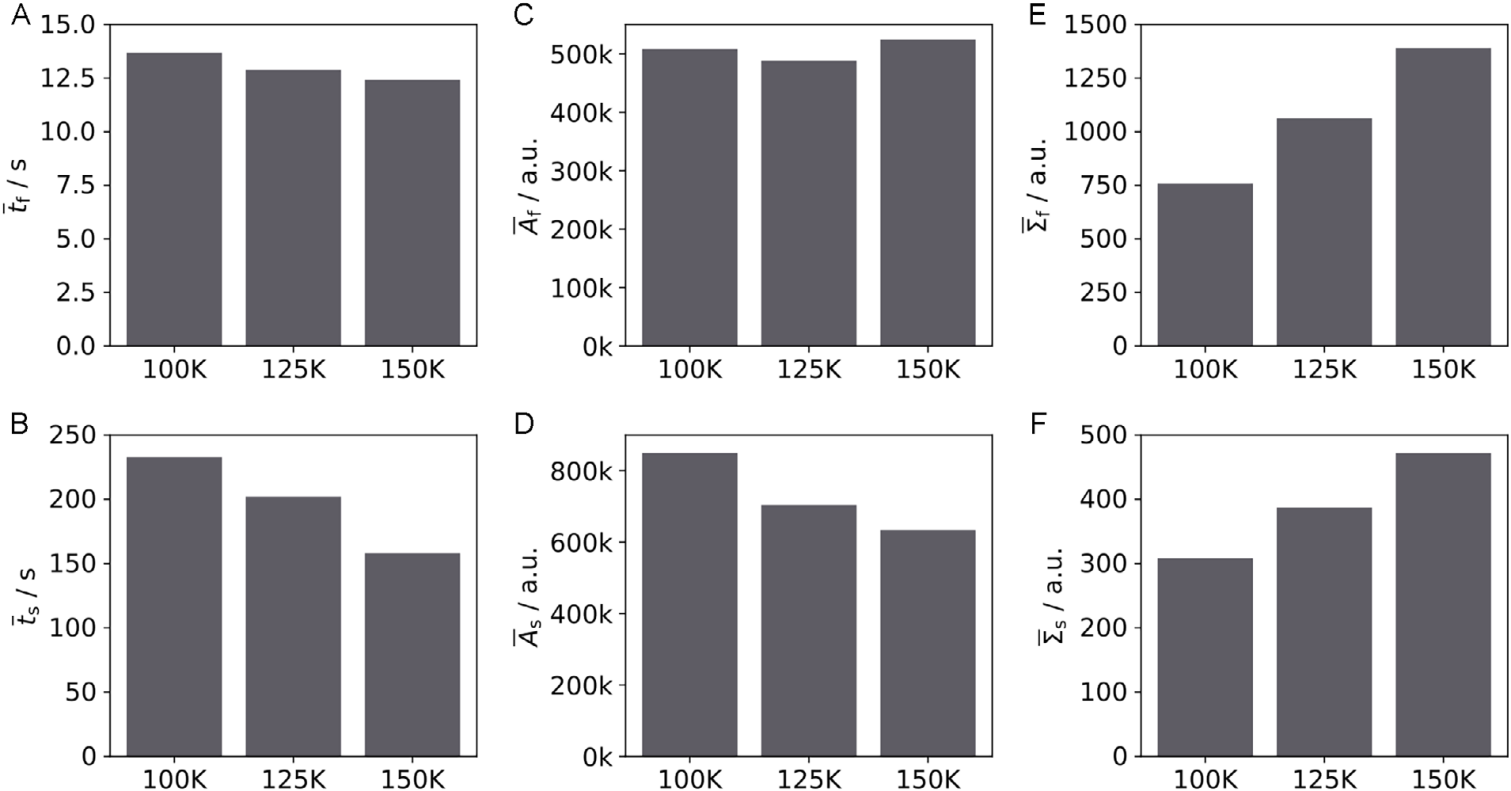
Bar plots showing the mean values of various parameters extracted and calculated from buildup analysis of l‐proline (0.1 M) at different temperatures (100, 125, and 150 K) and 18 kHz MAS frequency. (A) Mean *t*_f_ values (in seconds). (B) Mean *t*_s_ values (in seconds). (C) Mean *A*_f_ values (in arbitrary units, a.u.). (D) Mean *A*_s_ values (in a.u.). (E) Mean *Σ*_f_ values (in a.u.). (F) Mean *Σ*_s_ values (in a.u.).

A closer examination of both components contributing to the total signal intensity reveals that the fast component is nearly temperature‐independent, whereas the plateau amplitude of the slow component slightly decreases at higher temperatures (Figure 4C,D). A similar trend is observed for the buildup time constants: the fast component builds up with a constant of approximately 13 s at 100 K and approximately 12 s at 150 K, while the slow component shows a significantly greater temperature dependence, with a difference of 75 s between 100 and 150 K (Figure 4A,B).

Analyzing the resonance‐specific *t*_f_ values instead of their mean reveals that polarization indeed seems to build up slightly more rapidly on C_γ_, followed by the other ring carbons, while the slowest buildup time can be assigned to the carbonyl group. This is supported by the above‐discussed observation from natural abundance proline that C_γ_ is the most mobile group within the system. Nevertheless, the differences in buildup time constants are on the order of 1 s, which is comparable to the fitting error, indicating that ^13^C spin diffusion effectively dominates the distribution of magnetization.

Since the fast buildup time constant slightly but significantly increases at higher temperatures, we can infer a marginally higher ring mobility. To further quantify the efficiency of SCREAM‐DNP activity in proline, we calculated the ^1^H enhancement‐normalized sensitivity for the fast and slow processes according to

$${\text{Σ}}_{i}=\frac{A_{i}}{\varepsilon \cdot \sqrt{t_{i}}}$$

where i represents either the slow or fast buildup component. In order to act as a measure for cross‐relaxation efficiency, this approach attempts to compensate for the decreasing ^1^H enhancement with increasing temperature; as a consequence of the higher ring mobility, this enhancement‐normalized sensitivity increases at higher temperatures (Figure 4E,F).

### 2.4 *C*‐Terminally Modified Proline

SCREAM‐DNP buildup profiles were measured and analyzed at 100 and 150 K for all four dipeptide permutations prolylglycine (PG) and prolylalanine (PA) as well as glycylproline (GP) and alanylproline (PA). For both *C*‐terminally modified samples, the fast buildup time constants are around 14 s at 100 K and the slow constants around 220 s. At 150 K, the buildup is only slightly faster with time constants of around 13 and 200 s, respectively. As a result of the congruence of the buildup behavior of the unmodified proline and both dipeptides, we conclude that the puckering motion of the proline ring does not appear to be significantly affected by any *C*‐terminal peptide modification. Also, the methyl group seems to be sufficiently far removed from the ^13^C_5_ system of proline that its motion does not significantly shorten the buildup time constants.

**FIGURE 5:**
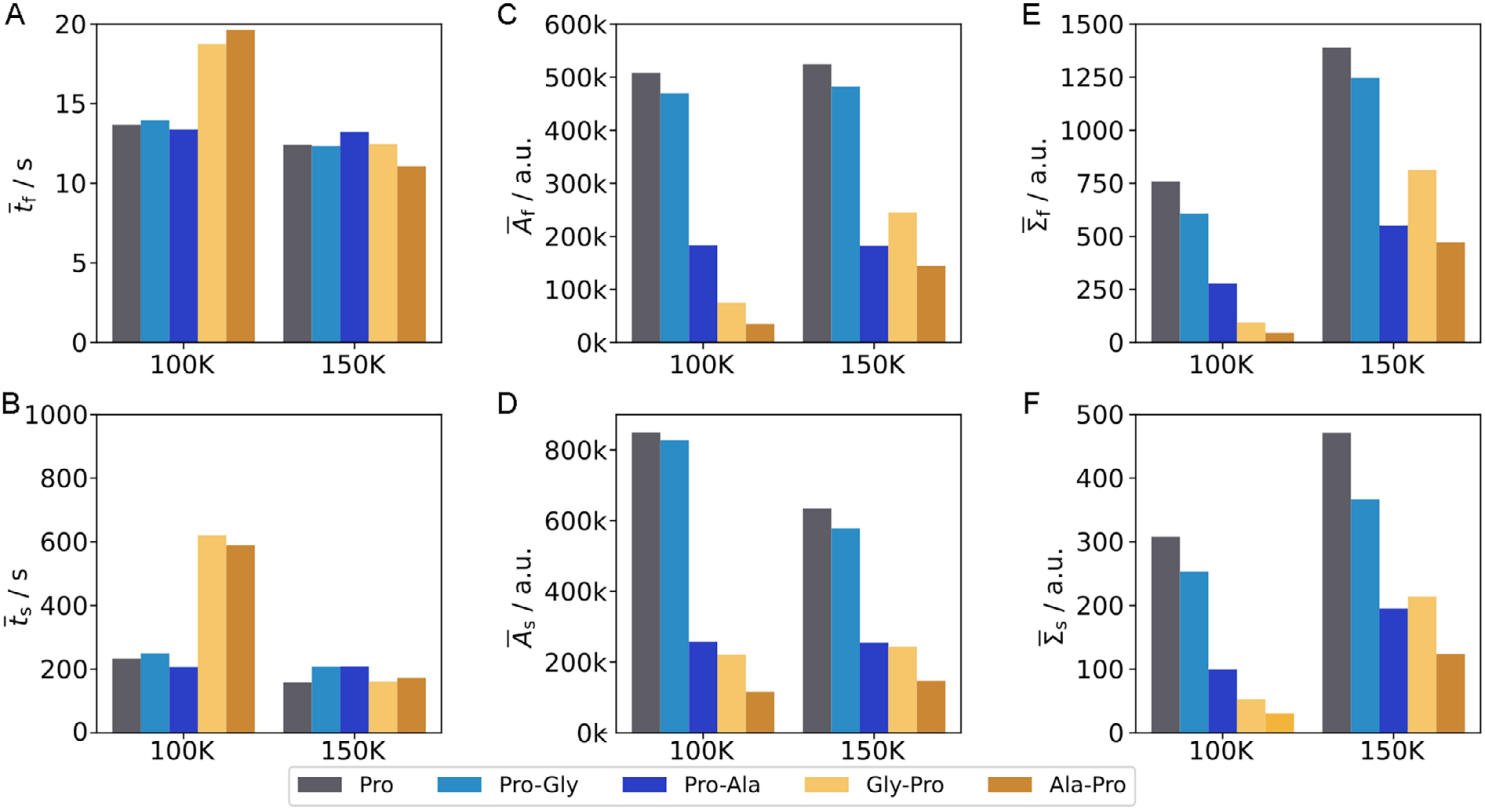
Temperature dependence of relaxation parameters for different proline samples. (A) Mean *t*_f_ values at 100 and 150 K for five different samples: Pro (gray), Pro‐Gly (light blue), Pro‐Ala (dark blue), Gly‐Pro (light yellow), and Ala‐Pro (dark yellow). (B) Mean *t*_s_ values for the same conditions and samples. (C,D) Mean *A*_f_ and *A*_s_ values for the same conditions and samples. (E, F) Mean *Σ* values, for the same samples at both temperatures.

In contrast to the buildup time constants, the ^1^H enhancement factor as well as the ^1^H‐^13^C CR‐efficiency, apparent as the enhancement‐normalized sensitivity (see Figure 5) reveals that the modification does indeed have an influence on these parameters, though. Comparing both *C*‐terminally modified prolines and the free proline reveals a similar trend, where the effectiveness of heteronuclear NOE increases at higher temperatures while the maximum achievable enhancement values decrease. Notably, PA exhibits a significant reduction in these values, with enhancement and sensitivity values dropping to approximately one half to a quarter of those seen in free proline or PG, therefore, we attribute this to be caused by the methyl group reorientation.

### 2.5 *N*‐Terminally Modified Proline

**FIGURE 6:**
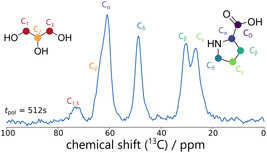
DNP enhanced ^13^C MAS NMR ΔDP_sat_ spectra for Gly‐^13^C,^15^N‐l‐proline (0.1 M) with 512 s polarization time showing the appearance of glycerol resonances at 72 ppm (C_2_) and 63 ppm (C_1,3_).

The *N*‐terminal modification of proline is expected to have a larger impact on the ring dynamics than the *C*‐terminal variants because the nitrogen in proline's pyrrolidone ring is directly involved in the peptide bond. We expect that due to the *π*‐orbitals involved in an amide bond and its participation in conjugation, a stiffening of the whole ring may occur. For both *N*‐terminal modified samples, it is noteworthy that the DNP matrix was not prepared with ^13^C‐depleted glycerol. Besides the occurrence of two additional signals from glycerol at long SCREAM‐DNP polarizing times, we expect no influence on the DNP buildup dynamics. Unfortunately, however, one of them overlaps with the C_α_ signal (see Figure 6). Since spectral deconvolution is difficult in this case, the buildup curve of this overlapped signal is distorted. Therefore, for these samples, the mean values were calculated excluding C_α_ but including all other resonances. Any deviation arising from this should be minimal since the glycerol signals appear only when spectra were recorded with long polarization times; indeed, the influence on the fast buildup time process is nearly negligible, even if C_α_ was included (see supporting information Tables S4.8–S4.11).

Comparing SCREAM‐DNP buildup dynamics between Pro, GP, and AP shows that potential differences in ring puckering dynamics, if existing, were not significantly influencing the buildup time constants at 150 K. On average, a fast buildup time constant of approximately 12 s besides a slow component of around 150 s were observed for all three samples at this temperature (see Figure 5). However, the overall signal intensity—and with it the contribution to the overall sensitivity from both the fast and slowly building up components—is reduced by about half, and even more for AP, in comparison to proline or PG. This observation indicates that a smaller amount of polarization is transferred, while the rate constant remains unchanged. The most plausible explanation for the additional signal reduction of AP in comparison to GP is the previously mentioned influence of the methyl group. This effect can also be observed for PA in comparison to PG and impacts the ^1^H longitudinal relaxation time (*T*_1_). As a result, it leads to an overall reduction of the enhancement factor across the sample (see Supporting Information, Table S3.1.1). In particular, the maximum achievable ^1^H polarization in the immediate vicinity of the dipeptides is likely reduced in a disproportionate manner. This reduction then accounts for the observed decrease in signal intensity.

When cooling to 100 K, a noteworthy effect appears in all parameters discussed earlier. The fast buildup time increases by approximately 5–6 s, while the slow component increases by about 400 seconds for both *N*‐terminal‐modified proline residues. Moreover, the previously distinctly biexponential buildup transitions more toward a stretched exponential behavior.

A significant change is also observed in the SCREAM‐DNP enhancement. While *A*_s_ shows almost the same intensity at 100 K as at 150 K—unlike free proline and *C*‐terminal modified proline*—A*_f_ is nearly absent, or at least much less pronounced, indicating that SCREAM‐DNP is essentially weaker at this lower temperature. Upon heating to 150 K, *A*_f_ increases by a factor of about four. Overall, this suggests that *N*‐terminally modified proline exhibits stronger thermally activated behavior. Both components’ contributions to the overall sensitivity again just follow the trend discussed for their respective amplitudes.

### 2.6 Calculations of Ring‐Inversion Energy Barrier

**FIGURE 7:**
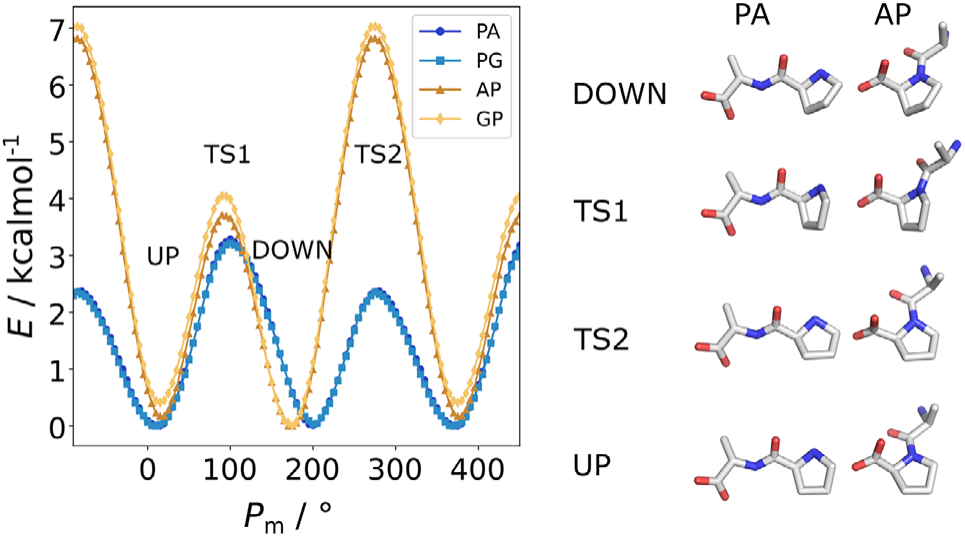
Left: Energy profiles for proline ring inversion calculated for PG, PA, GP, and AP, showing the relative energies of the up and down conformations and the corresponding transition states. *P*_m_ is the pseudorotation phase angle. Right: 3D‐ structures of of AP and PA in the up and down conformations as well as in the transition states (TS1 and TS2). Carbon: gray, oxygen: red, nitrogen: blue.

The cross‐relaxation efficiency depends strongly on the ring‐puckering dynamics which in turn are dictated by the ring‐inversion energy barrier. Thus, we calculated its energy profile for all four dipeptides (see Figure 7) to further support the findings of the SCREAM‐DNP experiments. The ring inversion can be described via the phase angle of pseudorotation of the five‐membered ring. The phase angle here is determined by a combination of all five *χ* angles involved in the ring‐puckering movement (For further details see Supporting Information S6).

The up and down (or *exo* and *endo*) conformations of proline are found at the characteristic minima of the energy profile. As can be seen, the energy profiles for each pair of *C*‐ or *N*‐terminally modified proline‐containing dipeptides are almost identical, which is consistent with the observations of both the fast and the slow buildup times. However, for both *N*‐terminally modified compounds, the activation energy for the ring flip is noticeably higher than it is for the *C*‐terminally modified peptides. This is in agreement with the results from the SCREAM‐DNP measurements manifesting in higher buildup time constants for AP and GP.

Comparing the ring‐inversion transitions in more detail, in both PA and PG, the activation barrier is around 2.3 kcal mol^−1^ via transition state 2 (TS2) while the path along transition state 1 (TS1) is only slightly higher at 3.2 kcal mol^−1^. In AP and GP, however, TS1 provides the lower energy path at about 3.8 kcal mol^−1^, while the path via TS2 requires as much as 7 kcal mol^−1^. Interestingly, the transition‐state structures for AP and PA are almost identical (the same holds for GP and PG, though we focus the discussion here on AP and PA). This indicates that in both systems the ring must traverse essentially the same geometrical landscape during the switch between *exo* and *endo* conformations. However, the nitrogen's sp^2^ character is greater in AP than in PA, which makes it more difficult for AP to pass through this state. The sum off all three C–N bond angles with proline nitrogen as the central atom can confirm this. For GP and AP, the sum is approximately 360° in all four conformations (up, down, TS1, and TS2), indicating almost perfect planarity. For PG and PA, as expected for sp^3^ hybridized molecules, the sum of bond angles is lower (333° for the *endo* state, 326° for the *exo* state, 322° for TS1, and 324° for TS2, as compared to 321° in NH_3_ or 328° in NH_4_^+^).

A key unresolved issue concerns the exact nature of the proline motion that is relevant in SCREAM‐DNP experiments. In principle, two mechanistic scenarios representative of extreme cases can be considered. The first involves a complete inversion of the ring, whereas the second corresponds only to localized vibrational motions in the two observed energy minima. Support for the latter hypothesis arises from the substantial spatial rearrangement required for a full ring inversion. Such a process would likely necessitate displacement of surrounding solvent molecules to accommodate the transient geometry, which may be kinetically hindered at temperatures near 100 K. Both processes could also occur concurrently but with different probabilities. In this framework, the presence of two distinct buildup time constants could be rationalized by assigning the faster component to vibrational motion between minima and the slower component to the full ring inversion. However, to prove this, the exact origin of the slow buildup time constant must be evaluated. An alternative explanation of the occurrence of the slow component may be intermolecular transfer between two proline moieties. This should be addressed in the future by a proline concentration‐dependent analysis. If the slow buildup component is dependent on proline–proline interactions, a reduction in concentration should lead to a less pronounced slow buildup component, since the mean dipolar coupling would be weaker and, with this, the transition probability of heteronuclear NOE lower. A first indication that the effect is not due to a proline–proline interaction can already be obtained by comparing proline samples with and without carbon‐13 labeling (see Figure 3). For example, the solution of ^13^C_5_‐proline at a concentration of 0.1 M, with 100 % ^13^C enrichment, has a ^13^C concentration at the C_γ_ position of 0.1 M. In contrast, the unlabeled proline solution at 1 M, with the natural abundance of ^13^C (about 1 %), has a ^13^C concentration at the C_γ_ position of only 0.01 M—a ten‐fold lower value. Despite this large difference in ^13^C concentration, a biexponential buildup behavior is still observed (see Figure 3) in both cases. This suggests that the slow buildup component is most likely not caused by intermolecular interactions between proline molecules.

### 2.7 ^15^N SCREAM‐DNP Experiments

**FIGURE 8:**
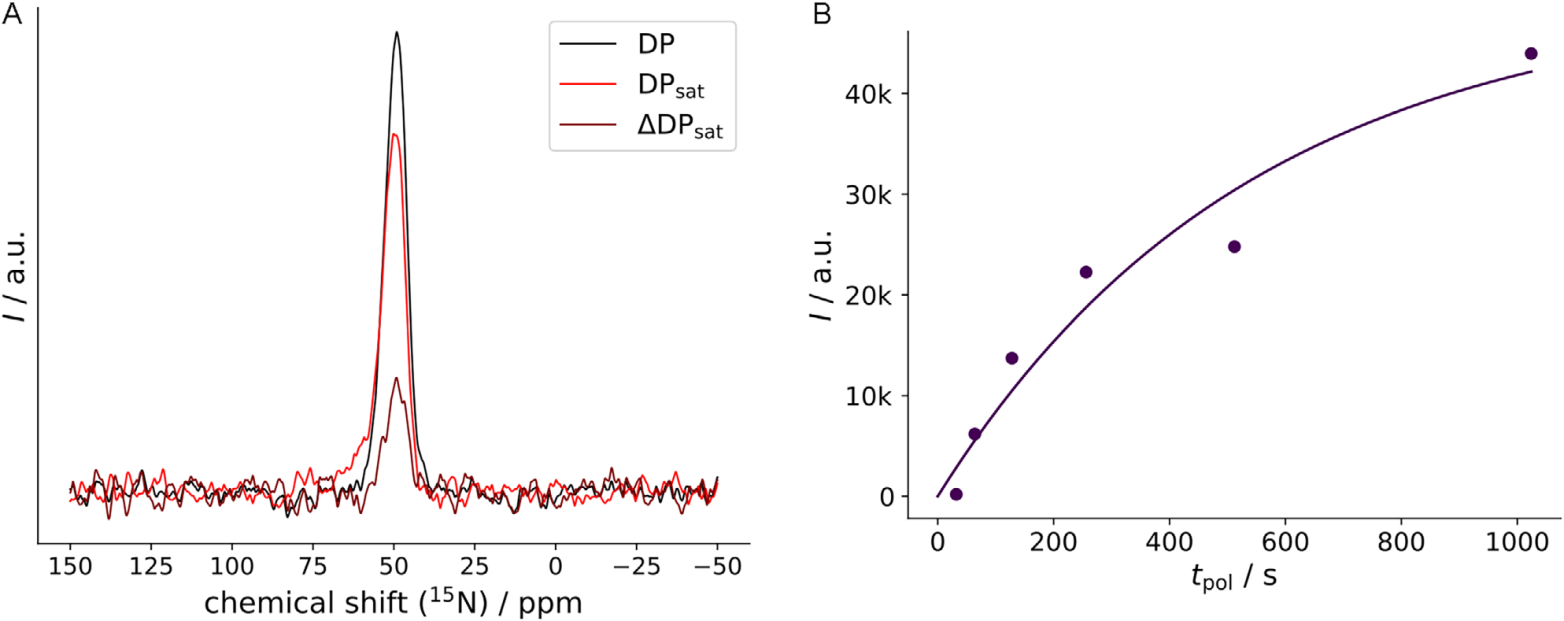
A: ^15^N‐DP (black), ^15^N‐DP_sat_ (blue) and ^15^N‐ΔDP_sat_ (red) spectra of ^13^C_5_,^15^N‐l‐Pro at 100 K, *ω*_MAS_ = 18 kHz, *t*_pol_ = 1024 s. ΔDP_sat_ is positive in this case due to the negative gyromagnetic ratio of ^15^N. B: Polarization build‐up dynamics for the ^15^N atom in l‐proline. Solid lines represent exponential fits with their parameters provided in Table S4.5.

Given the distinct ^1^H‐^13^C SCREAM‐DNP activity exhibited by in the ^13^C,^15^N‐labeled proline, we expected that ^15^N could also be subject to CR. To test this hypothesis, a SCREAM‐DNP buildup series with polarizing periods between 32 s and 1024s was measured at a temperature of 100 K. As depicted in Figure 8A, the CR efficiency of ^15^N is much lower when compared to carbon atoms. Note that the ΔDP_sat_ spectrum is positive in this case due to the negative gyromagnetic ratio of ^15^N. The low level of SCREAM‐DNP activity can be attributed, besides the 2.5‐times smaller gyromagnetic ratio of ^15^N in comparison to ^13^C and consequentially smaller dipolar interactions, to the larger distance between the ^15^N nucleus and the protons involved in the puckering motion. The nitrogen itself is not directly involved in the motion within the proline ring. As a result, the indirect CR pathway in this scenario is mediated by the ^1^H‐^15^N dipolar coupling to the H_δ_ and H_γ_ protons, which are approximately 2–4 Å away; thus, the *r* ^*−*6^ scaling of CR yields only a small ΔDP_sat_ signal. A buildup time constant of 521 s could be estimated via a monoexponential fit of this series (Figure 8B); however, the low number of points and rather poor signal‐to‐noise ratio did not allow for an unambiguous distinction between mono‐ or biexponential buildup behavior.

## 3 Experimental

### 3.1 Synthesis

For the synthesis of the *C*‐terminal modified proline derivatives, solid phase peptide synthesis was used (see Supporting Information Section S2.2). The *N*‐terminal modifications were synthesized using standard peptide synthesis procedures in solution (see Supporting Information S2.3). Note that in all cases, the l‐amino acids were used.

### 3.2 DNP Sample Preparation

For DNP measurements, two stock solutions were prepared. One containing 20 mM AMUPol and one containing 200 mM of ^13^C,^15^N labeled proline or derivative in 60 vol‐% ^12^C_3_‐glycerol‐d_8_, 30 vol‐% D_2_O, and 10 vol‐% H_2_O (DNP matrix). For samples containing Gly‐Pro or Ala‐Pro dipeptides, glycerol‐d_8_ was used instead of the ^13^C depleted one. Afterwards, both stock solutions were mixed in a ratio of 1:1 to get a final concentration of 10 mM AMUPol and 100 mM proline or derivative. These aliquots were transferred to a 1.3 mm zirconia rotor with a Vespel drive cap. For samples of natural abundance, a 2 M stock solution of the corresponding species was prepared and then mixed in a 1:1 ratio with the 20 mM AMUPol stock solution, resulting in a final concentration of 1 M.

### 3.3 DNP‐Enhanced MAS‐NMR Experiments

The experiments were carried out on a Bruker AVANCE III HD spectrometer with an operating field of 400.5 MHz ^1^H frequency with a Bruker ASCEND DNP 9.4 T widebore (89 mm) magnet. 263.4 GHz microwaves were produced by a Bruker/CPI second‐harmonic gyrotron operating at a field of 4.8 T with a 132 mA beam current. All experiments were performed using rf powers of 170 kHz (^1^H), 60 kHz (^13^C), and 50 kHz (^15^N) for 1.3 mm probe operating in ^1^H/^13^C/^15^N triple channel mode. The temperature was detected via a thermocouple inside the MAS stator. ^1^H, ^13^C, and ^15^N 90°‐pulse power was determined in Hartmann‐Hahn CP experiments with 2.00 ms contact time. SPINAL‐64 was used for decoupling of ^1^H during acquisition. MAS with a spinning frequency of 18 kHz was used for all experiments to exclude the effects of rotational resonance.

### 3.4 Determination of Enhancement Factor

The ^1^H enhancement factor (*ε*_DNP_ = *I*_on_/*I*_off_) was measured using standard CP experiments with and without active microwave under the conditions mentioned above. An overview about all determined enhancement factors is given in Supporting Information Table S3.1.

### 3.5 Recording of SCREAM‐DNP Spectra

For SCREAM‐DNP experiments, a series of 16 90° pulses, each separated by 5 ms, were applied to both ^1^H and ^13^C/^15^N nuclei. This pulse train aimed to eliminate all magnetization, before allowing the polarization to build up during a variable polarization time (*t*_pol_ = 1, 4, 8, 16, 32, 64, 128, 256, 512 s) to generate the DP spectrum. To obtain the DP_sat_ spectra, the same experiment was carried out with additional 180° ^1^H pulses every 250 ms to prevent ^1^H polarization buildup during the ^13^C polarization time. The number of ^1^H pulses varies depending on the polarization buildup time. The ΔDP spectra were obtained by mathematically subtraction of the DP_sat_ from the DP spectrum (ΔDP_sat_ = DP – DP_sat_).

### 3.6 SCREAM‐DNP Data Evaluation

SCREAM‐DNP data evaluation was performed using the screamlab (v0.1.0) [24] Python package for python (v3.10.7) [25]. Data evaluation was performed in two successive steps. In the first step, each carbon resonance in each ΔDP_sat_ spectrum was fitted to a Voigt profile. The line broadening parameters as well as the resonance position (µ) were fitted globally over all polarization times *t*_pol_. The Gaussian (*σ*) and Lorentzian (*γ*) line broadening parameter were thereby allowed to vary between 0 and 3 ppm, the amplitude (*A*) was restricted to non‐negative values, and the peak center (µ) was allowed to vary within ±1 ppm around a given value. A prefit was performed on the spectrum corresponding to the longest polarization time. Complete fitting results, including numerical parameters and graphical representations, are given in Supporting Information Section S3.2. In a second step, the time evolution of the integrated peak areas was fitted to a biexponential buildup model

$$I\left(t_{\mathrm{pol}}\right)=A_{f}\left(1-\exp\left(-\frac{t_{\mathrm{pol}}}{t_{f}}\right)\right)+A_{s}\left(1-\exp\left(-\frac{t_{\mathrm{pol}}}{t_{s}}\right)\right)$$

where *I*(*t*_pol_) denotes the signal integral at polarization time *t*_pol_, *A*_f_ and *A*_s_ are the plateau integrals of the fast and slowly building‐up component, and *t*_f_ and *t*_s_ are their respective buildup times. Complete fitting parameters for the second step are given in Supporting Information Section S4 together with corresponding graphical representations. Subsequently, for uniformly ^13^C,^15^N‐labeled samples, the mean values of the parameters *A*_f_, *A*_s_, *t*_f_, and *t*_s_ were determined by averaging across all five proline carbon resonances. In the case of the AP and GP samples, only four resonances were included in the calculation due to spectral overlap of the glycerol signal with the C_α_ resonance.

## 4 Conclusion

We demonstrated that the spin dynamics induced by the ring puckering motion of proline and all tested dipeptides are strongly pronounced even at low temperatures between 100 and 150 K, yielding heteronuclear NOE effects between ^1^H and ^13^C or even ^15^N. However, the rates at which this effect manifests in the SCREAM‐DNP buildup are slower than those shown in various cases relying on methyl group reorientation and therefore these molecular dynamics are not equally effective. For unmodified proline, it could be shown that the ring inversion dynamic and with this the SCREAM‐DNP buildup is fastest at 150 K and also that the obtained signal per unit time is highest. This trend could be found for all investigated species. In general, the mobility seems not to be significantly influenced by peptide bonding to the *C*‐terminus. Attaching glycine or alanine shows almost no effect on the buildup time constants but the methyl group of alanine yields a drastic decrease of the enhancement‐normalized CR efficiency as it serves as relaxation sink. Further insight into the actual mechanism of interference could be obtained by investigation of the distance dependence of this effect, e.g., by using a spacer between proline and alanine. In contrast to the *C*‐terminal modification, a significant reduction in ring mobility could be found by attaching glycine or alanine to the *N*‐terminus. At lowest temperature (100 K), a much slower polarization transfer via the indirect pathway could be observed. Through increasing of temperature the ring dynamics could be again increased until it reaches the same level as in the free amino acid at 150 K. The addition of another methyl‐bearing amino acid to the *N*‐terminus has around the same effect as observed for the *C*‐terminus. Therefore, in a biomolecular context, it is advisable to control for SCREAM‐DNP active amino acids in the direct surrounding of the proline of interest.

Examining the practical sensitivity across all samples and temperatures, it is evident that the highest values consistently occur at 150 K, even though the DNP‐enhanced ^1^H polarization being transferred by SCREAM‐DNP is smaller than at 100 K. In certain cases, measurements at lower temperatures may still be advantageous to facilitate the differentiation between signals influenced by proline ring puckering and those enhanced by processes such as methyl group rotation.

Finally, it was shown that it is possible to transfer polarization from ^1^H to ^15^N within proline via a ^1^H‐^15^N CR, even if the transfer is slow, most likely due to the rather small dipolar couplings involved. Beyond the influence of ring puckering on proline itself, polarization transfer to the solvent becomes evident at long polarization times. However, as mentioned before, more investigations on a potential SCREAM‐DNP activity of glycerol are needed. An important question arises regarding the effects of incorporating larger and more structurally complex amino acids than the small and simple glycine and alanine utilized in this study. Specifically, it would be of interest to modify the system with sterically demanding residues such as tryptophan. Since dipeptides remain relatively small compared to most naturally occurring peptides or proteins, an increase in system size including the propensity of proline‐containing peptide chains for forming secondary structure elements such as α‐helices is of significant interest in future investigations.

## Funding

This study was supported by the Deutsche Forschungsgemeinschaft (Grant TRR‐386 (Project 514664767) and INST 264/177‐1 FUGG).

## Conflicts of Interest

The authors declare no conflicts of interest.

## Supporting information

Supporting Information containing detailed description of chemical synthesis, details on spectral and buildup‐curve fitting, information on energy barrier calculation, and additional references is available.

## Data Availability

The data that support the findings of this study are available from the corresponding author upon reasonable request.
