# Supplementary material for: Proline Ring‐Inversion Dynamics in Peptides Enable Cross‐Relaxation‐Based Dynamic Nuclear Polarization
Source: Chemphyschem. 2026 Jun 14;27(11):e70444. doi: 10.1002/cphc.70444 (PMC13265247; doi:10.1002/cphc.70444)
Supplement: Supplementary file 1 — Supporting Information containing detailed description of chemical synthesis, details on spectral and buildup‐curve fitting, information on energy barrier calculation, and additional references is available. [file CPHC-27-e70444-s001.pdf]

SUPPORTING INFORMATION

Proline ring-inversion dynamics enable cross-relaxation-based dynamic nuclear polarization

*Florian Taube, Max Gierth, Georg Künze and Björn Corzilius**

Contents:

S1. Experimental

S2. Syntheses of compounds

S3. NMR data analysis

S4. Buildup analysis

S5. Calculation of energy barriers

S6. References

S1. Experimental

S1.1. Liquid state NMR

Liquid NMR spectra were recorded on Bruker spectrometer (AVANCE 500) and were referenced internally to the deuterated solvent (^{1}H: $(CD_3)_2SO$ δ_{ref} = 2.50 ppm; ^{13}C: $(CD_3)_2SO$ δ_{ref} = 39.6 ppm). All measurements were carried out at ambient temperature. NMR signals were assigned using experimental data (e.g. chemical shifts, coupling constants, integrals where applicable) and with the aid of 2D spectroscopy (TOCSY, NOESY, HSQC, HMBC).

S1.2. Semi-preparative HPLC

Semi-preparative HPLC purification was performed on a KNAUER AZURA Analytical HPLC system (LC-20AT prominence liquid chromatograph, AZURA AS 6.1L auto sampler, UVD 2.1.L UV/vis detector (λ = 220 nm), and an Eurospher II 100-5 C18 reversed phase column (250 mm × 4.6 mm) was used. Solvents A (deionized water with 0.1% trifluoroacetic acid (TFA)) and B (acetonitrile (MeCN) with 0.1% TFA) were utilized in a purification process that involved separation and column washing/equilibration steps according to the following protocol:

1. *Isocratic*: 95 vol.-% A, 5 vol.-% B (min 0 – 30).
2. *Gradient*: 95 vol.-% A to 10 vol.-% A (min 30 – 35).
3. *Isocratic*: 10 vol.-% A, 90 vol.-% B (min 35 – 45).
4. *Gradient*: 10 vol.-% A to 95 vol.-% A (min 45 – 50).
5. *Isocratic*: 95 vol.-% A, 5 vol.-% B (min 50 – 60).

A constant flow rate of 1 ml per min (min 0 – 30) and 3.0 ml per min (min 30 – 60) was applied.

S2. Syntheses of compounds

S2.1. General remarks

All chemicals were purchased from *SIGMA-ALDRICH*, except the 2-chlorotrityl chloride (2-CTC) resin, Fmoc-Pro-OH (*Novabiochem*), and AMUPol (*CortecNet*) and were used as received. For the amino acids, exclusively the L-enantiomers were used.

S2.2 Solid-phase protocol for peptide synthesis

The amounts of reagents of the following synthesis protocol correspond to 77.5 µmol scale.

1) *Swelling*: The 2-CTC-resin was swollen in 1.0 mL dimethylformamide (DMF) for 30 min in a 5-ml-syringe for solid-phase peptide synthesis (SPPS).
2) *Loading of first amino acid*: The Fmoc-amino acid (2.00 eq) was dissolved in N,N-diisopropylethylamine (DIPEA) (8.00 eq) and DMF (0.5 mL). The resulting solution was added to the pre swollen resin and the mixture was shaken on an agitation plate (*RS-OS 5D, PHOENIX Germany Instrument*, 150 rpm) for 2 h. The solution was discarded and the resin was washed with DMF (3 × 2 mL) and dichloromethane (DCM) (3 × 2 mL) and dried under high vacuum (HV) for 30 min. The free binding sites were capped with 0.5 mL of a solution of DCM/MeOH/DIPEA (17:2:1) at RT for 15 min, followed by the washing step mentioned above.
3) *Deprotection of the temporal Fmoc-group*: The resin was treated with 0.5 mL of 40 % piperidine in DMF and sonicated for 15 min. This process was repeated, followed by filtering off the resin and washing steps with DMF (3 × 2 mL) and DCM (3 × 2 mL).
4) *Coupling of amino acids*: The Fmoc-amino acid (2.00 eq), 2-(1*H*-benzotriazol-1-yl)-1,1,3,3-tetramethyluronium-hexafluorophosphate (HBTU) (4.00 eq) and 1-hydroxybenzotriazole (HOBt) (4.00 eq) were dissolved in DIPEA (8.00 eq) and DMF (0.5 mL). The resulting solution was activated for 5 min and added to the resin. This mixture was shaken for 30 min at RT. The solution was discarded and the resin was washed with DMF (3 × 2 mL) and DCM (3 × 2 mL).
5) *Final cleavage of the peptides*: The dried resin was shaken with 0.5 mL of 33 % trifluoroacetic acid (TFA) in DCM for 1 h. This step was repeated, and the resin was washed with 0.5 mL of the TFA solution. The solvent was evaporated under reduced pressure, and 1.0 mL deionized water was added before freeze-drying.
6) *Working up and characterization*: The freeze-dried products were purified via semi-preparative HPLC on an *Knauer Azura* Analytical HPLC system as described before.

S2.2.1. HN-L-$^{13}C_5$,^{15}N-Pro-Gly-OH

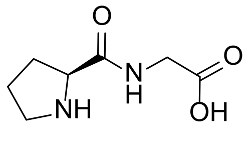

HN-L-$^{13}C_5$,^{15}N-Pro-Gly-OH was synthesized in a 77.5 µmol (1.00 eq) scale on 2-CTC-resin (b = 1.55 mmol/g) according to the general SPPS protocol using Fmoc-L-Gly-OH in step 2) and

Fmoc-L-$^{13}C_5$,^{15}N-Pro-OH in step 4). HN-L-$^{13}C_5$,^{15}N-Pro-Gly-OH × TFA (11.9 mg, 52 %) were obtained as yellow oil.

^{1}H-NMR (500 MHz, d_6-DMSO): δ / ppm = 8.83 (dt, $^2J(^{13}C)$ = 4.51 Hz, $^3J(^1H)$ = 5.72 Hz, 1H, Gly-NH); 8.58 (d, $^1J(^{13}C)$ = 76.6 Hz, 1H, Pro-NH); 4.21 (d, $^1J(^{13}C)$ = 148 Hz, 1H, Pro-α); 3.87 (m, 2H, Gly-α); 3.21 (d, $^1J(^{13}C)$ = 135 Hz, 2H, Pro-δ); 2.31 (d, $^1J(^{13}C)$ = 137 Hz, 1H, Pro-γ); 1.89 (d, $^1J(^{13}C)$ = 134 Hz, 3H, Pro-β/γ).

HPLC t_{ret} / min = 17.2.

S2.2.2. HN-L-$^{13}C_5$,^{15}N-Pro-L-Ala-OH

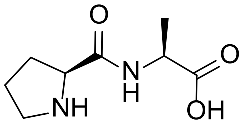

HN-L-$^{13}C_5$,^{15}N-Pro-L-Ala-OH was synthesized in a 77.5 µmol (1.00 eq) scale on 2-CTC-resin (b = 1.55 mmol/g) according to the general protocol SPPS using Fmoc-L-Ala-OH in step 2) and Fmoc-L-$^{13}C_5$,^{15}N-Pro-OH in step 6). HN-L-$^{13}C_5$,^{15}N-Pro-L-Ala-OH × TFA (9.23 mg, 39 %) were obtained as yellow oil.

^{1}H-NMR (500 MHz, d_6-DMSO): δ / ppm = 8.79 (dt, $^2J(^{13}C)$ = 3.69 Hz, $^3J(^1H)$ = 7.39 Hz, 1H, Gly-NH); 8.54 (d, $^1J(^{13}C)$ = 75.3 Hz, 1H, Pro-NH); 4.27 (m, 1H, Ala-α); 4.21 (d, $^1J(^{13}C)$ = 147 Hz, 1H, Pro-α); 3.21 (d, $^1J(^{13}C)$ = 145 Hz, 2H, Pro-δ); 2.31 (d, $^1J(^{13}C)$ = 136 Hz, 1H, Pro-γ); 1.89 (d, $^1J(^{13}C)$ = 132 Hz, 3H, Pro-β/γ); 1.32 (d, $^3J(^1H)$ = 7.39 Hz, 3H, Ala-β).

HPLC t_{ret} / min = 17.2.

S2.3. Liquid-phase protocol for peptide synthesis

HN-$^{13}C_5$,^{15}N-L-Pro-OH (23.0 mg, 0.2 mmol, 1.00 eq) was suspended in DMF (2 mL) in a 10 mL round flask. Fmoc-Xaa-OH (0.3 mmol, 1.50 eq), where Xaa refers to either Gly or Ala, was dissolved in 2 mL DMF together with HBTU (106.2 mg, 0.28 mmol, 1.40 eq) and DIPEA (136.1 µL, 0.8 mmol, 4.00 eq) and the solution was shaken for 10 min. The Fmoc-Xaa solution was added dropwise to the Pro solution. The solution was stirred for 60 min. Afterwards the solvent was removed under reduced pressure (5 mbar, 60 °C). A solution of 20% Piperidine in DMF was added to resuspend/resolve the raw product and stirred for 15 min. The solvent was removed under reduced pressure (5 mbar, 60 °C) and the raw product was resuspend in 2 mL pure water and transferred to a 50 ml falcon tube. The round flask was washed 3 times with 2 mL water. The collected water phase was centrifuged and decanted. The resulting aqueous solution was frozen and lyophilized. The resulting raw product was purified using the above mentioned HPLC protocol.

S2.3.1. Gly-$^{13}C_5$,^{15}N-L-Pro

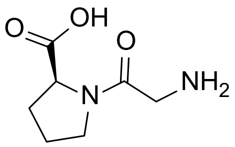

Gly-$^{13}C_5$,^{15}N-L-Pro was synthesized according to the protocol for liquid phase peptide synthesis. 89.1 mg Fmoc-Gly-OH were used.

^{1}H-NMR (500 MHz, d_6-DMSO): δ / ppm = 8.80 (t, ^3J = 7.39 Hz, 1H, Gly-NH); 8.54 (s, 1H, Pro-NH); 4.27 (m, 1H, Ala-α); 4.18 (m, 1H, Pro-α); 3.22 (m, 2H, Pro-δ); 2.30 (m, 1H, Pro-γ); 1.88 (m, 3H, Pro-β/γ); 1.32 (d, ^3J = 7.39 Hz, 3H, Ala-β).

HPLC t_{ret} / min = 17.2.

S2.3.2. L-Ala-$^{13}C_5$,^{15}N-L-Pro

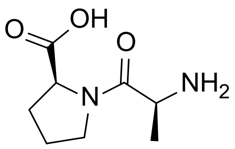

L-Ala-$^{13}C_5$,^{15}N-L-Pro was synthesized according to the protocol for liquid phase peptide synthesis. 93.2 mg Fmoc-Ala-OH were used.

^{1}H-NMR (500 MHz, d_6-DMSO): δ / ppm = 8.80 (t, ^3J = 7.39 Hz, 1H, Gly-NH); 8.54 (s, 1H, Pro-NH); 4.27 (m, 1H, Ala-α); 4.18 (m, 1H, Pro-α); 3.22 (m, 2H, Pro-δ); 2.30 (m, 1H, Pro-γ); 1.88 (m, 3H, Pro-β/γ); 1.32 (d, ^3J = 7.39 Hz, 3H, Ala-β).

HPLC t_{ret} / min = 17.2.

S3. NMR data analysis

S3.1. Enhancements

Table S3.1.1. DNP enhancement factors for ^{1}H-^{13}C CP NMR spectra for proline or derivative (100 mM) at 100 K, 125 K and 150 K and ν_{MAS}= 18 kHz

Sample	*T* / K	ε
$^{13}C_5$,^{15}N-L-Pro	100	181
	125	128
	150	107
$^{13}C_5$,^{15}N-L-Pro-L-Ala	100	150
	150	104
$^{13}C_5$,^{15}N-L-Pro-Gly	100	193
	150	117
Gly-$^{13}C_5$,^{15}N-L-Pro	100	195
	150	100
L-Ala-$^{13}C_5$,^{15}N-L-Pro	100	152
	150	100
L-Pro	100	90

S3.2. Spectral decomposition

This section provides an overview of all fitting parameters obtained from the spectral decomposition into single Voigtian line shapes of ΔDP_{sat} spectra for all analyzed species. Additionally, the full width at half maximum (FWHM) was calculated for the Lorentzian, Gaussian, and Voigtian components of each signal using line-broadening parameters obtained from fitting, according to:

$$FWHM_{Gauss} = 2 * \sqrt{2\ln(2)} * \sigma$$

$$FWHM_{Lorentz} = 2\gamma$$

$$FWHM_{Voigt} = 0.5346 * 2\gamma + \sqrt{0.2166 * (2\gamma)^2 + 4\ln(2) * \sigma^2}$$

S3.2.1. $^{13}C_5$,^{15}N-L-Pro at 100 K

Table S3.2.1: Parameters obtained from spectral simulation/deconvolution of ΔDP_{sat} spectra for $^{13}C_5$,^{15}N-L-Pro at 100 K and 18 kHz MAS frequency. All five resonances were fitted to a Voigtian lineshape, with σ and γ as the Gaussian and Lorentzian line shape parameters, respectively. FWHM parameters were calculated using the previously given formulas (SI S3.2) from the corresponding line broadening parameters. The integrals were obtained by numerical integration of the simulated peaks.

Label	Time / s	Center / ppm	Amplitude / a.u.	σ / ppm	γ / ppm	FWHM Lorentz / ppm	FWHM Gauss / ppm	FWHM Voigt / ppm	Integral / a.u.
**CO**	1.0	175.248	400.093	1.722	0.289	0.578	4.056	3.189	15774.733
	4.0	175.248	3205.771	1.722	0.289	0.578	4.056	3.189	126396.005
	8.0	175.248	6411.827	1.722	0.289	0.578	4.056	3.189	252803.215
	16.0	175.248	10197.044	1.722	0.289	0.578	4.056	3.189	402045.395
	32.0	175.248	14067.76	1.722	0.289	0.578	4.056	3.189	554658.621
	64.0	175.248	18173.306	1.722	0.289	0.578	4.056	3.189	716530.624
	128.0	175.248	22667.099	1.722	0.289	0.578	4.056	3.189	893710.281
	256.0	175.248	27806.058	1.722	0.289	0.578	4.056	3.189	1096327.338
	512.0	175.248	33136.117	1.722	0.289	0.578	4.056	3.189	1306478.974
**Cα**	1.0	61.075	432.917	1.576	0.258	0.517	3.711	2.911	17108.647
	4.0	61.075	3557.401	1.576	0.258	0.517	3.711	2.911	140586.69
	8.0	61.075	6918.228	1.576	0.258	0.517	3.711	2.911	273404.896
	16.0	61.075	10530.817	1.576	0.258	0.517	3.711	2.911	416172.584
	32.0	61.075	14080.34	1.576	0.258	0.517	3.711	2.911	556447.942
	64.0	61.075	18164.39	1.576	0.258	0.517	3.711	2.911	717847.537
	128.0	61.075	22591.66	1.576	0.258	0.517	3.711	2.911	892811.032
	256.0	61.075	27151.438	1.576	0.258	0.517	3.711	2.911	1073011.183
	512.0	61.075	32662.591	1.576	0.258	0.517	3.711	2.911	1290809.149
**Cδ**	1.0	46.27	495.509	1.376	0.611	1.223	3.241	3.015	19512.87
	4.0	46.27	3794.835	1.376	0.611	1.223	3.241	3.015	149438.547
	8.0	46.27	6991.577	1.376	0.611	1.223	3.241	3.015	275324.532
	16.0	46.27	10676.097	1.376	0.611	1.223	3.241	3.015	420418.923
	32.0	46.27	14491.869	1.376	0.611	1.223	3.241	3.015	570681.937
	64.0	46.27	18428.961	1.376	0.611	1.223	3.241	3.015	725722.49
	128.0	46.27	22628.299	1.376	0.611	1.223	3.241	3.015	891090.16
	256.0	46.27	27301.892	1.376	0.611	1.223	3.241	3.015	1075133.71
	512.0	46.27	32274.436	1.376	0.611	1.223	3.241	3.015	1270949.788
**Cβ**	1.0	30.0	427.195	1.237	0.568	1.136	2.913	2.734	16795.193
	4.0	30.0	3428.537	1.237	0.568	1.136	2.913	2.734	134793.232
	8.0	30.0	6518.587	1.237	0.568	1.136	2.913	2.734	256278.783
	16.0	30.0	9888.178	1.237	0.568	1.136	2.913	2.734	388754.561
	32.0	30.0	13247.743	1.237	0.568	1.136	2.913	2.734	520836.117
	64.0	30.0	16970.191	1.237	0.568	1.136	2.913	2.734	667184.482
	128.0	30.0	20885.285	1.237	0.568	1.136	2.913	2.734	821106.752
	256.0	30.0	25029.984	1.237	0.568	1.136	2.913	2.734	984055.936
	512.0	30.0	30088.827	1.237	0.568	1.136	2.913	2.734	1182944.782
**Cγ**	1.0	24.819	517.699	2.035	0.0	0.0	4.792	3.389	20498.929
	4.0	24.819	3739.093	2.035	0.0	0.0	4.792	3.389	148054.133
	8.0	24.819	6941.012	2.035	0.0	0.0	4.792	3.389	274838.175
	16.0	24.819	10602.703	2.035	0.0	0.0	4.792	3.389	419827.46
	32.0	24.819	14487.219	2.035	0.0	0.0	4.792	3.389	573639.804
	64.0	24.819	18409.579	2.035	0.0	0.0	4.792	3.389	728950.61
	128.0	24.819	22620.295	2.035	0.0	0.0	4.792	3.389	895679.228
	256.0	24.819	27702.93	2.035	0.0	0.0	4.792	3.389	1096932.601
	512.0	24.819	32326.427	2.035	0.0	0.0	4.792	3.389	1280005.825

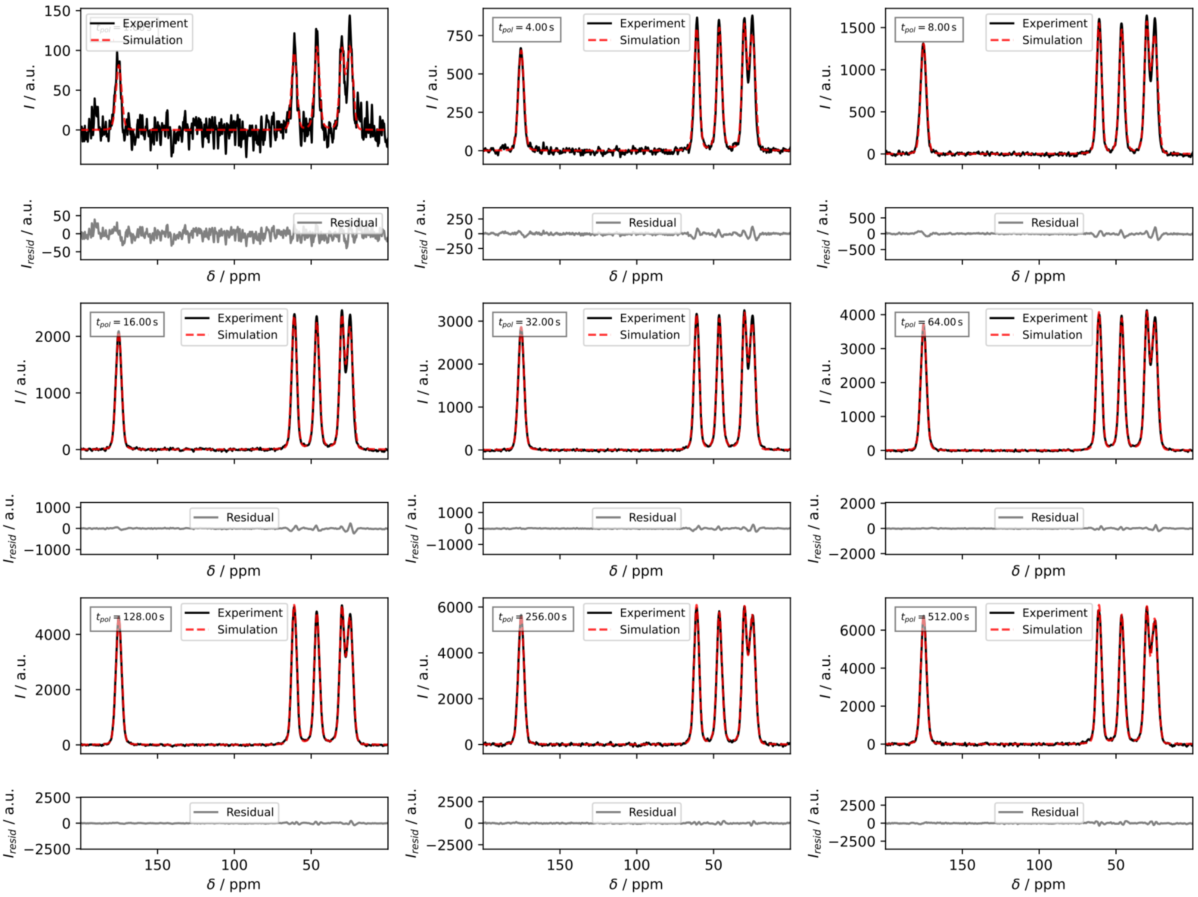

Figure S3.2.1: Number of scan normalized experimental (black) and simulated (red) DNP-enhanced ^{13}C MAS NMR ΔDP_{sat} spectra of 100 mM ^{13}C,^{15}N-L-Pro (100 mM, 18 kHz MAS and 100 K) at different polarizing times (t_{pol}). Each panel represents a spectrum with increasing t_{pol} from top left to bottom right. The residuals are shown in the lower part of each panel. Each resonance was fitted to an Voigtian lineshape and the line broadening parameter were fitted globally over all polarizing times (t_{pol}). Fitting parameters are given in Table S3.2.1.

S3.2.2. $^{13}C_5$,^{15}N-L-Pro at 125 K

Table S3.2.2: Parameters obtained from spectral simulation/deconvolution of ΔDP_{sat} spectra for $^{13}C_5$,^{15}N-L-Pro at 125 K and 18 kHz MAS frequency. All five resonances were fitted to a Voigtian lineshape, with σ and γ as the Gaussian and Lorentzian line shape parameters, respectively. FWHM parameters were calculated using the previously given formulas (SI S3.2) from the corresponding line broadening parameters. The integrals were obtained by numerical integration of the simulated peaks.

Label	Time / s	Center / ppm	Amplitude / a.u.	σ / ppm	γ / ppm	FWHM Lorentz / ppm	FWHM Gauss / ppm	FWHM Voigt / ppm	Integral / a.u.
**CO**	1.0	174.982	250.895	1.72	0.344	0.687	4.049	3.249	9884.575
	4.0	174.982	3180.662	1.72	0.344	0.687	4.049	3.249	125309.323
	8.0	174.982	6552.772	1.72	0.344	0.687	4.049	3.249	258161.153
	16.0	174.982	10330.281	1.72	0.344	0.687	4.049	3.249	406984.602
	32.0	174.982	13778.65	1.72	0.344	0.687	4.049	3.249	542840.844
	64.0	174.982	17711.197	1.72	0.344	0.687	4.049	3.249	697772.36
	128.0	174.982	21475.43	1.72	0.344	0.687	4.049	3.249	846072.788
	256.0	174.982	25734.364	1.72	0.344	0.687	4.049	3.249	1013863.022
	512.0	174.982	30286.367	1.72	0.344	0.687	4.049	3.249	1193199.43
**Cα**	1.0	60.763	467.59	1.576	0.521	1.042	3.71	3.225	18442.097
	4.0	60.763	3414.338	1.576	0.521	1.042	3.71	3.225	134664.033
	8.0	60.763	6769.27	1.576	0.521	1.042	3.71	3.225	266985.041
	16.0	60.763	10275.107	1.576	0.521	1.042	3.71	3.225	405257.848
	32.0	60.763	13652.679	1.576	0.521	1.042	3.71	3.225	538471.82
	64.0	60.763	17277.055	1.576	0.521	1.042	3.71	3.225	681419.899
	128.0	60.763	21151.972	1.576	0.521	1.042	3.71	3.225	834249.494
	256.0	60.763	25078.058	1.576	0.521	1.042	3.71	3.225	989097.246
	512.0	60.763	28820.943	1.576	0.521	1.042	3.71	3.225	1136719.433
**Cδ**	1.0	46.106	538.314	1.354	0.979	1.959	3.187	3.478	21127.725
	4.0	46.106	3538.716	1.354	0.979	1.959	3.187	3.478	138887.442
	8.0	46.106	6992.959	1.354	0.979	1.959	3.187	3.478	274459.514
	16.0	46.106	10406.255	1.354	0.979	1.959	3.187	3.478	408424.483
	32.0	46.106	13778.965	1.354	0.979	1.959	3.187	3.478	540796.511
	64.0	46.106	17366.618	1.354	0.979	1.959	3.187	3.478	681604.668
	128.0	46.106	21078.141	1.354	0.979	1.959	3.187	3.478	827274.408
	256.0	46.106	24947.731	1.354	0.979	1.959	3.187	3.478	979148.002
	512.0	46.106	28795.603	1.354	0.979	1.959	3.187	3.478	1130169.203
**Cβ**	1.0	29.761	573.311	1.217	1.009	2.018	2.866	3.313	22412.678
	4.0	29.761	3435.533	1.217	1.009	2.018	2.866	3.313	134306.675
	8.0	29.761	6831.319	1.217	1.009	2.018	2.866	3.313	267059.489
	16.0	29.761	10060.121	1.217	1.009	2.018	2.866	3.313	393284.318
	32.0	29.761	13419.96	1.217	1.009	2.018	2.866	3.313	524631.864
	64.0	29.761	16806.425	1.217	1.009	2.018	2.866	3.313	657020.279
	128.0	29.761	20230.854	1.217	1.009	2.018	2.866	3.313	790892.825
	256.0	29.761	23792.724	1.217	1.009	2.018	2.866	3.313	930138.441
	512.0	29.761	27207.5	1.217	1.009	2.018	2.866	3.313	1063633.661
**Cγ**	1.0	24.596	428.757	1.828	0.625	1.249	4.304	3.766	16819.848
	4.0	24.596	3662.528	1.828	0.625	1.249	4.304	3.766	143678.639
	8.0	24.596	6741.73	1.828	0.625	1.249	4.304	3.766	264473.778
	16.0	24.596	10571.924	1.828	0.625	1.249	4.304	3.766	414729.873
	32.0	24.596	13758.89	1.828	0.625	1.249	4.304	3.766	539752.545
	64.0	24.596	17316.308	1.828	0.625	1.249	4.304	3.766	679307.771
	128.0	24.596	21363.092	1.828	0.625	1.249	4.304	3.766	838060.549
	256.0	24.596	25730.316	1.828	0.625	1.249	4.304	3.766	1009383.98
	512.0	24.596	29726.606	1.828	0.625	1.249	4.304	3.766	1166155.884

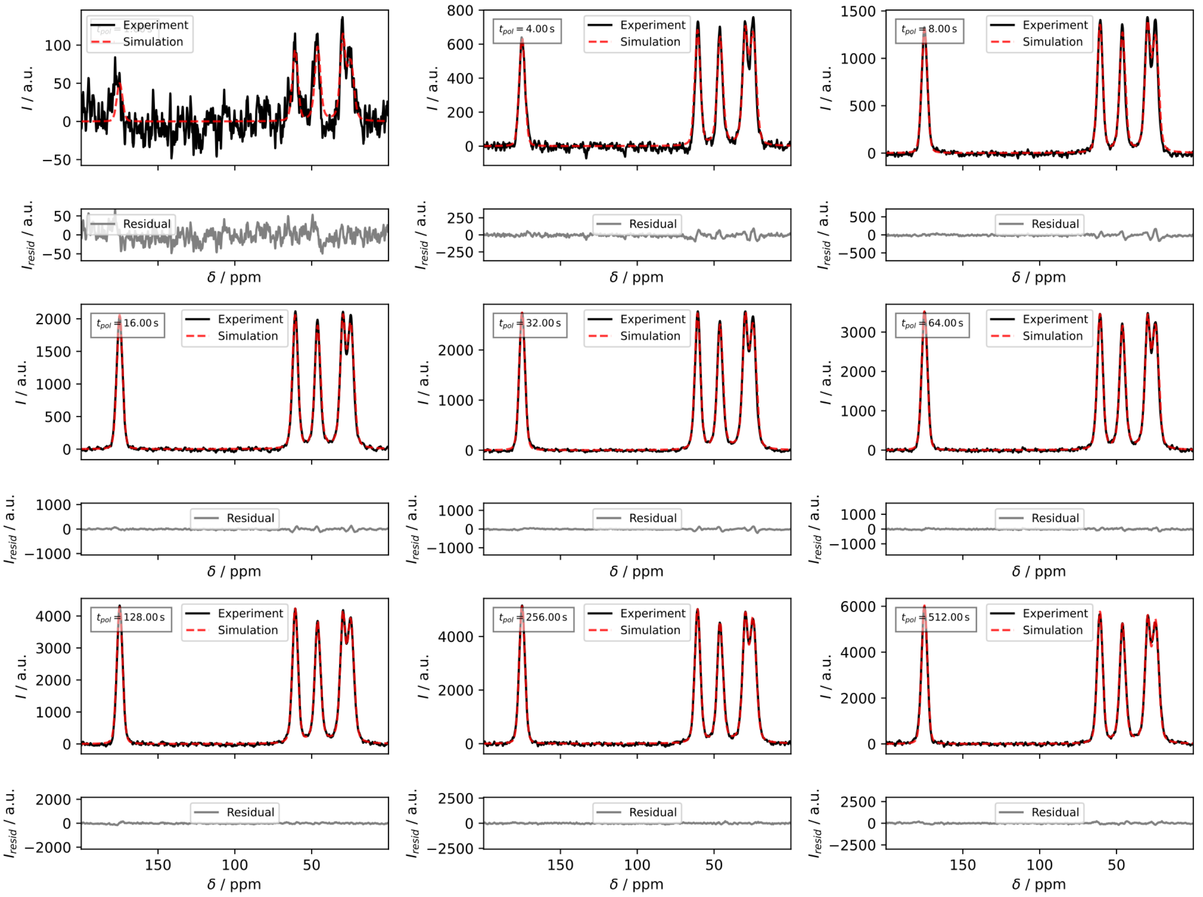

Figure S3.2.2: Number of scan normalized experimental (black) and simulated (red) DNP-enhanced ^{13}C MAS NMR ΔDP_{sat} spectra of 100 mM $^{13}C,^{15}N$-L-Pro (100 mM, 18 kHz MAS and 125 K) at different polarizing times (t_{pol}). Each panel represents a spectrum with increasing t_{pol} from top left to bottom right. The residuals are shown in the lower part of each panel. Each resonance was fitted to an Voigtian lineshape and the line broadening parameter were fitted globally over all polarizing times (t_{pol}). Fitting parameters are given in Table S3.2.2.

S3.2.3. $^{13}C_5$,^{15}N-L-Pro at 150 K

Table S3.2.3: Parameters obtained from spectral simulation/deconvolution of ΔDP_{sat} spectra for $^{13}C_5$,^{15}N-L-Pro at 150 K and 18 kHz MAS frequency. All five resonances were fitted to a Voigtian lineshape, with σ and γ as the Gaussian and Lorentzian line shape parameters, respectively. FWHM parameters were calculated using the previously given formulas (SI S3.2) from the corresponding line broadening parameters. The integrals were obtained by numerical integration of the simulated peaks.

Label	Time / s	Center / ppm	Amplitude / a.u.	σ / ppm	γ / ppm	FWHM Lorentz / ppm	FWHM Gauss / ppm	FWHM Voigt / ppm	Integral / a.u.
CO	1.0	175.03	407.317	1.657	0.391	0.781	3.901	3.2	16036.04
	4.0	175.03	3601.344	1.657	0.391	0.781	3.901	3.2	141784.66
	8.0	175.03	7307.359	1.657	0.391	0.781	3.901	3.2	287690.213
	16.0	175.03	11373.977	1.657	0.391	0.781	3.901	3.2	447792.706
	32.0	175.03	15145.879	1.657	0.391	0.781	3.901	3.2	596292.215
	64.0	175.03	19211.214	1.657	0.391	0.781	3.901	3.2	756344.177
	128.0	175.03	23394.983	1.657	0.391	0.781	3.901	3.2	921058.854
	256.0	175.03	27218.402	1.657	0.391	0.781	3.901	3.2	1071586.603
	512.0	175.03	30502.123	1.657	0.391	0.781	3.901	3.2	1200866.47
Cα	1.0	60.91	530.401	1.416	0.627	1.254	3.334	3.099	20902.831
	4.0	60.91	3935.245	1.416	0.627	1.254	3.334	3.099	155086.064
	8.0	60.91	7466.924	1.416	0.627	1.254	3.334	3.099	294267.806
	16.0	60.91	11494.166	1.416	0.627	1.254	3.334	3.099	452979.417
	32.0	60.91	15381.454	1.416	0.627	1.254	3.334	3.099	606175.536
	64.0	60.91	18863.124	1.416	0.627	1.254	3.334	3.099	743386.46
	128.0	60.91	22315.286	1.416	0.627	1.254	3.334	3.099	879434.431
	256.0	60.91	26373.271	1.416	0.627	1.254	3.334	3.099	1039357.643
	512.0	60.91	29110.383	1.416	0.627	1.254	3.334	3.099	1147225.861
Cδ	1.0	46.392	609.509	1.212	1.048	2.095	2.855	3.362	23908.209
	4.0	46.392	3943.839	1.212	1.048	2.095	2.855	3.362	154698.471
	8.0	46.392	7672.103	1.212	1.048	2.095	2.855	3.362	300940.938
	16.0	46.392	11562.514	1.212	1.048	2.095	2.855	3.362	453543.697
	32.0	46.392	15316.792	1.212	1.048	2.095	2.855	3.362	600806.591
	64.0	46.392	18885.5	1.212	1.048	2.095	2.855	3.362	740790.433
	128.0	46.392	22236.168	1.212	1.048	2.095	2.855	3.362	872221.563
	256.0	46.392	26060.009	1.212	1.048	2.095	2.855	3.362	1022213.069
	512.0	46.392	28564.409	1.212	1.048	2.095	2.855	3.362	1120449.066
Cβ	1.0	30.0	515.796	1.072	1.072	2.144	2.525	3.191	20149.993
	4.0	30.0	3918.157	1.072	1.072	2.144	2.525	3.191	153066.112
	8.0	30.0	7490.518	1.072	1.072	2.144	2.525	3.191	292623.422
	16.0	30.0	11319.99	1.072	1.072	2.144	2.525	3.191	442224.969
	32.0	30.0	15139.713	1.072	1.072	2.144	2.525	3.191	591445.682
	64.0	30.0	18351.306	1.072	1.072	2.144	2.525	3.191	716909.299
	128.0	30.0	21061.918	1.072	1.072	2.144	2.525	3.191	822801.635
	256.0	30.0	25260.846	1.072	1.072	2.144	2.525	3.191	986836.334
	512.0	30.0	26796.38	1.072	1.072	2.144	2.525	3.191	1046823.27
Cγ	1.0	24.878	670.811	1.767	0.643	1.286	4.161	3.69	26310.742
	4.0	24.878	3914.184	1.767	0.643	1.286	4.161	3.69	153523.324
	8.0	24.878	7303.827	1.767	0.643	1.286	4.161	3.69	286472.924
	16.0	24.878	10931.292	1.767	0.643	1.286	4.161	3.69	428750.456
	32.0	24.878	14268.627	1.767	0.643	1.286	4.161	3.69	559648.446
	64.0	24.878	18423.795	1.767	0.643	1.286	4.161	3.69	722623.683
	128.0	24.878	23399.471	1.767	0.643	1.286	4.161	3.69	917781.177
	256.0	24.878	25657.177	1.767	0.643	1.286	4.161	3.69	1006333.589
	512.0	24.878	29719.838	1.767	0.643	1.286	4.161	3.69	1165680.512

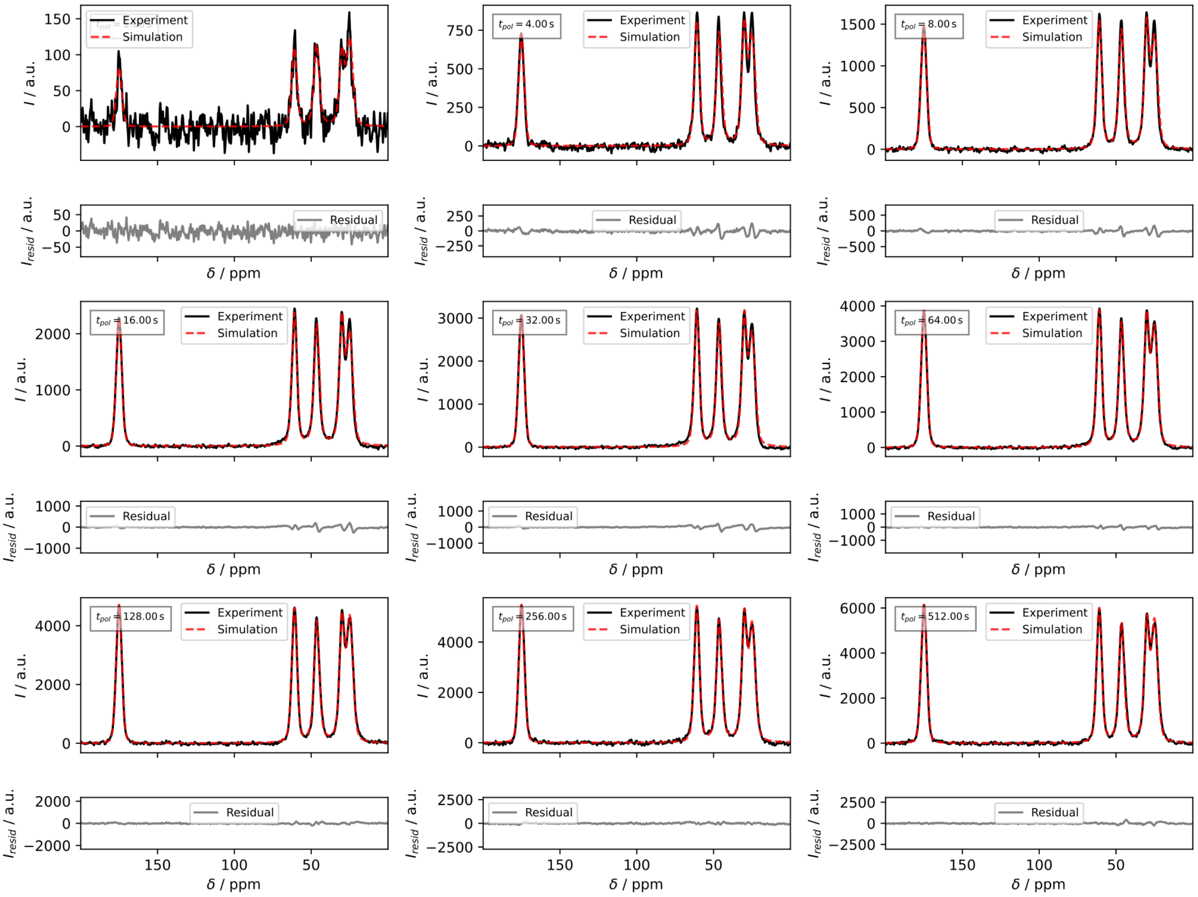

Figure S3.2.3: Number of scan normalized experimental (black) and simulated (red) DNP-enhanced ^{13}C MAS NMR ΔDP_{sat} spectra of 100 mM ^{13}C,^{15}N-L-Pro (100 mM, 18 kHz MAS and 150 K) at different polarizing times (t_{pol}). Each panel represents a spectrum with increasing t_{pol} from top left to bottom right. The residuals are shown in the lower part of each panel. Each resonance was fitted to an Voigtian lineshape and the line broadening parameter were fitted globally over all polarizing times (t_{pol}). Fitting parameters are given in Table S3.2.3.

S3.2.4. $^{13}C_5$,^{15}N-L-Pro-Gly at 100 K

Table S3.2.4: Parameters obtained from spectral simulation/deconvolution of ΔDP_{sat} spectra for $^{13}C_5$,^{15}N-L-Pro-Gly at 100 K and 18 kHz MAS frequency. All five resonances were fitted to a Voigtian lineshape, with σ and γ as the Gaussian and Lorentzian line shape parameters, respectively. FWHM parameters were calculated using the previously given formulas (SI S3.2) from the corresponding line broadening parameters. The integrals were obtained by numerical integration of the simulated peaks.

Label	Time / s	Center / ppm	Amplitude / a.u.	σ / ppm	γ / ppm	FWHM Lorentz / ppm	FWHM Gauss / ppm	FWHM Voigt / ppm	Integral / a.u.
**CO**	1.0	172.092	478.563	1.78	0.255	0.511	4.192	3.247	18884.949
	4.0	172.092	2724.471	1.78	0.255	0.511	4.192	3.247	107512.372
	8.0	172.092	5807.394	1.78	0.255	0.511	4.192	3.247	229169.881
	16.0	172.092	9342.041	1.78	0.255	0.511	4.192	3.247	368653.195
	32.0	172.092	12785.339	1.78	0.255	0.511	4.192	3.247	504531.735
	64.0	172.092	16555.657	1.78	0.255	0.511	4.192	3.247	653315.036
	128.0	172.092	20677.453	1.78	0.255	0.511	4.192	3.247	815968.305
	256.0	172.092	25561.748	1.78	0.255	0.511	4.192	3.247	1008711.066
	512.0	172.092	30504.246	1.78	0.255	0.511	4.192	3.247	1203750.649
**Cα**	1.0	61.014	325.434	1.639	0.147	0.294	3.859	2.889	12871.733
	4.0	61.014	3082.53	1.639	0.147	0.294	3.859	2.889	121921.917
	8.0	61.014	6063.54	1.639	0.147	0.294	3.859	2.889	239828.516
	16.0	61.014	9469.25	1.639	0.147	0.294	3.859	2.889	374533.031
	32.0	61.014	12656.368	1.639	0.147	0.294	3.859	2.889	500591.69
	64.0	61.014	16342.617	1.639	0.147	0.294	3.859	2.889	646392.246
	128.0	61.014	20314.194	1.639	0.147	0.294	3.859	2.889	803478.282
	256.0	61.014	24927.878	1.639	0.147	0.294	3.859	2.889	985961.255
	512.0	61.014	30058.251	1.639	0.147	0.294	3.859	2.889	1188880.624
**Cδ**	1.0	47.892	408.128	1.373	0.729	1.457	3.233	3.163	16057.397
	4.0	47.892	3377.262	1.373	0.729	1.457	3.233	3.163	132874.963
	8.0	47.892	6627.608	1.373	0.729	1.457	3.233	3.163	260756.507
	16.0	47.892	10154.845	1.373	0.729	1.457	3.233	3.163	399532.079
	32.0	47.892	13541.018	1.373	0.729	1.457	3.233	3.163	532757.617
	64.0	47.892	17334.526	1.373	0.729	1.457	3.233	3.163	682009.318
	128.0	47.892	21443.951	1.373	0.729	1.457	3.233	3.163	843690.482
	256.0	47.892	26007.506	1.373	0.729	1.457	3.233	3.163	1023238.904
	512.0	47.892	31259.286	1.373	0.729	1.457	3.233	3.163	1229864.85
**Cβ**	1.0	32.035	343.292	1.411	0.448	0.896	3.322	2.864	13520.948
	4.0	32.035	2928.128	1.411	0.448	0.896	3.322	2.864	115327.558
	8.0	32.035	5732.42	1.411	0.448	0.896	3.322	2.864	225777.671
	16.0	32.035	8868.19	1.411	0.448	0.896	3.322	2.864	349283.423
	32.0	32.035	11887.229	1.411	0.448	0.896	3.322	2.864	468191.621
	64.0	32.035	15278.67	1.411	0.448	0.896	3.322	2.864	601767.25
	128.0	32.035	18846.446	1.411	0.448	0.896	3.322	2.864	742288.029
	256.0	32.035	22841.548	1.411	0.448	0.896	3.322	2.864	899639.548
	512.0	32.035	27605.036	1.411	0.448	0.896	3.322	2.864	1087254.76
**Cγ**	1.0	26.076	468.964	1.85	0.287	0.575	4.356	3.399	18494.006
	4.0	26.076	3401.978	1.85	0.287	0.575	4.356	3.399	134160.042
	8.0	26.076	6544.45	1.85	0.287	0.575	4.356	3.399	258086.226
	16.0	26.076	10049.916	1.85	0.287	0.575	4.356	3.399	396327.426
	32.0	26.076	13668.156	1.85	0.287	0.575	4.356	3.399	539015.935
	64.0	26.076	17339.81	1.85	0.287	0.575	4.356	3.399	683810.889
	128.0	26.076	21661.903	1.85	0.287	0.575	4.356	3.399	854256.458
	256.0	26.076	26302.473	1.85	0.287	0.575	4.356	3.399	1037261.511
	512.0	26.076	31761.477	1.85	0.287	0.575	4.356	3.399	1252542.2

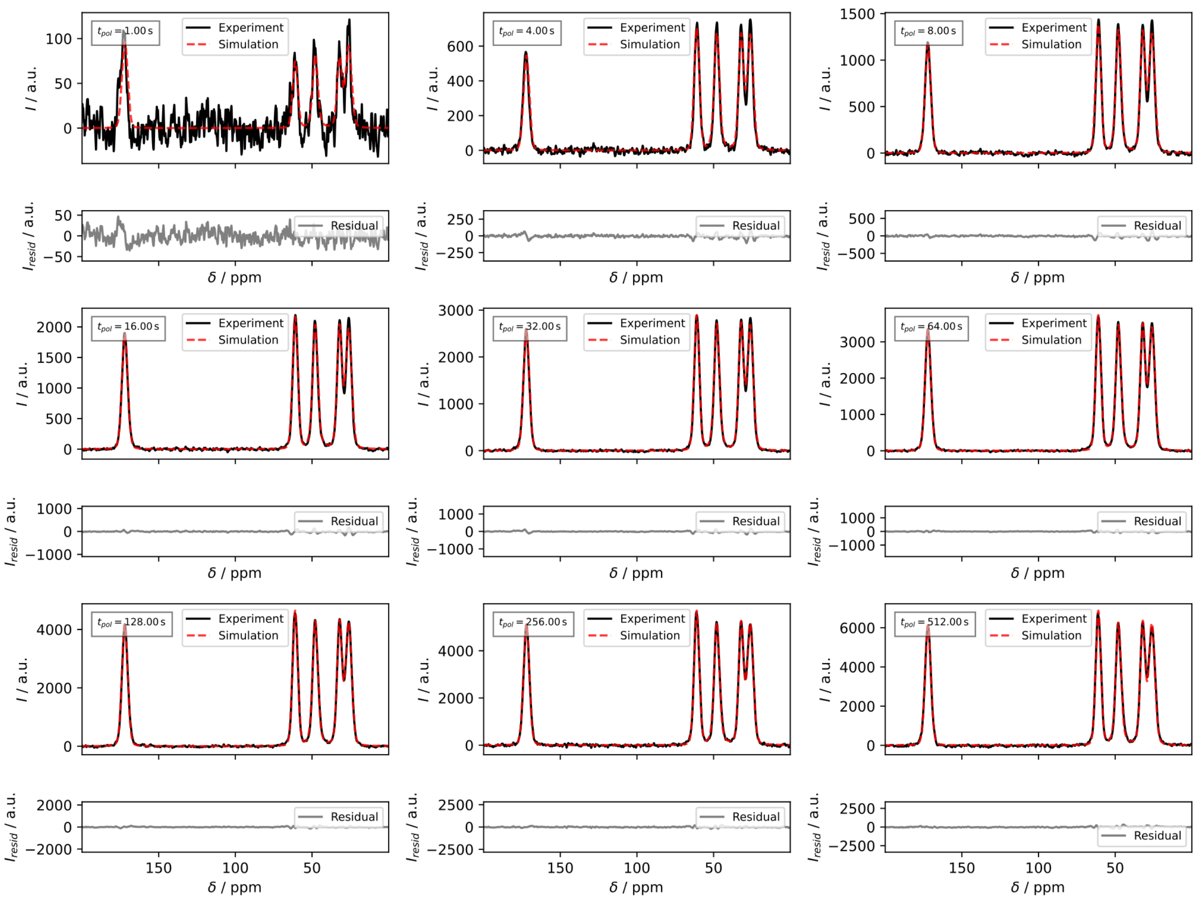

Figure S3.2.4: Number of scan normalized experimental (black) and simulated (red) DNP-enhanced ^{13}C MAS NMR ΔDP_{sat} spectra of 100 mM ^{13}C,^{15}N-L-Pro-Gly (100 mM, 18 kHz MAS and 100 K) at different polarizing times (t_{pol}). Each panel represents a spectrum with increasing t_{pol} from top left to bottom right. The residuals are shown in the lower part of each panel. Each resonance was fitted to an Voigtian lineshape and the line broadening parameter were fitted globally over all polarizing times (t_{pol}). Fitting parameters are given in Table S3.2.4.

S3.2.5. $^{13}C_5$,^{15}N-L-Pro-Gly at 150 K

Table S3.2.5: Parameters obtained from spectral simulation/deconvolution of ΔDP_{sat} spectra for $^{13}C_5$,^{15}N-L-Pro-Gly at 150 K and 18 kHz MAS frequency. All five resonances were fitted to a Voigtian lineshape, with σ and γ as the Gaussian and Lorentzian line shape parameters, respectively. FWHM parameters were calculated using the previously given formulas (SI S3.2) from the corresponding line broadening parameters. The integrals were obtained by numerical integration of the simulated peaks.

Label	Time / s	Center / ppm	Amplitude / a.u.	σ / ppm	γ / ppm	FWHM Lorentz / ppm	FWHM Gauss / ppm	FWHM Voigt / ppm	Integral / a.u.
CO	1.0	172.413	166.432	1.6	0.476	0.952	3.767	3.209	6547.917
	4.0	172.413	2541.299	1.6	0.476	0.952	3.767	3.209	99982.314
	8.0	172.413	5911.144	1.6	0.476	0.952	3.767	3.209	232562.098
	16.0	172.413	9921.041	1.6	0.476	0.952	3.767	3.209	390323.453
	32.0	172.413	13594.168	1.6	0.476	0.952	3.767	3.209	534835.278
	64.0	172.413	17340.512	1.6	0.476	0.952	3.767	3.209	682227.692
	128.0	172.413	19758.856	1.6	0.476	0.952	3.767	3.209	777372.566
	256.0	172.413	23383.103	1.6	0.476	0.952	3.767	3.209	919961.301
	512.0	172.413	26986.239	1.6	0.476	0.952	3.767	3.209	1061719.477
Cα	1.0	61.288	438.172	1.495	0.523	1.047	3.519	3.095	17281.824
	4.0	61.288	2997.223	1.495	0.523	1.047	3.519	3.095	118212.752
	8.0	61.288	5820.71	1.495	0.523	1.047	3.519	3.095	229573.201
	16.0	61.288	9526.3	1.495	0.523	1.047	3.519	3.095	375724.489
	32.0	61.288	12945.598	1.495	0.523	1.047	3.519	3.095	510584.192
	64.0	61.288	16433.482	1.495	0.523	1.047	3.519	3.095	648148.942
	128.0	61.288	18781.266	1.495	0.523	1.047	3.519	3.095	740747.345
	256.0	61.288	22075.011	1.495	0.523	1.047	3.519	3.095	870655.129
	512.0	61.288	25236.658	1.495	0.523	1.047	3.519	3.095	995352.884
Cδ	1.0	48.41	341.826	1.063	1.256	2.511	2.503	3.464	13387.429
	4.0	48.41	3294.537	1.063	1.256	2.511	2.503	3.464	129028.876
	8.0	48.41	6270.302	1.063	1.256	2.511	2.503	3.464	245573.17
	16.0	48.41	10119.177	1.063	1.256	2.511	2.503	3.464	396312.4
	32.0	48.41	13869.065	1.063	1.256	2.511	2.503	3.464	543174.879
	64.0	48.41	17504.238	1.063	1.256	2.511	2.503	3.464	685544.553
	128.0	48.41	19569.363	1.063	1.256	2.511	2.503	3.464	766424.129
	256.0	48.41	22511.465	1.063	1.256	2.511	2.503	3.464	881650.069
	512.0	48.41	25801.668	1.063	1.256	2.511	2.503	3.464	1010509.198
Cβ	1.0	32.444	282.96	1.18	0.973	1.947	2.779	3.205	11076.209
	4.0	32.444	2996.708	1.18	0.973	1.947	2.779	3.205	117303.326
	8.0	32.444	5822.76	1.18	0.973	1.947	2.779	3.205	227926.483
	16.0	32.444	9465.694	1.18	0.973	1.947	2.779	3.205	370525.738
	32.0	32.444	12595.978	1.18	0.973	1.947	2.779	3.205	493057.756
	64.0	32.444	15672.035	1.18	0.973	1.947	2.779	3.205	613467.141
	128.0	32.444	17722.497	1.18	0.973	1.947	2.779	3.205	693730.53
	256.0	32.444	20280.912	1.18	0.973	1.947	2.779	3.205	793877.281
	512.0	32.444	23300.385	1.18	0.973	1.947	2.779	3.205	912071.728
Cγ	1.0	26.567	357.445	1.697	0.73	1.459	3.997	3.687	14010.186
	4.0	26.567	2843.803	1.697	0.73	1.459	3.997	3.687	111463.728
	8.0	26.567	5886.606	1.697	0.73	1.459	3.997	3.687	230727.339
	16.0	26.567	9824.566	1.697	0.73	1.459	3.997	3.687	385076.871
	32.0	26.567	13395.426	1.697	0.73	1.459	3.997	3.687	525037.844
	64.0	26.567	17212.782	1.697	0.73	1.459	3.997	3.687	674660.29
	128.0	26.567	18726.627	1.697	0.73	1.459	3.997	3.687	733995.906
	256.0	26.567	22469.287	1.697	0.73	1.459	3.997	3.687	880690.594
	512.0	26.567	26124.122	1.697	0.73	1.459	3.997	3.687	1023942.963

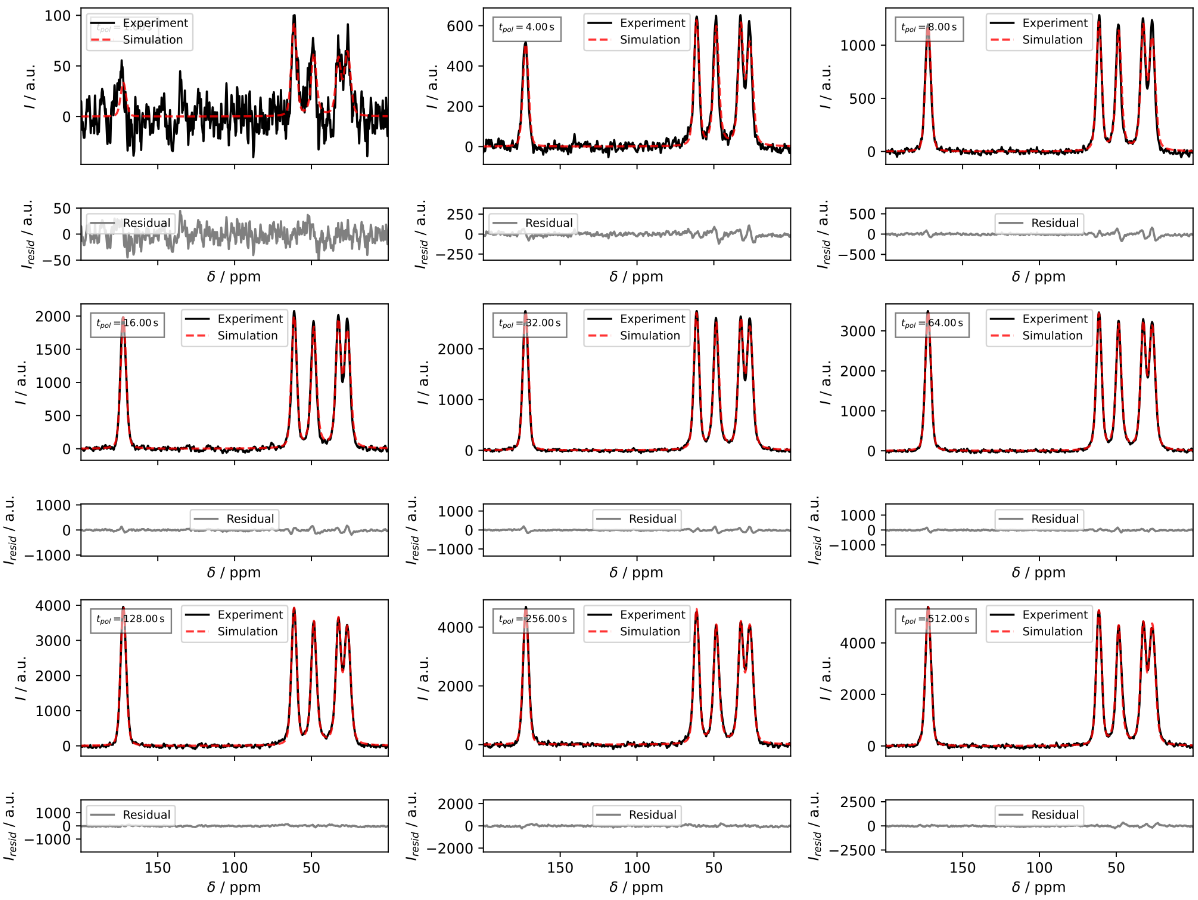

Figure S3.2.5: Number of scan normalized experimental (black) and simulated (red) DNP-enhanced ^{13}C MAS NMR ΔDP_{sat} spectra of 100 mM ^{13}C,^{15}N-L-Pro-Gly (100 mM, 18 kHz MAS and 150 K) at different polarizing times (t_{pol}). Each panel represents a spectrum with increasing t_{pol} from top left to bottom right. The residuals are shown in the lower part of each panel. Each resonance was fitted to an Voigtian lineshape and the line broadening parameter were fitted globally over all polarizing times (t_{pol}). Fitting parameters are given in Table S3.2.5.

S3.2.6. $^{13}C_5$,^{15}N-L-Pro-L-Ala at 100 K

Table S3.2.6: Parameters obtained from spectral simulation/deconvolution of ΔDP_{sat} spectra for $^{13}C_5$,^{15}N-L-Pro-L-Ala at 100 K and 18 kHz MAS frequency. All five resonances were fitted to a Voigtian lineshape, with σ and γ as the Gaussian and Lorentzian line shape parameters, respectively. FWHM parameters were calculated using the previously given formulas (SI S3.2) from the corresponding line broadening parameters. The integrals were obtained by numerical integration of the simulated peaks.

Label	Time / s	Center / ppm	Amplitude / a.u.	σ / ppm	γ / ppm	FWHM Lorentz / ppm	FWHM Gauss / ppm	FWHM Voigt / ppm	Integral / a.u.
CO	1.0	171.476	92.054	1.596	0.476	0.951	3.758	3.203	3622.328
	4.0	171.476	1232.904	1.596	0.476	0.951	3.758	3.203	48514.948
	8.0	171.476	2399.742	1.596	0.476	0.951	3.758	3.203	94430.217
	16.0	171.476	3820.319	1.596	0.476	0.951	3.758	3.203	150330.102
	32.0	171.476	5301.586	1.596	0.476	0.951	3.758	3.203	208618.205
	64.0	171.476	6638.134	1.596	0.476	0.951	3.758	3.203	261211.539
	128.0	171.476	8291.009	1.596	0.476	0.951	3.758	3.203	326252.443
	256.0	171.476	9833.901	1.596	0.476	0.951	3.758	3.203	386965.451
	512.0	171.476	11229.99	1.596	0.476	0.951	3.758	3.203	441901.755
Cα	1.0	61.022	0.0	1.584	0.68	1.36	3.731	3.44	0.0
	4.0	61.022	1355.965	1.584	0.68	1.36	3.731	3.44	53416.705
	8.0	61.022	2616.753	1.584	0.68	1.36	3.731	3.44	103084.001
	16.0	61.022	3926.22	1.584	0.68	1.36	3.731	3.44	154668.943
	32.0	61.022	5187.152	1.584	0.68	1.36	3.731	3.44	204341.942
	64.0	61.022	6504.429	1.584	0.68	1.36	3.731	3.44	256234.558
	128.0	61.022	7905.555	1.584	0.68	1.36	3.731	3.44	311430.316
	256.0	61.022	9739.803	1.584	0.68	1.36	3.731	3.44	383688.412
	512.0	61.022	11425.307	1.584	0.68	1.36	3.731	3.44	450086.957
Cδ	1.0	48.156	0.0	1.392	1.15	2.299	3.277	3.782	0.0
	4.0	48.156	1374.529	1.392	1.15	2.299	3.277	3.782	53880.796
	8.0	48.156	2588.207	1.392	1.15	2.299	3.277	3.782	101456.279
	16.0	48.156	4029.709	1.392	1.15	2.299	3.277	3.782	157962.381
	32.0	48.156	5284.902	1.392	1.15	2.299	3.277	3.782	207165.263
	64.0	48.156	6631.47	1.392	1.15	2.299	3.277	3.782	259949.964
	128.0	48.156	8073.158	1.392	1.15	2.299	3.277	3.782	316463.344
	256.0	48.156	9347.391	1.392	1.15	2.299	3.277	3.782	366412.605
	512.0	48.156	10874.983	1.392	1.15	2.299	3.277	3.782	426293.345
Cβ	1.0	32.17	0.0	1.515	0.819	1.639	3.568	3.512	0.0
	4.0	32.17	1238.547	1.515	0.819	1.639	3.568	3.512	48566.726
	8.0	32.17	2394.672	1.515	0.819	1.639	3.568	3.512	93901.469
	16.0	32.17	3810.32	1.515	0.819	1.639	3.568	3.512	149412.776
	32.0	32.17	5010.498	1.515	0.819	1.639	3.568	3.512	196474.973
	64.0	32.17	6321.77	1.515	0.819	1.639	3.568	3.512	247893.423
	128.0	32.17	7548.762	1.515	0.819	1.639	3.568	3.512	296007.051
	256.0	32.17	9129.076	1.515	0.819	1.639	3.568	3.512	357975.364
	512.0	32.17	10664.428	1.515	0.819	1.639	3.568	3.512	418180.608
Cγ	1.0	26.05	0.015	2.153	0.0	0.0	5.069	3.584	0.585
	4.0	26.05	1190.502	2.153	0.0	0.0	5.069	3.584	47139.182
	8.0	26.05	2247.853	2.153	0.0	0.0	5.069	3.584	89006.096
	16.0	26.05	3480.025	2.153	0.0	0.0	5.069	3.584	137795.228
	32.0	26.05	4698.111	2.153	0.0	0.0	5.069	3.584	186026.633
	64.0	26.05	5774.381	2.153	0.0	0.0	5.069	3.584	228642.697
	128.0	26.05	7101.284	2.153	0.0	0.0	5.069	3.584	281182.806
	256.0	26.05	8162.037	2.153	0.0	0.0	5.069	3.584	323184.431
	512.0	26.05	9173.867	2.153	0.0	0.0	5.069	3.584	363248.909

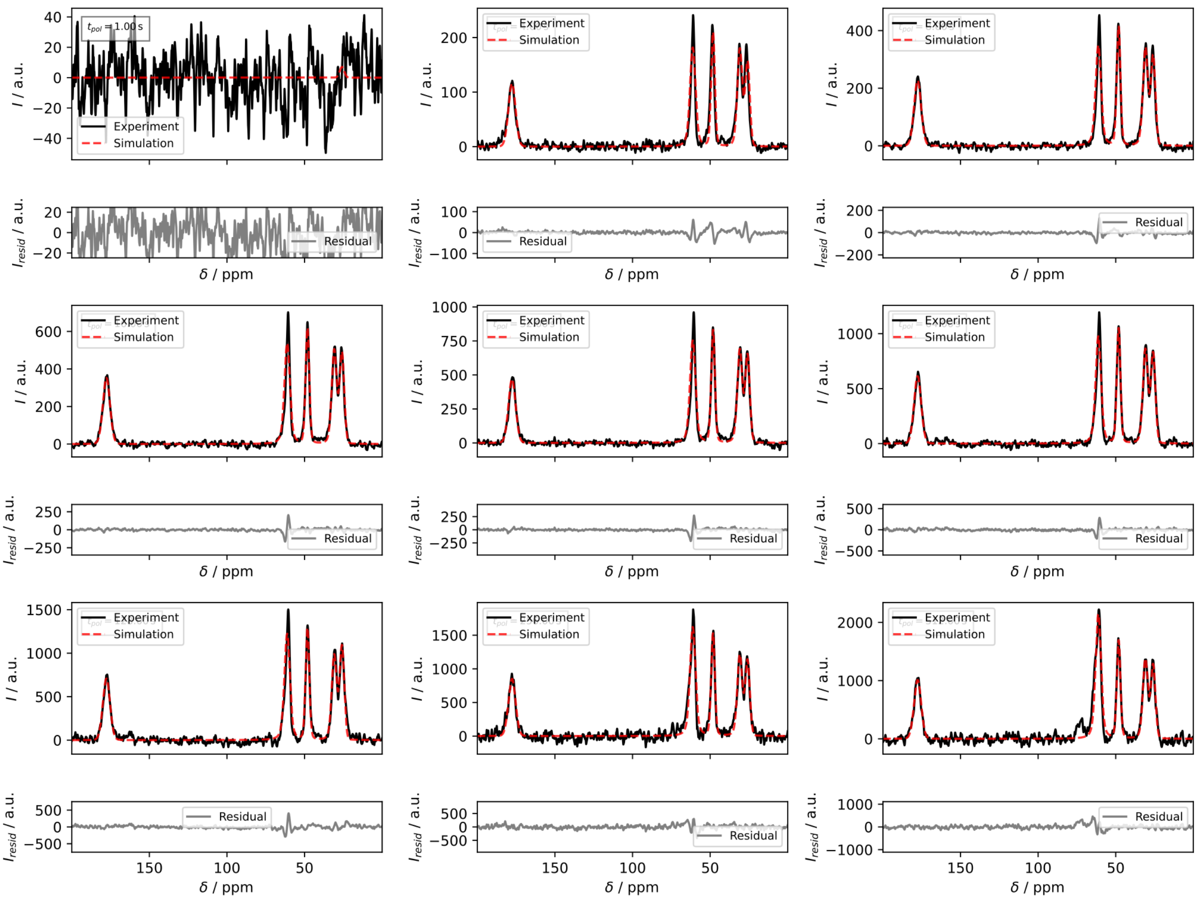

Figure S3.2.6: Number of scan normalized experimental (black) and simulated (red) DNP-enhanced ^{13}C MAS NMR ΔDP_{sat} spectra of 100 mM ^{13}C,^{15}N-L-Pro-L-Ala (100 mM, 18 kHz MAS and 100 K) at different polarizing times (t_{pol}). Each panel represents a spectrum with increasing t_{pol} from top left to bottom right. The residuals are shown in the lower part of each panel. Each resonance was fitted to an Voigtian lineshape and the line broadening parameter were fitted globally over all polarizing times (t_{pol}). Fitting parameters are given in Table S3.2.6.

S3.2.7. $^{13}C_5$,^{15}N-L-Pro-L-Ala at 150 K

Table S3.2.7: Parameters obtained from spectral simulation/deconvolution of ΔDP_{sat} spectra for $^{13}C_5$,^{15}N-L-Pro-L-Ala at 150 K and 18 kHz MAS frequency. All five resonances were fitted to a Voigtian lineshape, with σ and γ as the Gaussian and Lorentzian line shape parameters, respectively. FWHM parameters were calculated using the previously given formulas (SI S3.2) from the corresponding line broadening parameters. The integrals were obtained by numerical integration of the simulated peaks.

Label	Time / s	Center / ppm	Amplitude / a.u.	σ / ppm	γ / ppm	FWHM Lorentz / ppm	FWHM Gauss / ppm	FWHM Voigt / ppm	Integral / a.u.
**CO**	1.0	171.363	105.838	1.597	0.477	0.954	3.761	3.206	4164.725
	4.0	171.363	1231.14	1.597	0.477	0.954	3.761	3.206	48445.48
	8.0	171.363	2402.195	1.597	0.477	0.954	3.761	3.206	94526.645
	16.0	171.363	3835.808	1.597	0.477	0.954	3.761	3.206	150939.457
	32.0	171.363	5303.652	1.597	0.477	0.954	3.761	3.206	208699.284
	64.0	171.363	6655.145	1.597	0.477	0.954	3.761	3.206	261880.657
	128.0	171.363	8227.989	1.597	0.477	0.954	3.761	3.206	323772.249
	256.0	171.363	9886.598	1.597	0.477	0.954	3.761	3.206	389038.684
	512.0	171.363	11253.236	1.597	0.477	0.954	3.761	3.206	442816.051
**Cα**	1.0	60.887	104.112	1.832	0.21	0.419	4.313	3.28	4115.929
	4.0	60.887	1239.466	1.832	0.21	0.419	4.313	3.28	49000.602
	8.0	60.887	2379.492	1.832	0.21	0.419	4.313	3.28	94069.979
	16.0	60.887	3598.487	1.832	0.21	0.419	4.313	3.28	142261.305
	32.0	60.887	4778.059	1.832	0.21	0.419	4.313	3.28	188894.069
	64.0	60.887	5938.871	1.832	0.21	0.419	4.313	3.28	234785.218
	128.0	60.887	7232.778	1.832	0.21	0.419	4.313	3.28	285938.075
	256.0	60.887	8761.639	1.832	0.21	0.419	4.313	3.28	346379.506
	512.0	60.887	10245.271	1.832	0.21	0.419	4.313	3.28	405032.895
**Cδ**	1.0	48.035	108.642	1.297	1.295	2.59	3.053	3.858	4253.188
	4.0	48.035	1413.33	1.297	1.295	2.59	3.053	3.858	55329.831
	8.0	48.035	2663.403	1.297	1.295	2.59	3.053	3.858	104268.383
	16.0	48.035	4162.591	1.297	1.295	2.59	3.053	3.858	162959.439
	32.0	48.035	5444.991	1.297	1.295	2.59	3.053	3.858	213163.534
	64.0	48.035	6839.579	1.297	1.295	2.59	3.053	3.858	267759.659
	128.0	48.035	8266.419	1.297	1.295	2.59	3.053	3.858	323618.359
	256.0	48.035	9594.507	1.297	1.295	2.59	3.053	3.858	375611.125
	512.0	48.035	11120.846	1.297	1.295	2.59	3.053	3.858	435365.081
**Cβ**	1.0	32.069	156.934	1.733	0.372	0.745	4.08	3.304	6186.549
	4.0	32.069	1063.022	1.733	0.372	0.745	4.08	3.304	41905.753
	8.0	32.069	2059.151	1.733	0.372	0.745	4.08	3.304	81174.482
	16.0	32.069	3346.963	1.733	0.372	0.745	4.08	3.304	131941.727
	32.0	32.069	4418.211	1.733	0.372	0.745	4.08	3.304	174171.754
	64.0	32.069	5539.355	1.733	0.372	0.745	4.08	3.304	218368.769
	128.0	32.069	6613.621	1.733	0.372	0.745	4.08	3.304	260717.738
	256.0	32.069	7844.698	1.733	0.372	0.745	4.08	3.304	309248.457
	512.0	32.069	9198.071	1.733	0.372	0.745	4.08	3.304	362600.214
**Cγ**	1.0	25.955	165.03	1.845	0.676	1.352	4.345	3.859	6471.984
	4.0	25.955	1420.375	1.845	0.676	1.352	4.345	3.859	55702.927
	8.0	25.955	2690.926	1.845	0.676	1.352	4.345	3.859	105530.193
	16.0	25.955	4060.335	1.845	0.676	1.352	4.345	3.859	159234.35
	32.0	25.955	5395.241	1.845	0.676	1.352	4.345	3.859	211585.444
	64.0	25.955	6754.473	1.845	0.676	1.352	4.345	3.859	264890.525
	128.0	25.955	8067.168	1.845	0.676	1.352	4.345	3.859	316370.517
	256.0	25.955	9836.692	1.845	0.676	1.352	4.345	3.859	385766.029
	512.0	25.955	10936.384	1.845	0.676	1.352	4.345	3.859	428892.726

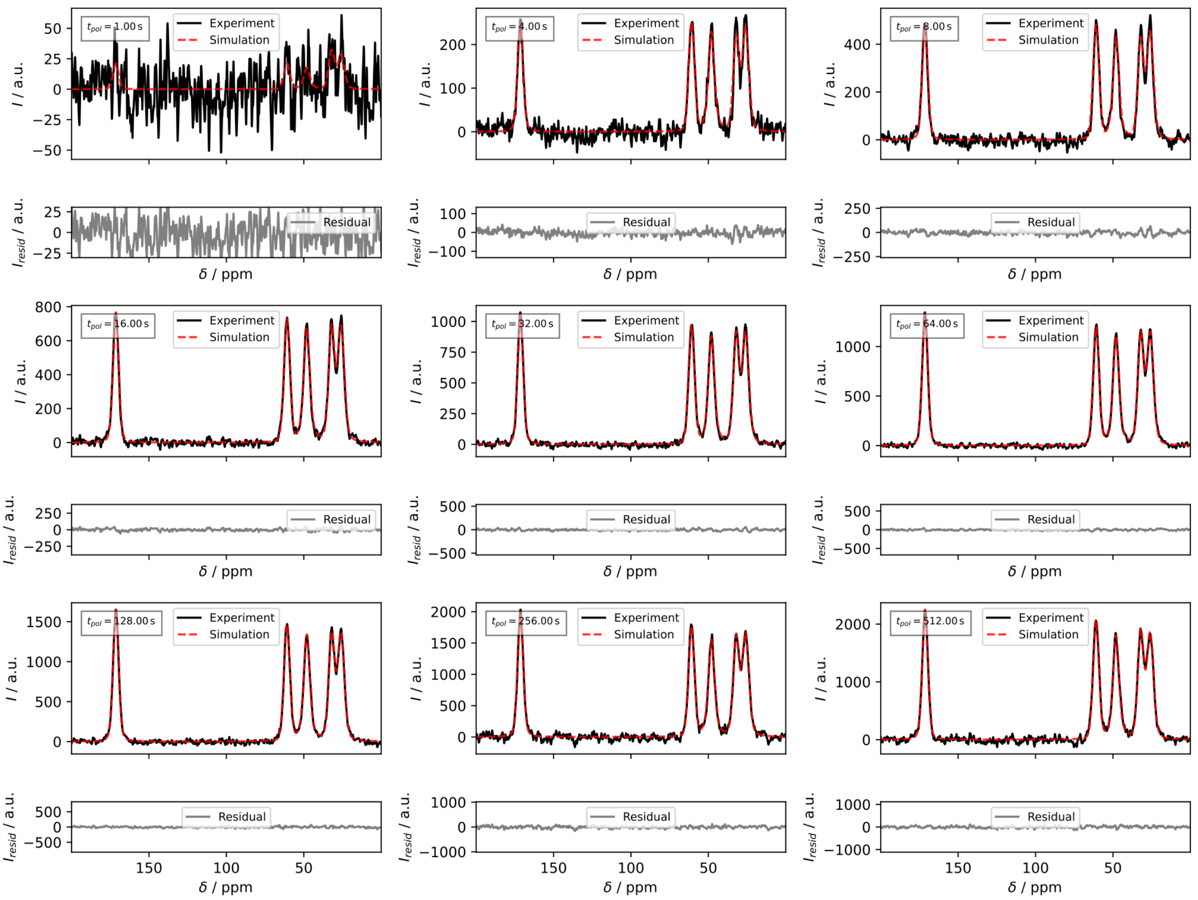

Figure S3.2.7: Number of scan normalized experimental (black) and simulated (red) DNP-enhanced ^{13}C MAS NMR ΔDP_{sat} spectra of 100 mM ^{13}C,^{15}N-L-Pro-L-Ala (100 mM, 18 kHz MAS and 150 K) at different polarizing times (t_{pol}). Each panel represents a spectrum with increasing t_{pol} from top left to bottom right. The residuals are shown in the lower part of each panel. Each resonance was fitted to an Voigtian lineshape and the line broadening parameter were fitted globally over all polarizing times (t_{pol}). Fitting parameters are given in Table S3.2.7.

S3.2.8. Gly-$^{13}C_5$,^{15}N-L-Pro at 100 K

Table S3.2.8: Parameters obtained from spectral simulation/deconvolution of ΔDP$_{sat}$ spectra for Gly-$^{13}C_5$,^{15}N-L-Pro at 100 K and 18 kHz MAS frequency. All five resonances were fitted to a Voigtian lineshape, with σ and γ as the Gaussian and Lorentzian line shape parameters, respectively. FWHM parameters were calculated using the previously given formulas (SI S3.2) from the corresponding line broadening parameters. The integrals were obtained by numerical integration of the simulated peaks.

Label	Time / s	Center / ppm	Amplitude / a.u.	σ / ppm	γ / ppm	FWHM Lorentz / ppm	FWHM Gauss / ppm	FWHM Voigt / ppm	Integral / a.u.
**CO**	1.0	177.385	163.849	2.465	0.0	0.0	5.805	4.105	6487.71
	4.0	177.385	319.007	2.465	0.0	0.0	5.805	4.105	12631.291
	8.0	177.385	759.124	2.465	0.0	0.0	5.805	4.105	30057.982
	16.0	177.385	1204.139	2.465	0.0	0.0	5.805	4.105	47678.618
	32.0	177.385	1930.873	2.465	0.0	0.0	5.805	4.105	76454.109
	64.0	177.385	2620.629	2.465	0.0	0.0	5.805	4.105	103765.425
	128.0	177.385	3084.225	2.465	0.0	0.0	5.805	4.105	122121.792
	256.0	177.385	3963.333	2.465	0.0	0.0	5.805	4.105	156930.63
	512.0	177.385	5173.485	2.465	0.0	0.0	5.805	4.105	204847.336
**Cα**	1.0	61.15	8.066	0.72	0.916	1.833	1.695	2.451	317.194
	4.0	61.15	363.846	0.72	0.916	1.833	1.695	2.451	14307.733
	8.0	61.15	930.505	0.72	0.916	1.833	1.695	2.451	36590.747
	16.0	61.15	1566.717	0.72	0.916	1.833	1.695	2.451	61608.885
	32.0	61.15	2273.349	0.72	0.916	1.833	1.695	2.451	89396.137
	64.0	61.15	3025.528	0.72	0.916	1.833	1.695	2.451	118974.517
	128.0	61.15	4072.932	0.72	0.916	1.833	1.695	2.451	160162.158
	256.0	61.15	5573.133	0.72	0.916	1.833	1.695	2.451	219155.385
	512.0	61.15	7715.299	0.72	0.916	1.833	1.695	2.451	303392.936
**Cδ**	1.0	49.064	2.335	1.279	0.0	0.0	3.012	2.13	92.442
	4.0	49.064	303.651	1.279	0.0	0.0	3.012	2.13	12023.244
	8.0	49.064	753.602	1.279	0.0	0.0	3.012	2.13	29839.342
	16.0	49.064	1204.857	1.279	0.0	0.0	3.012	2.13	47707.06
	32.0	49.064	1795.101	1.279	0.0	0.0	3.012	2.13	71078.134
	64.0	49.064	2265.769	1.279	0.0	0.0	3.012	2.13	89714.528
	128.0	49.064	2879.203	1.279	0.0	0.0	3.012	2.13	114003.832
	256.0	49.064	3956.95	1.279	0.0	0.0	3.012	2.13	156677.89
	512.0	49.064	4514.58	1.279	0.0	0.0	3.012	2.13	178757.611
**Cβ**	1.0	30.791	59.077	1.286	0.658	1.316	3.027	2.93	2320.361
	4.0	30.791	363.278	1.286	0.658	1.316	3.027	2.93	14268.366
	8.0	30.791	984.203	1.286	0.658	1.316	3.027	2.93	38656.289
	16.0	30.791	1571.163	1.286	0.658	1.316	3.027	2.93	61710.204
	32.0	30.791	2311.928	1.286	0.658	1.316	3.027	2.93	90805.055
	64.0	30.791	2907.382	1.286	0.658	1.316	3.027	2.93	114192.564
	128.0	30.791	3992.357	1.286	0.658	1.316	3.027	2.93	156806.867
	256.0	30.791	4651.22	1.286	0.658	1.316	3.027	2.93	182684.855
	512.0	30.791	5986.786	1.286	0.658	1.316	3.027	2.93	235141.594
**Cγ**	1.0	26.51	63.378	1.386	0.227	0.453	3.264	2.56	2501.598
	4.0	26.51	440.895	1.386	0.227	0.453	3.264	2.56	17402.597
	8.0	26.51	621.058	1.386	0.227	0.453	3.264	2.56	24513.822
	16.0	26.51	1101.156	1.386	0.227	0.453	3.264	2.56	43463.806
	32.0	26.51	1584.042	1.386	0.227	0.453	3.264	2.56	62523.801
	64.0	26.51	1788.388	1.386	0.227	0.453	3.264	2.56	70589.566
	128.0	26.51	2715.689	1.386	0.227	0.453	3.264	2.56	107191.118
	256.0	26.51	3360.834	1.386	0.227	0.453	3.264	2.56	132655.676
	512.0	26.51	4702.851	1.386	0.227	0.453	3.264	2.56	185626.504

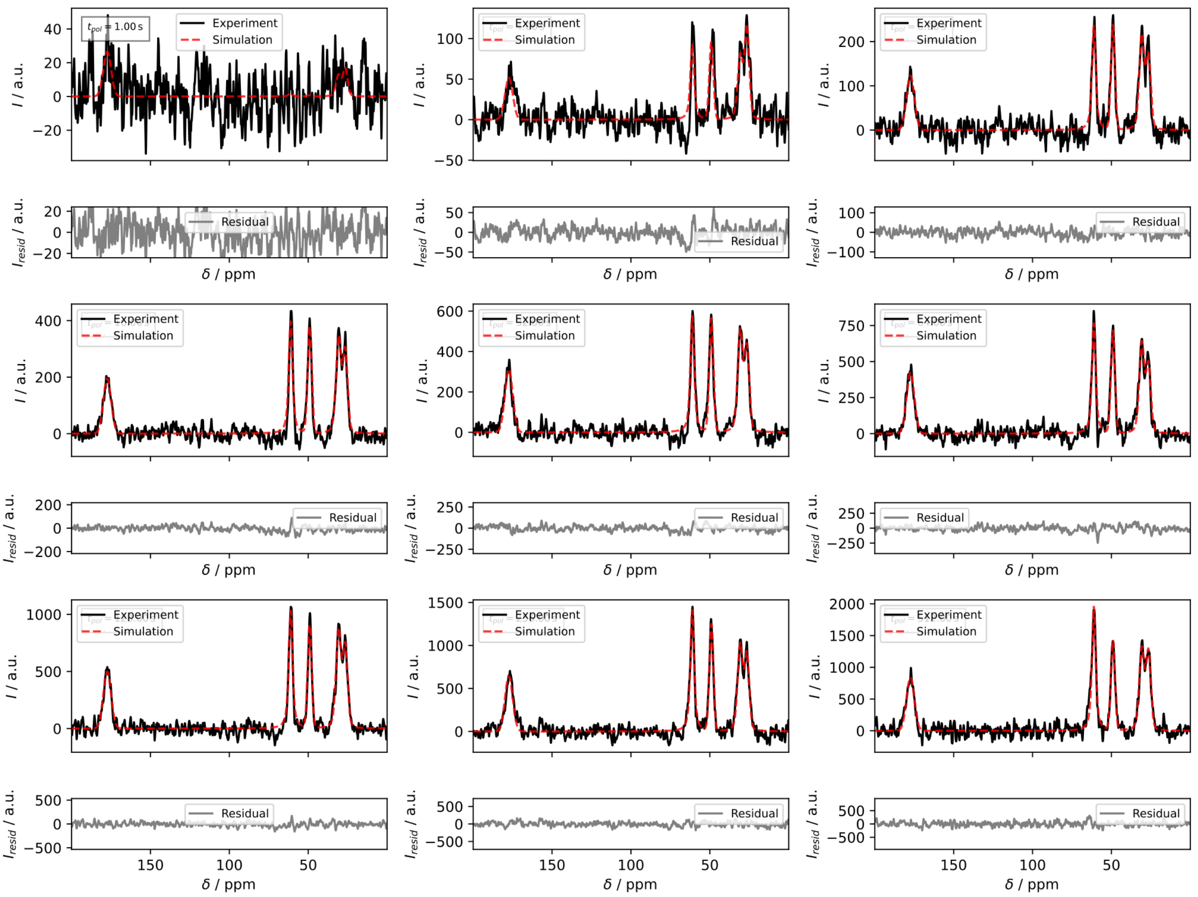

Figure S3.2.8: Number of scan normalized experimental (black) and simulated (red) DNP-enhanced ^{13}C MAS NMR ΔDP_{sat} spectra of 100 mM Gly-^{13}C,^{15}N-L-Pro (100 mM, 18 kHz MAS and 100 K) at different polarizing times (t_{pol}). Each panel represents a spectrum with increasing t_{pol} from top left to bottom right. The residuals are shown in the lower part of each panel. Each resonance was fitted to an Voigtian lineshape and the line broadening parameter were fitted globally over all polarizing times (t_{pol}). Fitting parameters are given in Table S3.2.8.

S3.2.9. Gly-$^{13}C_5$,^{15}N-L-Pro at 150 K

Table S3.2.9: Parameters obtained from spectral simulation/deconvolution of ΔDP_{sat} spectra for Gly-$^{13}C_5$,^{15}N-L-Pro at 150 K and 18 kHz MAS frequency. All five resonances were fitted to a Voigtian lineshape, with σ and γ as the Gaussian and Lorentzian line shape parameters, respectively. FWHM parameters were calculated using the previously given formulas (SI S3.2) from the corresponding line broadening parameters. The integrals were obtained by numerical integration of the simulated peaks.

Label	Time / s	Center / ppm	Amplitude / a.u.	σ / ppm	γ / ppm	FWHM Lorentz / ppm	FWHM Gauss / ppm	FWHM Voigt / ppm	Integral / a.u.
**CO**	1.0	177.357	157.25	1.971	0.661	1.322	4.641	4.046	6160.671
	4.0	177.357	1977.64	1.971	0.661	1.322	4.641	4.046	77479.207
	8.0	177.357	4016.418	1.971	0.661	1.322	4.641	4.046	157353.625
	16.0	177.357	5699.953	1.971	0.661	1.322	4.641	4.046	223310.519
	32.0	177.357	7313.798	1.971	0.661	1.322	4.641	4.046	286537.103
	64.0	177.357	8911.101	1.971	0.661	1.322	4.641	4.046	349115.599
	128.0	177.357	10559.277	1.971	0.661	1.322	4.641	4.046	413687.18
	256.0	177.357	12005.111	1.971	0.661	1.322	4.641	4.046	470331.483
	512.0	177.357	12951.19	1.971	0.661	1.322	4.641	4.046	507396.604
**Glycerol**	1.0	72.954	59.028	1.774	0.0	0.0	4.178	2.954	2337.244
	4.0	72.954	2.819	1.774	0.0	0.0	4.178	2.954	111.616
	8.0	72.954	0.0	1.774	0.0	0.0	4.178	2.954	0.0
	16.0	72.954	0.0	1.774	0.0	0.0	4.178	2.954	0.0
	32.0	72.954	364.735	1.774	0.0	0.0	4.178	2.954	14441.905
	64.0	72.954	342.867	1.774	0.0	0.0	4.178	2.954	13576.027
	128.0	72.954	1158.864	1.774	0.0	0.0	4.178	2.954	45885.922
	256.0	72.954	1446.92	1.774	0.0	0.0	4.178	2.954	57291.664
	512.0	72.954	2294.951	1.774	0.0	0.0	4.178	2.954	90869.938
**Cα**	1.0	61.417	272.952	1.877	0.0	0.0	4.419	3.125	10807.698
	4.0	61.417	2008.084	1.877	0.0	0.0	4.419	3.125	79511.291
	8.0	61.417	3767.668	1.877	0.0	0.0	4.419	3.125	149183.071
	16.0	61.417	5642.292	1.877	0.0	0.0	4.419	3.125	223409.898
	32.0	61.417	7867.293	1.877	0.0	0.0	4.419	3.125	311510.164
	64.0	61.417	9867.901	1.877	0.0	0.0	4.419	3.125	390725.42
	128.0	61.417	12929.771	1.877	0.0	0.0	4.419	3.125	511961.988
	256.0	61.417	15606.694	1.877	0.0	0.0	4.419	3.125	617956.364
	512.0	61.417	18729.419	1.877	0.0	0.0	4.419	3.125	741602.5
**Cδ**	1.0	48.915	227.643	0.901	0.6	1.2	2.122	2.242	8967.073
	4.0	48.915	1911.153	0.901	0.6	1.2	2.122	2.242	75282.075
	8.0	48.915	3589.803	0.901	0.6	1.2	2.122	2.242	141405.615
	16.0	48.915	5140.149	0.901	0.6	1.2	2.122	2.242	202475.149
	32.0	48.915	6581.365	0.901	0.6	1.2	2.122	2.242	259245.972
	64.0	48.915	8036.048	0.901	0.6	1.2	2.122	2.242	316547.249
	128.0	48.915	9218.229	0.901	0.6	1.2	2.122	2.242	363114.418
	256.0	48.915	10395.773	0.901	0.6	1.2	2.122	2.242	409498.959
	512.0	48.915	11818.047	0.901	0.6	1.2	2.122	2.242	465523.637
**Cβ**	1.0	30.702	327.067	1.139	0.523	1.046	2.682	2.518	12867.303
	4.0	30.702	1805.466	1.139	0.523	1.046	2.682	2.518	71029.689
	8.0	30.702	3034.979	1.139	0.523	1.046	2.682	2.518	119400.577
	16.0	30.702	4666.9	1.139	0.523	1.046	2.682	2.518	183602.723
	32.0	30.702	6307.969	1.139	0.523	1.046	2.682	2.518	248164.824
	64.0	30.702	6853.732	1.139	0.523	1.046	2.682	2.518	269635.955
	128.0	30.702	8902.265	1.139	0.523	1.046	2.682	2.518	350228.242
	256.0	30.702	9446.461	1.139	0.523	1.046	2.682	2.518	371637.733
	512.0	30.702	10418.948	1.139	0.523	1.046	2.682	2.518	409896.812
**Cγ**	1.0	26.605	295.375	1.005	1.1	2.2	2.366	3.137	11517.78
	4.0	26.605	2368.621	1.005	1.1	2.2	2.366	3.137	92361.52
	8.0	26.605	4570.784	1.005	1.1	2.2	2.366	3.137	178232.182
	16.0	26.605	5995.132	1.005	1.1	2.2	2.366	3.137	233772.924
	32.0	26.605	7428.177	1.005	1.1	2.2	2.366	3.137	289652.79

64.0	26.605	10069.55	1.005	1.1	2.2	2.366	3.137	392649.914
128.0	26.605	10634.088	1.005	1.1	2.2	2.366	3.137	414663.403
256.0	26.605	12507.845	1.005	1.1	2.2	2.366	3.137	487728.283
512.0	26.605	13905.261	1.005	1.1	2.2	2.366	3.137	542218.828

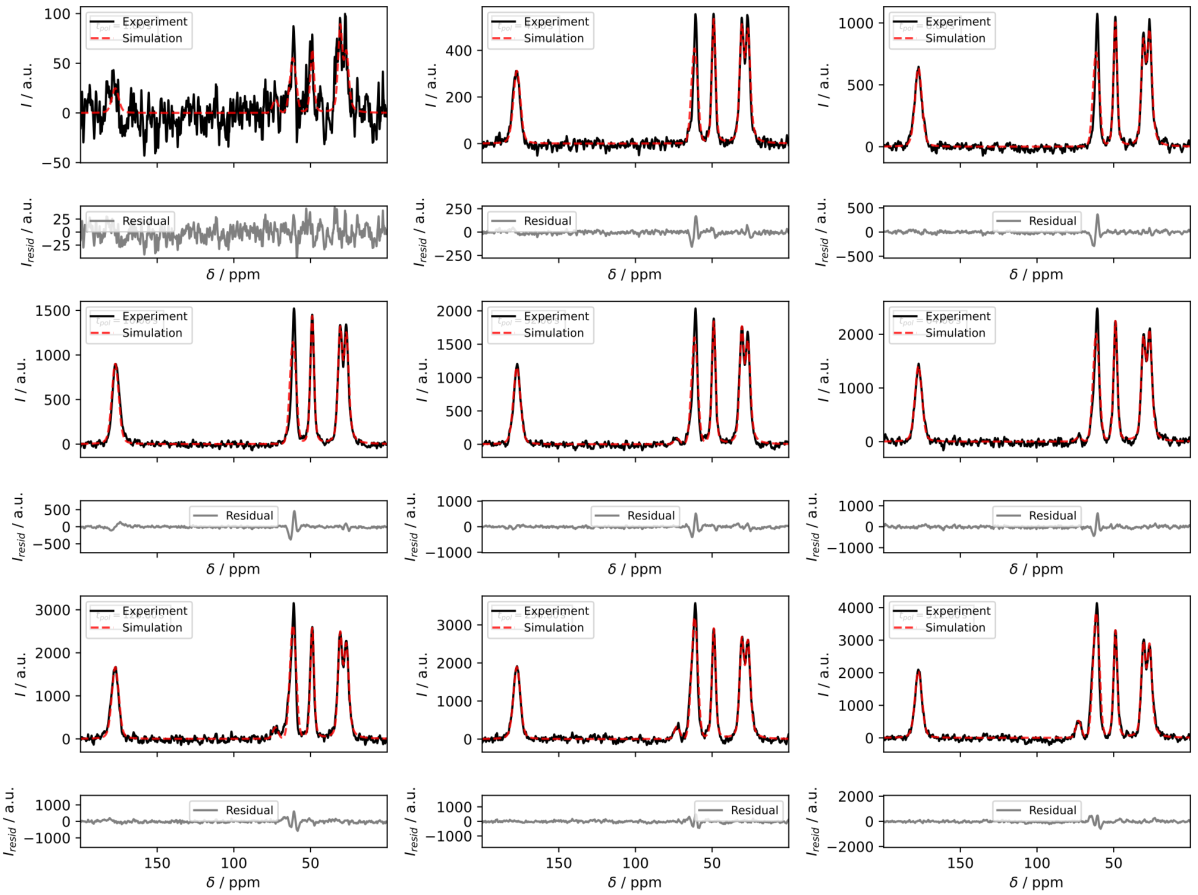

Figure S3.2.9: Number of scan normalized experimental (black) and simulated (red) DNP-enhanced ^{13}C MAS NMR ΔDP_{sat} spectra of 100 mM Gly-^{13}C,^{15}N-L-Pro (100 mM, 18 kHz MAS and 150 K) at different polarizing times (t_{pol}). Each panel represents a spectrum with increasing t_{pol} from top left to bottom right. The residuals are shown in the lower part of each panel. Each resonance was fitted to an Voigtian lineshape and the line broadening parameter were fitted globally over all polarizing times (t_{pol}). Fitting parameters are given in Table S3.2.9.

S3.2.10. L-Ala-$^{13}C_5$,^{15}N-L-Pro at 100 K

Table S3.2.10: Parameters obtained from spectral simulation/deconvolution of ΔDP_{sat} spectra for L-Ala-$^{13}C_5$,^{15}N-L-Pro at 100 K and 18 kHz MAS frequency. All five resonances were fitted to a Voigtian lineshape, with σ and γ as the Gaussian and Lorentzian line shape parameters, respectively. FWHM parameters were calculated using the previously given formulas (SI S3.2) from the corresponding line broadening parameters. The integrals were obtained by numerical integration of the simulated peaks.

Label	Time / s	Center / ppm	Amplitude / a.u.	σ / ppm	γ / ppm	FWHM Lorentz / ppm	FWHM Gauss / ppm	FWHM Voigt / ppm	Integral / a.u.
CO	1.0	178.236	146.824	2.184	0.35	0.7	5.144	4.026	5779.885
	4.0	178.236	130.164	2.184	0.35	0.7	5.144	4.026	5124.06
	8.0	178.236	263.927	2.184	0.35	0.7	5.144	4.026	10389.78
	16.0	178.236	506.8	2.184	0.35	0.7	5.144	4.026	19950.736
	32.0	178.236	848.623	2.184	0.35	0.7	5.144	4.026	33406.993
	64.0	178.236	1191.881	2.184	0.35	0.7	5.144	4.026	46919.732
	128.0	178.236	1603.171	2.184	0.35	0.7	5.144	4.026	63110.617
	256.0	178.236	1903.729	2.184	0.35	0.7	5.144	4.026	74942.391
	512.0	178.236	2674.043	2.184	0.35	0.7	5.144	4.026	105266.685
Cα	1.0	60.829	0.0	1.19	0.0	0.0	2.803	1.982	0.0
	4.0	60.829	190.297	1.19	0.0	0.0	2.803	1.982	7534.932
	8.0	60.829	402.064	1.19	0.0	0.0	2.803	1.982	15919.993
	16.0	60.829	670.174	1.19	0.0	0.0	2.803	1.982	26535.949
	32.0	60.829	814.743	1.19	0.0	0.0	2.803	1.982	32260.26
	64.0	60.829	1130.914	1.19	0.0	0.0	2.803	1.982	44779.262
	128.0	60.829	1518.319	1.19	0.0	0.0	2.803	1.982	60118.81
	256.0	60.829	2196.215	1.19	0.0	0.0	2.803	1.982	86960.528
	512.0	60.829	2754.987	1.19	0.0	0.0	2.803	1.982	109085.47
Cδ	1.0	48.086	31.579	0.834	0.603	1.206	1.964	2.143	1243.83
	4.0	48.086	269.038	0.834	0.603	1.206	1.964	2.143	10596.714
	8.0	48.086	488.455	0.834	0.603	1.206	1.964	2.143	19238.962
	16.0	48.086	781.255	0.834	0.603	1.206	1.964	2.143	30771.607
	32.0	48.086	1080.221	0.834	0.603	1.206	1.964	2.143	42547.09
	64.0	48.086	1377.265	0.834	0.603	1.206	1.964	2.143	54246.91
	128.0	48.086	1739.352	0.834	0.603	1.206	1.964	2.143	68508.547
	256.0	48.086	2367.882	0.834	0.603	1.206	1.964	2.143	93264.732
	512.0	48.086	2666.841	0.834	0.603	1.206	1.964	2.143	105039.932
Cβ	1.0	30.819	0.0	0.918	0.871	1.742	2.161	2.661	0.0
	4.0	30.819	180.832	0.918	0.871	1.742	2.161	2.661	7083.895
	8.0	30.819	358.98	0.918	0.871	1.742	2.161	2.661	14062.693
	16.0	30.819	731.443	0.918	0.871	1.742	2.161	2.661	28653.559
	32.0	30.819	957.076	0.918	0.871	1.742	2.161	2.661	37492.495
	64.0	30.819	1291.84	0.918	0.871	1.742	2.161	2.661	50606.509
	128.0	30.819	1686.05	0.918	0.871	1.742	2.161	2.661	66049.321
	256.0	30.819	2245.344	0.918	0.871	1.742	2.161	2.661	87959.067
	512.0	30.819	3027.182	0.918	0.871	1.742	2.161	2.661	118586.802
Cγ	1.0	25.796	0.0	1.607	0.0	0.0	3.784	2.676	0.0
	4.0	25.796	180.195	1.607	0.0	0.0	3.784	2.676	7134.931
	8.0	25.796	348.976	1.607	0.0	0.0	3.784	2.676	13817.934
	16.0	25.796	512.718	1.607	0.0	0.0	3.784	2.676	20301.38
	32.0	25.796	801.143	1.607	0.0	0.0	3.784	2.676	31721.772
	64.0	25.796	1187.999	1.607	0.0	0.0	3.784	2.676	47039.593
	128.0	25.796	1499.856	1.607	0.0	0.0	3.784	2.676	59387.758
	256.0	25.796	1919.871	1.607	0.0	0.0	3.784	2.676	76018.493
	512.0	25.796	2223.183	1.607	0.0	0.0	3.784	2.676	88028.35

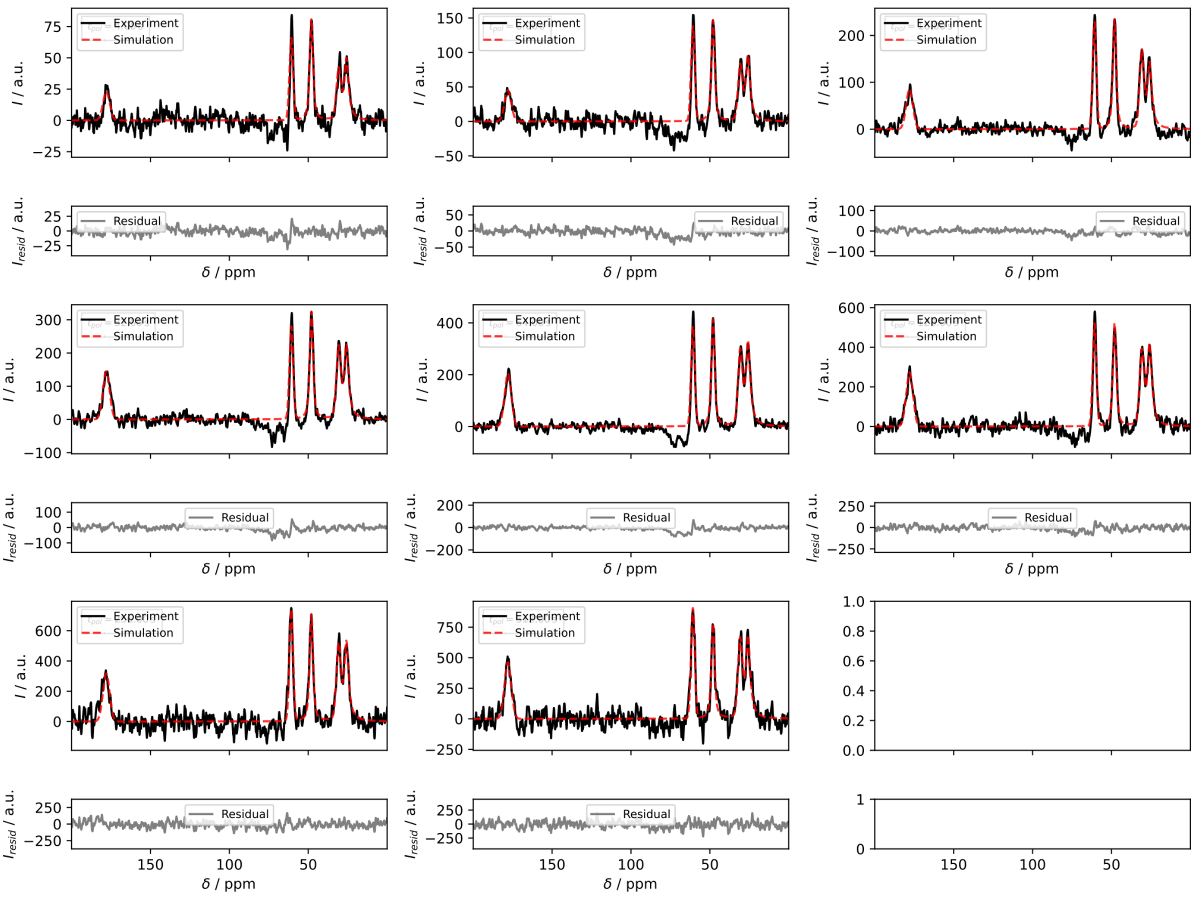

Figure S3.2.10: Number of scan normalized experimental (black) and simulated (red) DNP-enhanced ^{13}C MAS NMR ΔDP_{sat} spectra of 100 mM L-Ala-^{13}C,^{15}N-L-Pro (100 mM, 18 kHz MAS and 100 K) at different polarizing times (t_{pol}). Each panel represents a spectrum with increasing t_{pol} from top left to bottom right. The residuals are shown in the lower part of each panel. Each resonance was fitted to an Voigtian lineshape and the line broadening parameter were fitted globally over all polarizing times (t_{pol}). Fitting parameters are given in Table S3.2.10.

S3.2.11. L-Ala-$^{13}C_5$,^{15}N-L-Pro at 150 K

Table S3.2.11: Parameters obtained from spectral simulation/deconvolution of ΔDP_{sat} spectra for L-Ala-$^{13}C_5$,^{15}N-L-Pro at 150 K and 18 kHz MAS frequency. All five resonances were fitted to a Voigtian lineshape, with σ and γ as the Gaussian and Lorentzian line shape parameters, respectively. FWHM parameters were calculated using the previously given formulas (SI S3.2) from the corresponding line broadening parameters. The integrals were obtained by numerical integration of the simulated peaks.

Label	Time / s	Center / ppm	Amplitude / a.u.	σ / ppm	γ / ppm	FWHM Lorentz / ppm	FWHM Gauss / ppm	FWHM Voigt / ppm	Integral / a.u.
CO	1.0	177.822	161.413	1.562	0.873	1.745	3.678	3.658	6300.808
	4.0	177.822	1115.072	1.562	0.873	1.745	3.678	3.658	43527.274
	8.0	177.822	2093.22	1.562	0.873	1.745	3.678	3.658	81709.682
	16.0	177.822	3238.046	1.562	0.873	1.745	3.678	3.658	126398.418
	32.0	177.822	4226.799	1.562	0.873	1.745	3.678	3.658	164994.798
	64.0	177.822	4920.641	1.562	0.873	1.745	3.678	3.658	192079.196
	128.0	177.822	6003.569	1.562	0.873	1.745	3.678	3.658	234351.727
	256.0	177.822	6877.747	1.562	0.873	1.745	3.678	3.658	268475.626
	512.0	177.822	7882.12	1.562	0.873	1.745	3.678	3.658	307681.723
Cα	1.0	61.503	173.54	0.739	0.949	1.898	1.739	2.528	6822.688
	4.0	61.503	1305.698	0.739	0.949	1.898	1.739	2.528	51333.147
	8.0	61.503	2530.894	0.739	0.949	1.898	1.739	2.528	99501.376
	16.0	61.503	3741.454	0.739	0.949	1.898	1.739	2.528	147094.185
	32.0	61.503	5111.991	0.739	0.949	1.898	1.739	2.528	200976.453
	64.0	61.503	6624.999	0.739	0.949	1.898	1.739	2.528	260459.937
	128.0	61.503	7735.08	0.739	0.949	1.898	1.739	2.528	304102.473
	256.0	61.503	9992.722	0.739	0.949	1.898	1.739	2.528	392861.009
	512.0	61.503	11990.437	0.739	0.949	1.898	1.739	2.528	471400.624
Cδ	1.0	48.698	170.365	0.901	0.43	0.86	2.121	2.012	6720.607
	4.0	48.698	1068.213	0.901	0.43	0.86	2.121	2.012	42139.183
	8.0	48.698	1952.747	0.901	0.43	0.86	2.121	2.012	77032.552
	16.0	48.698	2842.296	0.901	0.43	0.86	2.121	2.012	112123.746
	32.0	48.698	3663.278	0.901	0.43	0.86	2.121	2.012	144510.11
	64.0	48.698	4465.03	0.901	0.43	0.86	2.121	2.012	176137.841
	128.0	48.698	5289.438	0.901	0.43	0.86	2.121	2.012	208659.352
	256.0	48.698	6018.397	0.901	0.43	0.86	2.121	2.012	237415.533
	512.0	48.698	6492.703	0.901	0.43	0.86	2.121	2.012	256126.109
Cβ	1.0	31.0	227.855	0.537	1.615	3.231	1.264	3.476	8844.959
	4.0	31.0	1564.524	0.537	1.615	3.231	1.264	3.476	60732.202
	8.0	31.0	2903.898	0.537	1.615	3.231	1.264	3.476	112724.416
	16.0	31.0	4109.314	0.537	1.615	3.231	1.264	3.476	159516.656
	32.0	31.0	5429.753	0.537	1.615	3.231	1.264	3.476	210773.87
	64.0	31.0	6655.267	0.537	1.615	3.231	1.264	3.476	258346.241
	128.0	31.0	6926.937	0.537	1.615	3.231	1.264	3.476	268892.032
	256.0	31.0	8305.068	0.537	1.615	3.231	1.264	3.476	322388.765
	512.0	31.0	8898.822	0.537	1.615	3.231	1.264	3.476	345437.275
Cγ	1.0	26.333	127.727	1.147	0.281	0.562	2.7	2.227	5037.585
	4.0	26.333	858.659	1.147	0.281	0.562	2.7	2.227	33865.779
	8.0	26.333	1566.103	1.147	0.281	0.562	2.7	2.227	61767.611
	16.0	26.333	2355.755	1.147	0.281	0.562	2.7	2.227	92911.711
	32.0	26.333	2841.525	1.147	0.281	0.562	2.7	2.227	112070.637
	64.0	26.333	3469.662	1.147	0.281	0.562	2.7	2.227	136844.56
	128.0	26.333	4742.462	1.147	0.281	0.562	2.7	2.227	187044.212
	256.0	26.333	4781.317	1.147	0.281	0.562	2.7	2.227	188576.649
	512.0	26.333	5831.024	1.147	0.281	0.562	2.7	2.227	229977.434

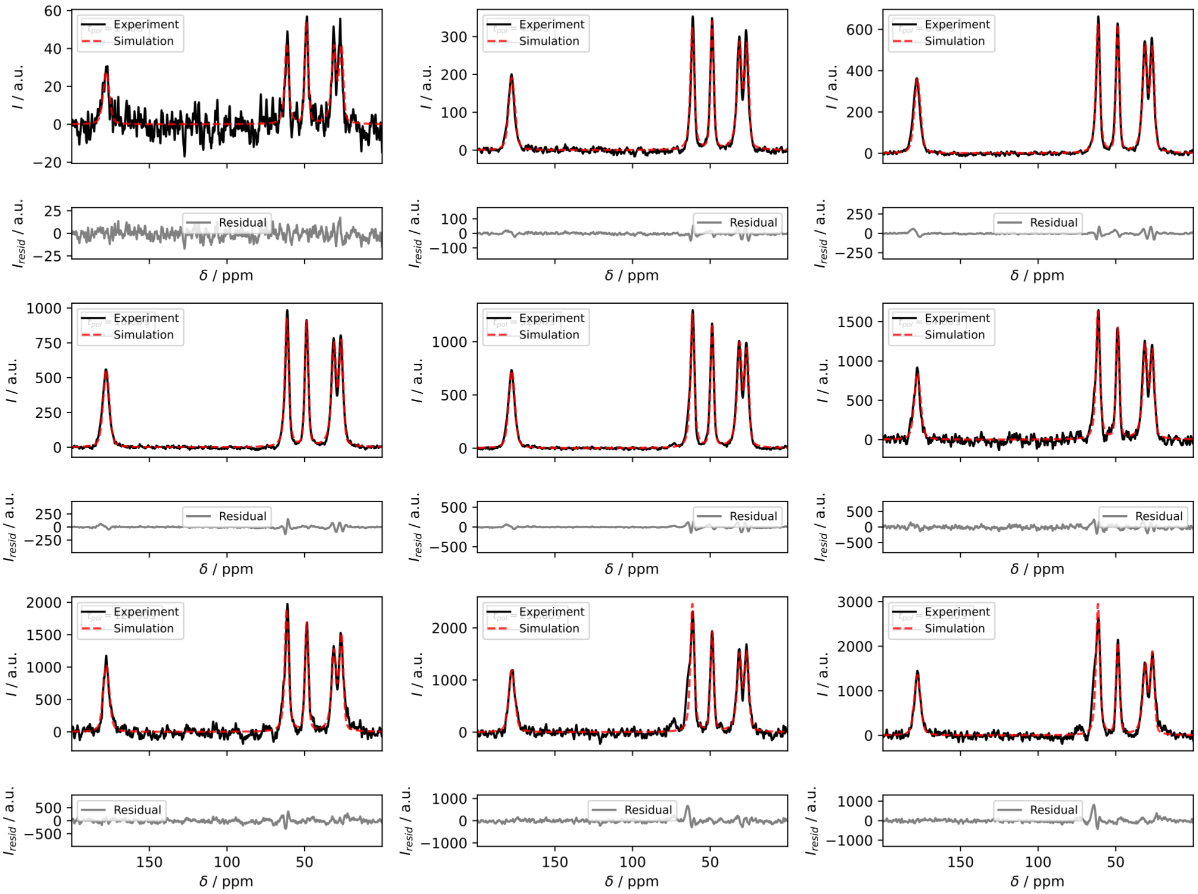

Figure S3.2.11: Number of scan normalized experimental (black) and simulated (red) DNP-enhanced ^{13}C MAS NMR ΔDP_{sat} spectra of 100 mM L-Ala-^{13}C,^{15}N-L-Pro (100 mM, 18 kHz MAS and 150 K) at different polarizing times (t_{pol}). Each panel represents a spectrum with increasing t_{pol} from top left to bottom right. The residuals are shown in the lower part of each panel. Each resonance was fitted to an Voigtian lineshape and the line broadening parameter were fitted globally over all polarizing times (t_{pol}). Fitting parameters are given in Table S3.2.11.

S3.2.12. L-Pro at 100 K

Table S3.2.12: Parameters obtained from spectral simulation/deconvolution of ΔDP_{sat} spectra for L-Pro at 100 K and 18 kHz MAS frequency. All five resonances were fitted to a Voigtian lineshape, with σ and γ as the Gaussian and Lorentzian line shape parameters, respectively. FWHM parameters were calculated using the previously given formulas (SI S3.2) from the corresponding line broadening parameters. The integrals were obtained by numerical integration of the simulated peaks.

Label	Time / s	Center / ppm	Amplitude / a.u.	σ / ppm	γ / ppm	FWHM Lorentz / ppm	FWHM Gauss / ppm	FWHM Voigt / ppm	Integral / a.u.
**Cα**	4.0	60.828	4.975	1.662	0.0	0.0	3.913	2.767	196.981
	8.0	60.828	55.529	1.662	0.0	0.0	3.913	2.767	2198.743
	16.0	60.828	191.732	1.662	0.0	0.0	3.913	2.767	7591.839
	32.0	60.828	115.731	1.662	0.0	0.0	3.913	2.767	4582.49
	64.0	60.828	245.407	1.662	0.0	0.0	3.913	2.767	9717.163
	128.0	60.828	352.032	1.662	0.0	0.0	3.913	2.767	13939.106
	256.0	60.828	619.256	1.662	0.0	0.0	3.913	2.767	24520.123
**Cδ**	4.0	46.295	116.09	1.189	1.097	2.195	2.799	3.401	4551.566
	8.0	46.295	384.972	1.189	1.097	2.195	2.799	3.401	15093.646
	16.0	46.295	713.334	1.189	1.097	2.195	2.799	3.401	27967.77
	32.0	46.295	850.37	1.189	1.097	2.195	2.799	3.401	33340.542
	64.0	46.295	1144.008	1.189	1.097	2.195	2.799	3.401	44853.23
	128.0	46.295	1311.614	1.189	1.097	2.195	2.799	3.401	51424.583
	256.0	46.295	1506.275	1.189	1.097	2.195	2.799	3.401	59056.709
**Cβ**	4.0	30.049	263.535	1.059	0.836	1.672	2.494	2.822	10326.043
	8.0	30.049	369.05	1.059	0.836	1.672	2.494	2.822	14460.437
	16.0	30.049	749.856	1.059	0.836	1.672	2.494	2.822	29381.514
	32.0	30.049	949.416	1.059	0.836	1.672	2.494	2.822	37200.842
	64.0	30.049	1130.365	1.059	0.836	1.672	2.494	2.822	44290.949
	128.0	30.049	1308.015	1.059	0.836	1.672	2.494	2.822	51251.797
	256.0	30.049	1437.338	1.059	0.836	1.672	2.494	2.822	56319.016
**Cγ**	4.0	24.699	305.783	1.973	0.222	0.443	4.645	3.528	12068.153
	8.0	24.699	631.32	1.973	0.222	0.443	4.645	3.528	24915.885
	16.0	24.699	914.624	1.973	0.222	0.443	4.645	3.528	36096.883
	32.0	24.699	1070.448	1.973	0.222	0.443	4.645	3.528	42246.696
	64.0	24.699	1334.755	1.973	0.222	0.443	4.645	3.528	52677.9
	128.0	24.699	1429.878	1.973	0.222	0.443	4.645	3.528	56432.083
	256.0	24.699	1720.151	1.973	0.222	0.443	4.645	3.528	67888.104

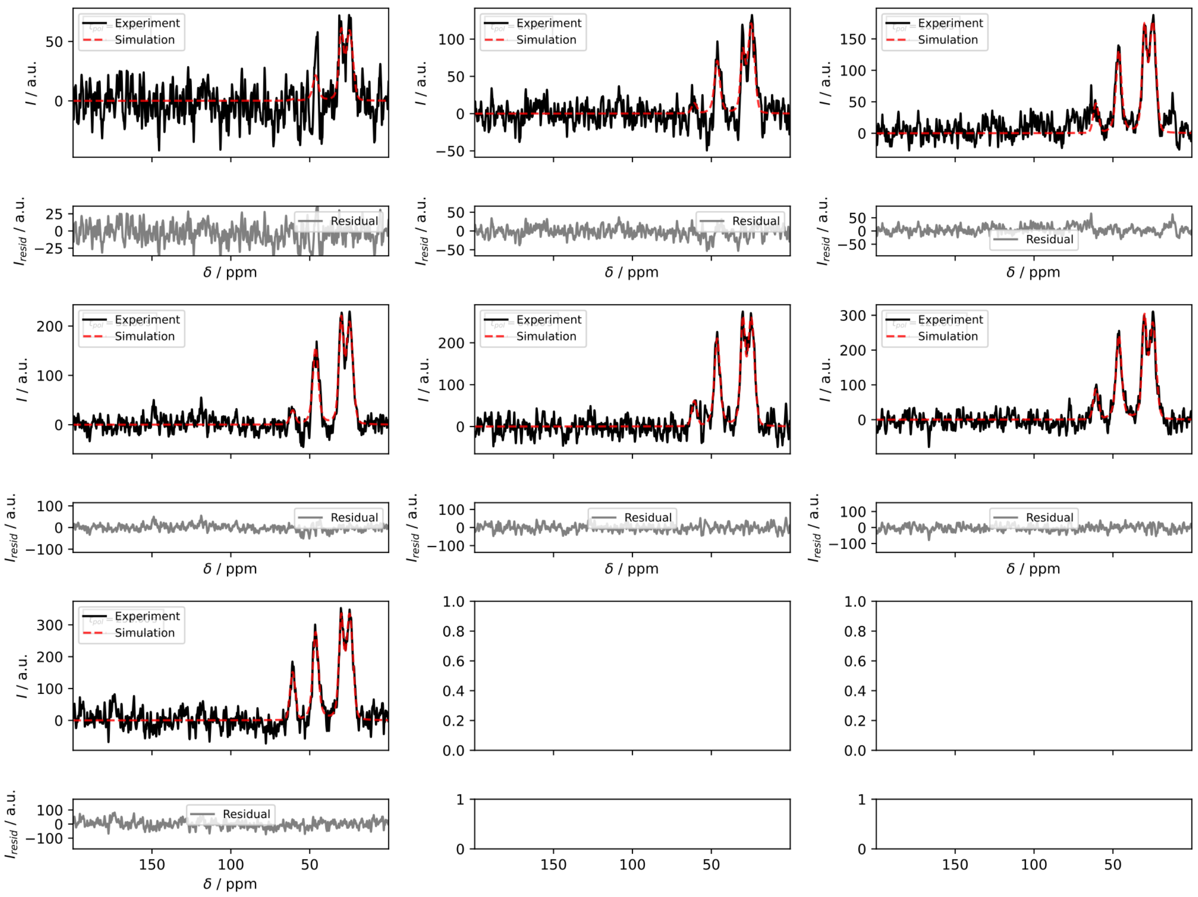

Figure S3.2.12: Number of scan normalized experimental (black) and simulated (red) DNP-enhanced ^{13}C MAS NMR ΔDP_{sat} spectra of 1 M L-Pro (100 mM, 18 kHz MAS and 100 K) at different polarizing times (t_{pol}). Each panel represents a spectrum with increasing t_{pol} from top left to bottom right. The residuals are shown in the lower part of each panel. Each resonance was fitted to an Voigtian lineshape and the line broadening parameter were fitted globally over all polarizing times (t_{pol}). Fitting parameters are given in Table S3.2.12.

S4. Buildup analysis

S4.1. $^{13}C_5$,^{15}N-L-Pro at 100 K

Table S4.1: Polarization build-up parameters for each carbon position in $^{13}C_5$,^{15}N-L-Pro (100 mM) for ΔDP_{sat} experiments at 100 K and 18 kHz MAS frequency. A biexponential fit was necessary for all signals, resulting in four parameters: the time constants of the fast (t_f) and slow (t_s) build-up components, along with their corresponding plateau integrals, A_f and A_s. Additionally, the respective build-up rates are given as $R_f = 1/t_f$ and $R_s = 1/t_s$, as well as the sensitivities $\sigma_f = A_f/\mathrm{sqrt}(t_f)$ and $\sigma_s = A_s/\mathrm{sqrt}(t_s)$.

Label	A_f / a.u.	t_f / s	A_s / a.u.	t_s / s	R_f / 1/s	R_s / 1/s	σ_f / a.u.	σ_s / a.u.
**CO**	511073.448	14.871	897100.69	237.204	0.06724	0.00422	132529.705	58247.888
**Cα**	514135.071	13.577	876808.769	240.665	0.07365	0.00416	139532.482	56519.514
**Cδ**	520867.467	13.375	834280.738	226.764	0.07477	0.00441	142423.068	55401.964
**Cβ**	487546.319	13.587	792733.302	248.545	0.0736	0.00402	132267.789	50283.394
**Cγ**	506332.309	13.023	846581.204	210.647	0.07679	0.00475	140307.252	58329.867
NH	48223.453	521.119			0.00192		211.467	

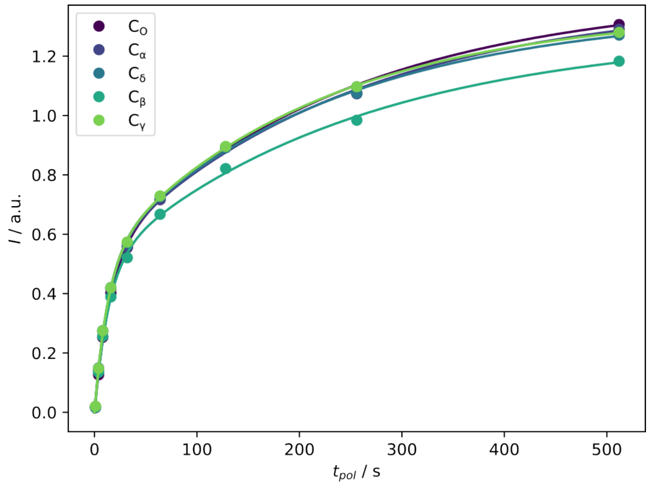

Figure S4.1: Polarization build-up dynamics for each carbon position in $^{13}C_5$,^{15}N-L-Pro (0.1 M) with 10 mM AMUPol in DNP matrix (glycerol-d_8:D_2O:H_2O, 60:30:10 vol.-%) at 100 K and 18 kHz MAS frequency. Solid lines represent biexponential fits with their parameters provided in Table S4.1.

S4.2. $^{13}C_5$,^{15}N-L-Pro at 125 K

Table S4.2: Polarization build-up parameters for each carbon position in $^{13}C_5$,^{15}N-L-Pro (100 mM) for ΔDP_{sat} experiments at 125 K and 18 kHz MAS frequency. A biexponential fit was necessary for all signals, resulting in four parameters: the time constants of the fast (t_f) and slow (t_s) build-up components, along with their corresponding plateau integrals, A_f and A_s. Additionally, the respective build-up rates are given as $R_f = 1/t_f$ and $R_s = 1/t_s$, as well as the sensitivities $\sigma_f = A_f/\mathrm{sqrt}(t_f)$ and $\sigma_s = A_s/\mathrm{sqrt}(t_s)$.

Label	A_f / a.u.	t_f / s	A_s / a.u.	t_s / s	R_f / 1/s	R_s / 1/s	σ_f / a.u.	σ_s / a.u.
**CO**	521721.881	14.625	756210.597	236.666	0.06838	0.00423	136424.105	49155.806
**Cα**	481615.224	12.751	698921.995	189.816	0.07843	0.00527	134873.952	50729.701
**Cδ**	488785.756	12.493	689606.56	197.563	0.08004	0.00506	138288.215	49062.376
**Cβ**	476421.723	12.543	626941.349	190.833	0.07973	0.00524	134521.235	45383.729
**Cγ**	470927.487	12.009	747065.679	195.142	0.08327	0.00512	135894.105	53479.016

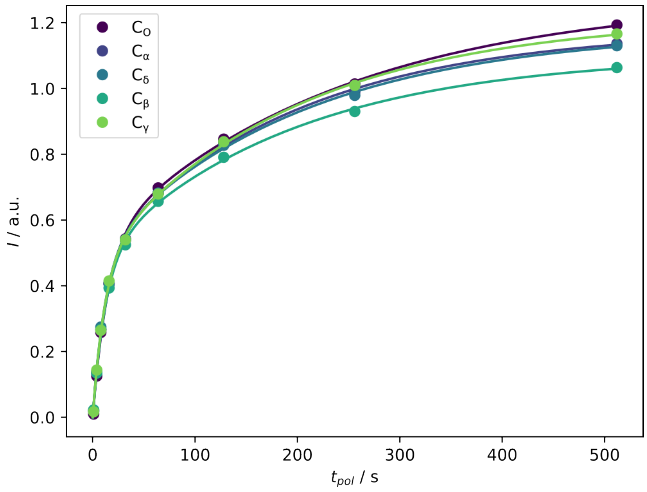

Figure S4.2: Polarization build-up dynamics for each carbon position in $^{13}C_5$,^{15}N-L-Pro (0.1 M) with 10 mM AMUPol in DNP matrix (glycerol-d_8:D_2O:H_2O, 60:30:10 vol.-%) at 125 K and 18 kHz MAS frequency. Solid lines represent biexponential fits with their parameters provided in Table S4.2.

S4.3. $^{13}C_5$,^{15}N-L-Pro at 150 K

Table S4.3: Polarization build-up parameters for each carbon position in $^{13}C_5$,^{15}N-L-Pro (100 mM) for ΔDP_{sat} experiments at 150 K and 18 kHz MAS frequency. A biexponential fit was necessary for all signals, resulting in four parameters: the time constants of the fast (t_f) and slow (t_s) build-up components, along with their corresponding plateau integrals, A_f and A_s. Additionally, the respective build-up rates are given as $R_f = 1/t_f$ and $R_s = 1/t_s$, as well as the sensitivities $\sigma_f = A_f/\text{sqrt}(t_f)$ and $\sigma_s = A_s/\text{sqrt}(t_s)$.

Label	A_f / a.u.	t_f / s	A_s / a.u.	t_s / s	R_f / 1/s	R_s / 1/s	σ_f / a.u.	σ_s / a.u.
**CO**	535992.915	13.356	691704.929	164.167	0.07487	0.00609	146663.094	53985.601
**Cα**	546351.431	12.65	632344.344	170.045	0.07905	0.00588	153612.594	48492.18
**Cδ**	538017.618	12.24	606347.642	159.173	0.0817	0.00628	153782.1	48060.357
**Cβ**	524531.102	12.082	545801.282	149.685	0.08277	0.00668	150904.374	44611.355
**Cγ**	477745.397	11.789	692560.705	146.941	0.08482	0.00681	139141.931	57132.911

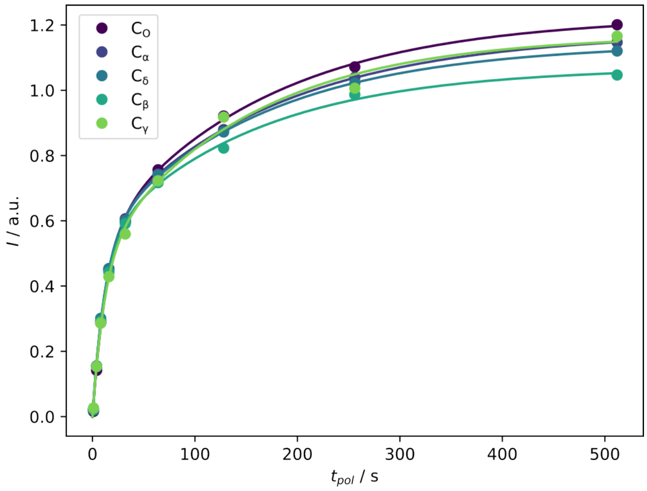

Figure S4.3: Polarization build-up dynamics for each carbon position in $^{13}C_5$,^{15}N-L-Pro (0.1 M) with 10 mM AMUPol in DNP matrix (glycerol-d_8:D_2O:H_2O, 60:30:10 vol.-%) at 150 K and 18 kHz MAS frequency. Solid lines represent biexponential fits with their parameters provided in Table S4.3.

S4.4. $^{13}C_5,^{15}N$-L-Pro-Gly at 100 K

Table S4.4: Polarization build-up parameters for each carbon position in $^{13}C_5,^{15}N$-L-Pro-Gly (100 mM) for ΔDP_{sat} experiments at 100 K and 18 kHz MAS frequency. A biexponential fit was necessary for all signals, resulting in four parameters: the time constants of the fast (t_f) and slow (t_s) build-up components, along with their corresponding plateau integrals, A_f and A_s. Additionally, the respective build-up rates are given as $R_f = 1/t_f$ and $R_s = 1/t_s$, as well as the sensitivities $\sigma_f = A_f/\text{sqrt}(t_f)$ and $\sigma_s = A_s/\text{sqrt}(t_s)$.

Label	A_f / a.u.	t_f / s	A_s / a.u.	t_s / s	R_f / 1/s	R_s / 1/s	σ_f / a.u.	σ_s / a.u.
**CO**	463148.247	14.897	838408.425	239.726	0.06713	0.00417	119997.063	54149.951
**Cα**	458930.643	13.747	834900.882	249.571	0.07274	0.00401	123777.894	52849.132
**Cδ**	490727.133	13.429	841728.583	246.724	0.07447	0.00405	133911.611	53587.855
**Cβ**	440504.438	14.027	749154.5	260.551	0.07129	0.00384	117616.402	46411.438
**Cγ**	493436.989	13.666	869663.825	251.608	0.07317	0.00397	133478.4	54826.331

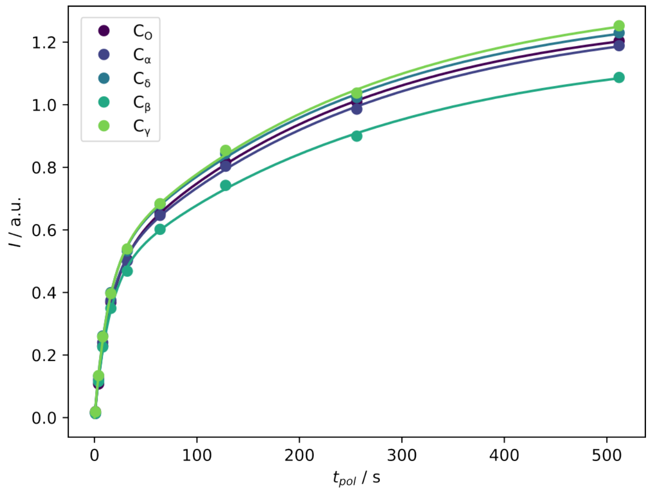

Figure S4.4: Polarization build-up dynamics for each carbon position in $^{13}C_5,^{15}N$-L-Pro-Gly (0.1 M) with 10 mM AMUPol in DNP matrix (glycerol-d_8:D_2O:H_2O, 60:30:10 vol.-%) at 100 K and 18 kHz MAS frequency. Solid lines represent biexponential fits with their parameters provided in Table S4.4.

S4.5. $^{13}C_5,^{15}N$-L-Pro-Gly at 150 K

Table S4.5: Polarization build-up parameters for each carbon position in $^{13}C_5,^{15}N$-L-Pro-Gly (100 mM) for ΔDP_{sat} experiments at 150 K and 18 kHz MAS frequency. A biexponential fit was necessary for all signals, resulting in four parameters: the time constants of the fast (t_f) and slow (t_s) build-up components, along with their corresponding plateau integrals, A_f and A_s. Additionally, the respective build-up rates are given as $R_f = 1/t_f$ and $R_s = 1/t_s$, as well as the sensitivities $\sigma_f = A_f/\text{sqrt}(t_f)$ and $\sigma_s = A_s/\text{sqrt}(t_s)$.

Label	A_f / a.u.	t_f / s	A_s / a.u.	t_s / s	R_f / 1/s	R_s / 1/s	σ_f / a.u.	σ_s / a.u.
**CO**	489417.164	12.632	627086.08	204.411	0.07916	0.00489	137702.916	43860.647
**Cα**	457917.398	12.294	577721.551	187.549	0.08134	0.00533	130599.207	42185.305
**Cδ**	501111.168	12.094	559561.738	188.054	0.08269	0.00532	144095.062	40804.374
**Cβ**	448675.622	11.39	507211.3	181.388	0.0878	0.00551	132944.572	37660.375
**Cγ**	512429.861	13.393	620402.694	275.524	0.07467	0.00363	140021.748	37376.098

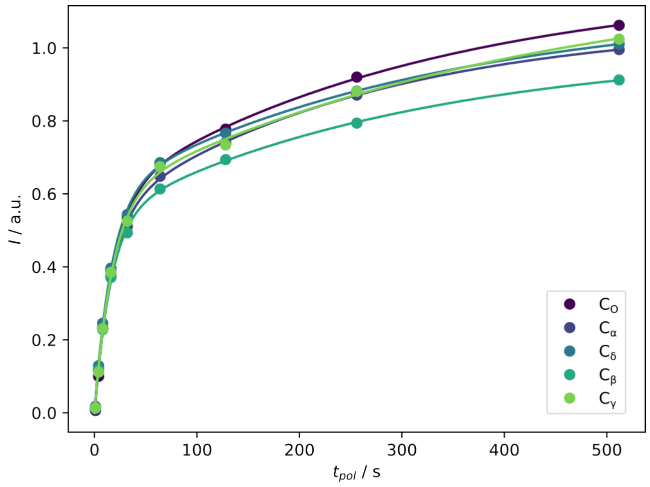

Figure S4.5: Polarization build-up dynamics for each carbon position in $^{13}C_5$,^{15}N-L-Pro-Gly (0.1 M) with 10 mM AMUPol in DNP matrix (glycerol-d$_8$:D_2O:H_2O, 60:30:10 vol.-%) at 150 K and 18 kHz MAS frequency. Solid lines represent biexponential fits with their parameters provided in Table S4.5.

S4.6. $^{13}C_5$,^{15}N-L-Pro-L-Ala at 100 K

Table S4.6: Polarization build-up parameters for each carbon position in $^{13}C_5$,^{15}N-L-Pro-L-Ala (100 mM) for ΔDP_{sat} experiments at 100 K and 18 kHz MAS frequency. A biexponential fit was necessary for all signals, resulting in four parameters: the time constants of the fast (t_f) and slow (t_s) build-up components, along with their corresponding plateau integrals, A_f and A_s. Additionally, the respective build-up rates are given as $R_f = 1/t_f$ and $R_s = 1/t_s$, as well as the sensitivities $\sigma_f = A_f/\mathrm{sqrt}(t_f)$ and $\sigma_s = A_s/\mathrm{sqrt}(t_s)$.

**Label**	**A_f / a.u.**	**t_f / s**	**A_s / a.u.**	**t_s / s**	**R_f / 1/s**	**R_s / 1/s**	**σ_f / a.u.**	**σ_s / a.u.**
**CO**	185986.774	14.214	271988.212	184.6	0.07035	0.00542	49331.453	20018.625
**Cα**	182053.545	12.258	299115.829	226.856	0.08158	0.00441	51998.322	19859.315
**Cδ**	196353.451	13.487	250679.843	212.078	0.07415	0.00472	53466.39	17213.596
**Cβ**	186677.497	13.787	262079.445	239.253	0.07253	0.00418	50275.579	16943.544
**Cγ**	166772.155	13.139	204737.226	167.235	0.07611	0.00598	46008.957	15831.907

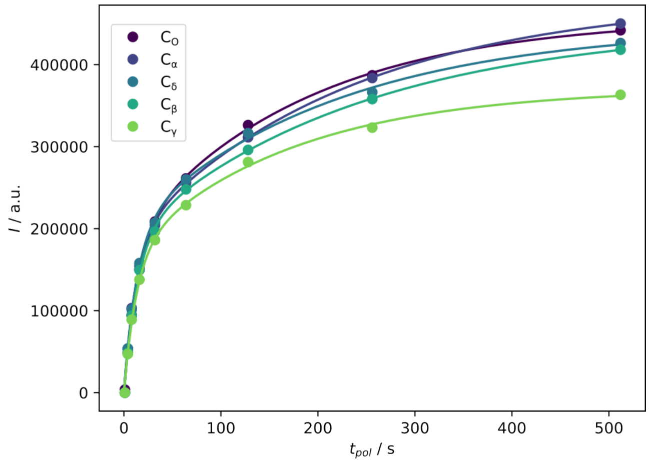

Figure S4.6: Polarization build-up dynamics for each carbon position in $^{13}C_5$,^{15}N-L-Pro-L-Ala (0.1 M) with 10 mM AMUPol in DNP matrix (glycerol-d$_8$:D_2O:H_2O, 60:30:10 vol.-%) at 100 K and 18 kHz MAS frequency. Solid lines represent biexponential fits with their parameters provided in Table S4.6.

S4.7. $^{13}C_5$,^{15}N-L-Pro-L-Ala at 150 K

Table S4.7: Polarization build-up parameters for each carbon position in $^{13}C_5$,^{15}N-L-Pro-L-Ala (100 mM) for ΔDP$_{sat}$ experiments at 150 K and 18 kHz MAS frequency. A biexponential fit was necessary for all signals, resulting in four parameters: the time constants of the fast (t_f) and slow (t_s) build-up components, along with their corresponding plateau integrals, A_f and A_s. Additionally, the respective build-up rates are given as $R_f = 1/t_f$ and $R_s = 1/t_s$, as well as the sensitivities $\sigma_f = A_f/\text{sqrt}(t_f)$ and $\sigma_s = A_s/\text{sqrt}(t_s)$.

Label	A_f / a.u.	t_f / s	A_s / a.u.	t_s / s	R_f / 1/s	R_s / 1/s	σ_f / a.u.	σ_s / a.u.
**CO**	185793.316	14.089	274478.723	187.228	0.07098	0.00534	49498.268	20059.647
**Cα**	169930.182	12.444	260610.79	222.267	0.08036	0.0045	48171.539	17480.542
**Cδ**	202098.69	13.385	253938.819	210.322	0.07471	0.00475	55240.082	17510.024
**Cβ**	170309.586	14.381	220472.825	251.174	0.06954	0.00398	44910.155	13911.3
**Cγ**	182056.06	11.823	260606.36	171.718	0.08458	0.00582	52946.992	19887.358

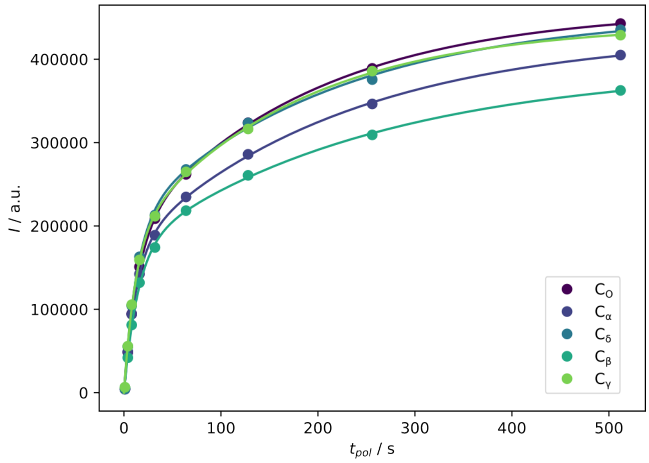

Figure S4.7: Polarization build-up dynamics for each carbon position in $^{13}C_5$,^{15}N-L-Pro-L-Ala (0.1 M) with 10 mM AMUPol in DNP matrix (glycerol-d$_8$:D_2O:H_2O, 60:30:10 vol.-%) at 150 K and 18 kHz MAS frequency. Solid lines represent biexponential fits with their parameters provided in Table S4.7.

S4.8. Gly-$^{13}C_5$,^{15}N-L-Pro at 100 K

Table S4.8: Polarization build-up parameters for each carbon position in Gly-$^{13}C_5$,^{15}N-L-Pro (100 mM) for ΔDP$_{sat}$ experiments at 100 K and 18 kHz MAS frequency. A biexponential fit was necessary for all signals, resulting in four parameters: the time constants of the fast (t_f) and slow (t_s) build-up components, along with their corresponding plateau integrals, A_f and A_s. Additionally, the respective build-up rates are given as $R_f = 1/t_f$ and $R_s = 1/t_s$, as well as the sensitivities $\sigma_f = A_f/\text{sqrt}(t_f)$ and $\sigma_s = A_s/\text{sqrt}(t_s)$. Values given in red are not used to calculate mean values due to the overlap with glycerol signals.

Label	A_f / a.u.	t_f / s	A_s / a.u.	t_s / s	R_f / 1/s	R_s / 1/s	σ_f / a.u.	σ_s / a.u.
**CO**	88573.353	23.057	250083.351	817.011	0.04337	0.00122	18445.978	8749.25
**Cα**	81386.34	17.665	350851.053	511.355	0.05661	0.00196	19363.983	15515.348
**Cδ**	53306.181	13.849	137418.69	200.099	0.07221	0.005	14324.134	9714.565
**Cβ**	95967.75	20.385	189681.651	391.853	0.04906	0.00255	21255.433	9582.167
**Cγ**	50928.278	12.962	196804.227	448.546	0.07715	0.00223	14145.652	9292.465

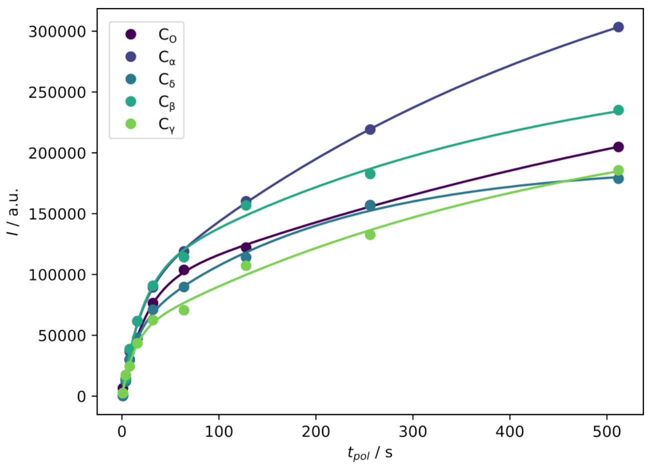

Figure S4.8: Polarization build-up dynamics for each carbon position in Gly-$^{13}C_5$,^{15}N-L-Pro (0.1 M) with 10 mM AMUPol in DNP matrix (glycerol-d_8:D_2O:H_2O, 60:30:10 vol.-%) at 100 K and 18 kHz MAS frequency. Solid lines represent biexponential fits with their parameters provided in Table S4.8.

S4.9. Gly-$^{13}C_5$,^{15}N-L-Pro at 150 K

Table S4.9: Polarization build-up parameters for each carbon position in Gly-$^{13}C_5$,^{15}N-L-Pro (100 mM) for ΔDP_{sat} experiments at 150 K and 18 kHz MAS frequency. A biexponential fit was necessary for all signals, resulting in four parameters: the time constants of the fast (t_f) and slow (t_s) build-up components, along with their corresponding plateau integrals, A_f and A_s. Additionally, the respective build-up rates are given as R_f = 1/t_f and R_s = 1/t_s, as well as the sensitivities σ_f = A_f/sqrt(t_f) and σ_s = A_s/sqrt(t_s). Values given in red are not used to calculate mean values due to the overlap with glycerol signals.

**Label**	A_f **/ a.u.**	t_f **/ s**	A_s **/ a.u.**	t_s **/ s**	R_f **/ 1/s**	R_s **/ 1/s**	σ_f **/ a.u.**	σ_s **/ a.u.**
**CO**	245923.384	10.57	265506.217	131.41	0.09461	0.00761	75641.883	23161.17
**Cα**	260320.382	12.58	525027.588	211.832	0.07949	0.00472	73395.233	36073.339
**Cδ**	250777.217	11.783	231958.992	205.932	0.08487	0.00486	73056.715	16164.017
**Cβ**	204640.441	10.607	205373.067	127.96	0.09428	0.00781	62834.068	18155.423
**Cγ**	281483.165	10.863	272992.327	174.81	0.09206	0.00572	85403.867	20647.492

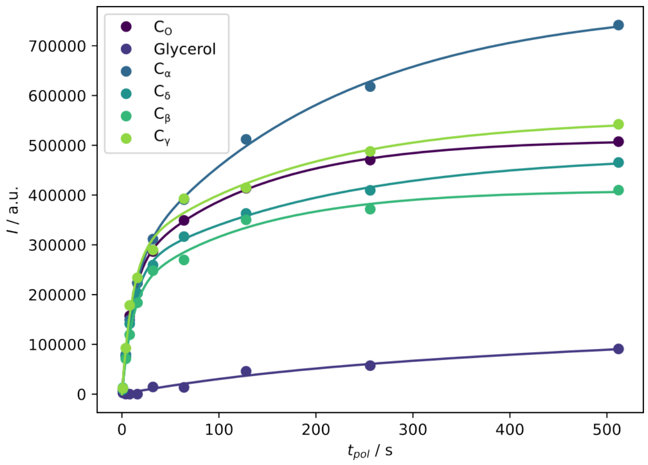

Figure S4.9: Polarization build-up dynamics for each carbon position in Gly-$^{13}C_5$,^{15}N-L-Pro (0.1 M) with 10 mM AMUPol in DNP matrix (glycerol-d_8:D_2O:H_2O, 60:30:10 vol.-%) at 150 K and 18 kHz MAS frequency. Solid lines represent biexponential fits (or exponential fit in case of glycerol) with their parameters provided in Table S4.9.

S4.10. L-Ala-$^{13}C_5$,^{15}N-L-Pro at 100 K

Table S4.10: Polarization build-up parameters for each carbon position in L-Ala-$^{13}C_5$,^{15}N-L-Pro (100 mM) for ΔDP_{sat} experiments at 100 K and 18 kHz MAS frequency. A biexponential fit was necessary for all signals, resulting in four parameters: the time constants of the fast (t_f) and slow (t_s) build-up components, along with their corresponding plateau integrals, A_f and A_s. Additionally, the respective build-up rates are given as R_f = 1/t_f and R_s = 1/t_s, as well as the sensitivities σ_f = A_f/sqrt(t_f) and σ_s = A_s/sqrt(t_s). Values given in red are not used to calculate mean values due to the overlap with glycerol signals.

Label	A_f / a.u.	t_f / s	A_s / a.u.	t_s / s	R_f / 1/s	R_s / 1/s	σ_f / a.u.	σ_s / a.u.
**CO**	45071.982	31.742	209708.752	1536.0	0.0315	0.00065	7999.991	5350.827
**Cα**	22536.896	9.68	101387.201	262.942	0.10331	0.0038	7243.633	6252.492
**Cδ**	30778.491	11.024	80017.13	184.054	0.09071	0.00543	9269.957	5898.076
**Cβ**	36156.767	17.513	125201.229	477.035	0.0571	0.0021	8639.912	5732.36
**Cγ**	28939.851	20.108	63414.299	190.198	0.04973	0.00526	6453.746	4598.162

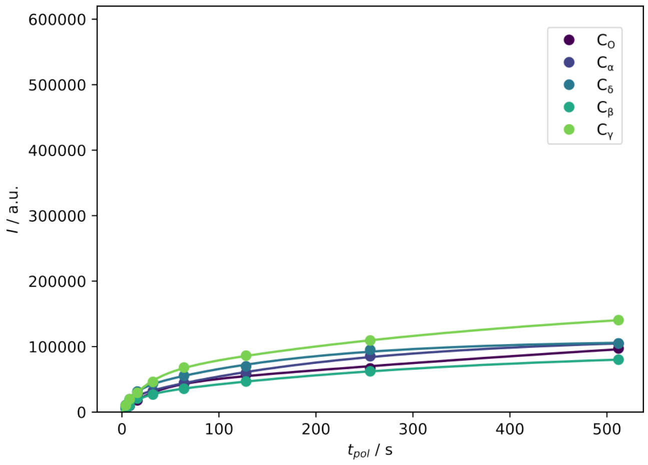

Figure S4.10: Polarization build-up dynamics for each carbon position in L-Ala-$^{13}C_5$,^{15}N-L-Pro (0.1 M) with 10 mM AMUPol in DNP matrix (glycerol-d_8:D_2O:H_2O, 60:30:10 vol.-%) at 100 K and 18 kHz MAS frequency. Solid lines represent biexponential fits with their parameters provided in Table S4.10.

S4.11. L-Ala-$^{13}C_5$,^{15}N-L-Pro at 150 K

Table S4.11: Polarization build-up parameters for each carbon position in L-Ala-$^{13}C_5$,^{15}N-L-Pro (100 mM) for ΔDP_{sat} experiments at 150 K and 18 kHz MAS frequency. A biexponential fit was necessary for all signals, resulting in four parameters: the time constants of the fast (t_f) and slow (t_s) build-up components, along with their corresponding plateau integrals, A_f and A_s. Additionally, the respective build-up rates are given as R_f = 1/t_f and R_s = 1/t_s, as well as the sensitivities σ_f = A_f/sqrt(t_f) and σ_s = A_s/sqrt(t_s). Values given in red are not used to calculate mean values due to the overlap with glycerol signals.

Label	A_f / a.u.	t_f / s	A_s / a.u.	t_s / s	R_f / 1/s	R_s / 1/s	σ_f / a.u.	σ_s / a.u.
**CO**	245923.384	10.57	265506.217	131.41	0.09461	0.00761	75641.883	23161.17
**Cα**	260320.382	12.58	525027.588	211.832	0.07949	0.00472	73395.233	36073.339
**Cδ**	250777.217	11.783	231958.992	205.932	0.08487	0.00486	73056.715	16164.017
**Cβ**	204640.441	10.607	205373.067	127.96	0.09428	0.00781	62834.068	18155.423
**Cγ**	281483.165	10.863	272992.327	174.81	0.09206	0.00572	85403.867	20647.492

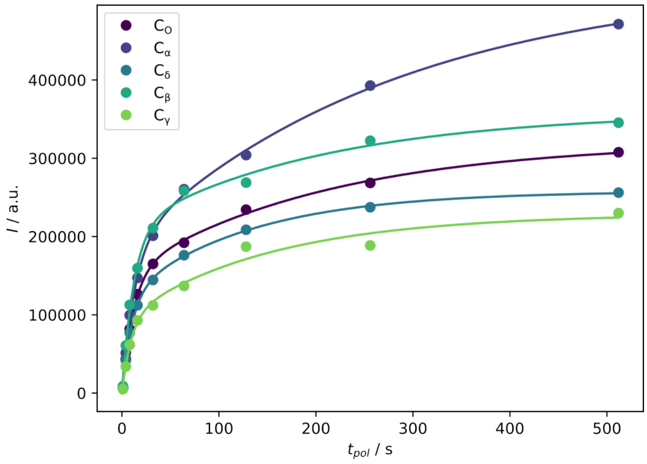

Figure S4.11: Polarization build-up dynamics for each carbon position in L-Ala-$^{13}C_5$,^{15}N-L-Pro (0.1 M) with 10 mM AMUPol in DNP matrix (glycerol-d_8:D_2O:H_2O, 60:30:10 vol.-%) at 150 K and 18 kHz MAS frequency. Solid lines represent biexponential fits with their parameters provided in Table S4.11.

S4.12. L-Pro at 150 K

Table S4.12: Polarization build-up parameters for each carbon position in L-Pro (1 M) for ΔDP_{sat} experiments at 150 K and 18 kHz MAS frequency. A biexponential fit was necessary for all signals, resulting in four parameters: the time constants of the fast (t_f) and slow (t_s) build-up components, along with their corresponding plateau integrals, A_f and A_s. Additionally, the respective build-up rates are given as $R_f = 1/t_f$ and $R_s = 1/t_s$, as well as the sensitivities $\sigma_f = A_f/\mathrm{sqrt}(t_f)$ and $\sigma_s = A_s/\mathrm{sqrt}(t_s)$.

Label	A_f / a.u.	t_f / s	A_s / a.u.	t_s / s	R_f / 1/s	R_s / 1/s	σ_f / a.u.	σ_s / a.u.
**Cδ**	20609.069	7.171	48939.556	43.868	0.13945	0.0228	7696.061	7389.008
**Cβ**	36648.583	9.6	36460.247	170.752	0.10417	0.00586	11828.279	2790.209
**Cγ**	41925.744	7.428	47032.826	92.58	0.13463	0.0108	15383.134	4888.127
**Cα**	31466.959	64.285	---	---	0.01556	---	3924.641	---

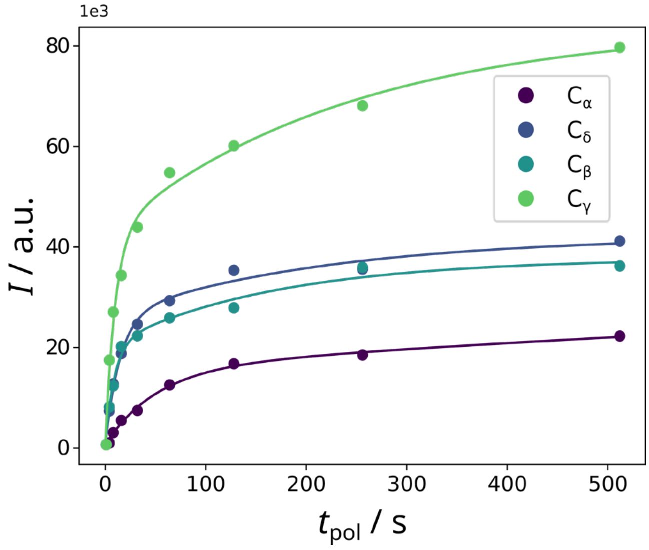

Figure S4.12: Polarization build-up dynamics for each carbon position in L-Pro (1 M) with 10 mM AMUPol in DNP matrix (glycerol-d_8:D_2O:H_2O, 60:30:10 vol.-%) at 100 K and 18 kHz MAS frequency. Solid lines represent biexponential fits (or exponential in case of C_α) with their parameters provided in Table S4.12.

S5. Calculation of energy barriers

The initial structures of the proline dipeptides (L-Pro-Gly, L-Pro-L-Ala, Gly-L-Pro, L-Ala-L-Pro) were built using the tleap program accessible through AMBER.[1] The endocyclic torsion angles (χ_1 to χ_5) of the proline ring were systematically changed to generate conformations with different ring puckering parameters, varying in the pseudorotation phase angle P_m from 0° to 360° in 5° steps. Given these P_m values, the endocyclic torsion angles were calculated by $\chi_i = Q_m \cos[P_m + 4\pi(i-2)/5]$ $(i = 1, 2, 3, 4, 5)$ (Table S6.1).

The χ_1 angle was chosen to be the angle about the C_α-C_β bond. The puckering amplitude (Q_m) was set to 38.6° which is the mean value derived from the X-ray structures of Ac-Pro-NHMe and H-Pro-OH described by DeTar *et al.*[2] Down- and up-puckered conformations of the proline ring were defined according to the position of the C_γ atom of the proline residue being either below or above the plane defined by the three atoms C_δ, N, and C_α.

Table S6.1: Initial endocyclic torsion angles for the proline ring along the pseudorotation phase angle.

P_m / °	X_1 / °	X_2 / °	X_3 / °	X_4 / °	X_5 / °
**0**	-31.228	38.600	-31.228	11.928	11.928
**5**	-29.132	38.453	-33.087	15.082	8.683
**10**	-26.814	38.014	-34.693	18.122	5.372
**15**	-24.292	37.285	-36.036	21.023	2.020
**20**	-21.585	36.272	-37.105	23.765	-1.347
**25**	-18.714	34.983	-37.891	26.325	-4.704
**30**	-15.700	33.429	-38.389	28.685	-8.025
**35**	-12.567	31.619	-38.594	30.827	-11.286
**40**	-9.338	29.569	-38.506	32.735	-14.460
**45**	-6.038	27.294	-38.125	34.393	-17.524
**50**	-2.693	24.812	-37.453	35.789	-20.455
**55**	0.674	22.140	-36.497	36.913	-23.230
**60**	4.035	19.300	-35.263	37.756	-25.828
**65**	7.365	16.313	-33.760	38.312	-28.230
**70**	10.640	13.202	-32.001	38.576	-30.417
**75**	13.833	9.990	-29.998	38.547	-32.373
**80**	16.921	6.703	-27.767	38.224	-34.082
**85**	19.880	3.364	-25.324	37.611	-35.531
**90**	22.689	0.000	-22.689	36.711	-36.711
**95**	25.324	-3.364	-19.880	35.531	-37.611
**100**	27.767	-6.703	-16.921	34.082	-38.224
**105**	29.998	-9.990	-13.833	32.373	-38.547
**110**	32.001	-13.202	-10.640	30.417	-38.576
**115**	33.760	-16.313	-7.365	28.230	-38.312
**120**	35.263	-19.300	-4.035	25.828	-37.756
**125**	36.497	-22.140	-0.674	23.230	-36.913
**130**	37.453	-24.812	2.693	20.455	-35.789
**135**	38.125	-27.294	6.038	17.524	-34.393
**140**	38.506	-29.569	9.338	14.460	-32.735
**145**	38.594	-31.619	12.567	11.286	-30.827
**150**	38.389	-33.429	15.700	8.025	-28.685
**155**	37.891	-34.983	18.714	4.704	-26.325
**160**	37.105	-36.272	21.585	1.347	-23.765
**165**	36.036	-37.285	24.292	-2.020	-21.023
**170**	34.693	-38.014	26.814	-5.372	-18.122

**175**	33.087	-38.453	29.132	-8.683	-15.082
**180**	31.228	-38.600	31.228	-11.928	-11.928
**185**	29.132	-38.453	33.087	-15.082	-8.683
**190**	26.814	-38.014	34.693	-18.122	-5.372
**195**	24.292	-37.285	36.036	-21.023	-2.020
**200**	21.585	-36.272	37.105	-23.765	1.347
**205**	18.714	-34.983	37.891	-26.325	4.704
**210**	15.700	-33.429	38.389	-28.685	8.025
**215**	12.567	-31.619	38.594	-30.827	11.286
**220**	9.338	-29.569	38.506	-32.735	14.460
**225**	6.038	-27.294	38.125	-34.393	17.524
**230**	2.693	-24.812	37.453	-35.789	20.455
**235**	-0.674	-22.140	36.497	-36.913	23.230
**240**	-4.035	-19.300	35.263	-37.756	25.828
**245**	-7.365	-16.313	33.760	-38.312	28.230
**250**	-10.640	-13.202	32.001	-38.576	30.417
**255**	-13.833	-9.990	29.998	-38.547	32.373
**260**	-16.921	-6.703	27.767	-38.224	34.082
**265**	-19.880	-3.364	25.324	-37.611	35.531
**270**	-22.689	0.000	22.689	-36.711	36.711
**275**	-25.324	3.364	19.880	-35.531	37.611
**280**	-27.767	6.703	16.921	-34.082	38.224
**285**	-29.998	9.990	13.833	-32.373	38.547
**290**	-32.001	13.202	10.640	-30.417	38.576
**295**	-33.760	16.313	7.365	-28.230	38.312
**300**	-35.263	19.300	4.035	-25.828	37.756
**305**	-36.497	22.140	0.674	-23.230	36.913
**310**	-37.453	24.812	-2.693	-20.455	35.789
**315**	-38.125	27.294	-6.038	-17.524	34.393
**320**	-38.506	29.569	-9.338	-14.460	32.735
**325**	-38.594	31.619	-12.567	-11.286	30.827
**330**	-38.389	33.429	-15.700	-8.025	28.685
**335**	-37.891	34.983	-18.714	-4.704	26.325
**340**	-37.105	36.272	-21.585	-1.347	23.765
**345**	-36.036	37.285	-24.292	2.020	21.023
**350**	-34.693	38.014	-26.814	5.372	18.122
**355**	-33.087	38.453	-29.132	8.683	15.082
**360**	-31.228	38.600	-31.228	11.928	11.928

The endocyclic torsion angles were adjusted to the values in Table S6.1 by running a minimization calculation with restraints in the Sander program accessible through AMBER.[(1)] The energy minimization calculation used the generalized Born (GB) implicit solvent model, 10000 steps of the steepest descent algorithm, and a force constant for the dihedral angle restraint potentials of 10000 kcal mol^{-1} rad^{-2}. The P_m and Q_m values were back-calculated from the minimized peptide structures according to $\tan P_m = B/A$ and ${Q_m}^2 = A^2 + B^2$ where $A = \frac{2}{5}\sum_{i=1}^{5}\chi_i \cos[4\pi(i-2)/5]$ and $B = -\frac{2}{5}\sum_{i=1}^{5}\chi_i \sin[4\pi(i-2)/5]$. In the case of $\chi_2 < 0°$, the calculated P_m value was augmented by 180°.

The potential energy surfaces of the peptides along P_m were afterwards obtained by adiabatic optimizations and single-point energy calculations of the minimized structures with their endocyclic torsion angles fixed at the initial values using the B3LYP/6-31+G(d,p) level of theory and the IEFPCM implicit water model(3) implemented in the Gaussian 16 program.(4)

S6. References

1. Case DA, Aktulga HM, Belfon K, Cerutti DS, Cisneros GA, Cruzeiro VWD, et al. AmberTools. J Chem Inf Model. 2023 Oct 23;63(20):6183–91. doi:10.1021/acs.jcim.3c01153

2. DeTar DF, Luthra NP. Conformations of proline. J Am Chem Soc. 1977 Feb 1;99(4):1232–44. doi:10.1021/ja00446a040

3. Cancès E, Mennucci B, Tomasi J. A new integral equation formalism for the polarizable continuum model: Theoretical background and applications to isotropic and anisotropic dielectrics. J Chem Phys. 1997 Aug 22;107(8):3032–41. doi:10.1063/1.474659

4. Citation | Gaussian.com [Internet]. [cited 2025 Aug 22]. Available from: https://gaussian.com/citation/
